# Electrophilic compound screening identifies GPX4-dependent ferroptosis as a senescence vulnerability

**DOI:** 10.1038/s41556-026-01921-z

**Published:** 2026-04-24

**Authors:** Mariantonietta D’Ambrosio, Matthew E. H. White, Efthymios S. Gavriil, Laura Bousset, Jodie Birch, Aleksandra Gruevska, Emiliano Pasquini, Manuel Colucci, Winnie Fong, Simone Mosole, Aurora Valdata, Dimitris Veroutis, Katie Tyson, Vikas Ranvir, Sandra Prokosch, Joaquim Pombo, Aoki Ardisson, Sanjay Khadayate, George Young, Alex Montoya, Georgia Roumelioti, Jack Houghton, Jianan Lu, Pavel V. Shliaha, Elena De Vita, Santiago Vernia, Vassilis G. Gorgoulis, Suchira Gallage, Mathias Heikenwälder, Zoe Hall, Andrea Alimonti, Iain A. McNeish, Edward W. Tate, Jesús Gil

**Affiliations:** 1 MRC Laboratory of Medical Sciences, Du Cane Road, London, UK; 2 Institute of Clinical Sciences, Faculty of Medicine, Imperial College London, London, UK; 3 Department of Chemistry, Molecular Sciences Research Hub, Imperial College London, London, UK; 4 The Francis Crick Institute, London, UK; 5 Department of Metabolism, Digestion and Reproduction, Imperial College London, London, UK; 6 Institute of Oncology Research, Bellinzona, Switzerland; 7 Faculty of Biomedical Sciences, Università della Svizzera Italiana, Lugano, Switzerland; 8 University of Tübingen, Faculty of Medicine, Institute for Interdisciplinary Research on Cancer Metabolism and Chronic Inflammation, M3 Research Center for Malignome, Metabolome and Microbiome, Tübingen, Germany; 9 German Cancer Research Center, Division of Chronic Inflammation and Cancer, Heidelberg, Germany; 10 Department of Health Sciences and Technology, ETH Zurich, Zurich, Switzerland; 11 Division of Cancer Research, Ninewells Hospital and Medical School, University of Dundee, Dundee, UK; 12 Ovarian Cancer Action Research Centre, Department of Surgery and Cancer, Imperial College London, London, UK; 13 Centre for Molecular Cell Biology, Department of Biochemistry, School of Biological and Behavioural Sciences, Queen Mary University of London, London, UK; 14 Institute of Biomedicine of Valencia, CSIC and Valencia Biomedical Research Foundation, Centro de Investigación Príncipe Felipe—associated unit to the IBV-CSICC, Valencia, Spain; 15 Molecular Carcinogenesis Group, Department of Histology and Embryology, Medical School, National and Kapodistrian University of Athens, Athens, Greece; 16 Biomedical Research Foundation, Academy of Athens, Athens, Greece; 17 Faculty Institute for Cancer Sciences, Manchester Academic Health Sciences Centre, University of Manchester, Manchester, UK

**Keywords:** Senescence, Cell death, Cancer

## Abstract

Senescent cells drive ageing and age-related pathologies, including cancer. Consequently, senolytics, drugs that selectively kill senescent cells, have broad therapeutic appeal. Here we report a senolytic screen of a library of 10,480 electrophilic compounds. Among 38 identified hits, we found a subset of chloroacetamides with broad senolytic activity. Activity-based protein profiling, coupled with functional assays, identified the glutathione peroxidase GPX4 as a target. We show that senescent cells are primed for ferroptosis, displaying high levels of oxidative stress and intracellular Fe^2+^, but also upregulate GPX4, which prevents the accumulation of oxidized lipids. Treatment with senolytic chloroacetamides or GPX4 inhibitors selectively kills senescent cells by ferroptosis. The combination of anticancer therapies with GPX4 inhibitors eliminated senescent tumour cells in models of melanoma, prostate and ovarian cancer. Our results show that senescent cells rely on GPX4 to prevent ferroptosis and that GPX4 inhibitors kill senescent cells.

## Abstract (web summary)

D’Ambrosio et al. screen 10,480 electrophilic compounds for senolytic effects and show that senolytic chloroacetamides or GPX4 inhibitors selectively kill senescent cells by ferroptosis.

## Main

Cells that undergo senescence stably exit the cell cycle and display distinct phenotypic changes, which include the production of a bioactive secretome (the senescence-associated secretory phenotype (SASP)), reprogramming their metabolism and becoming more resistant to cell death^1^. Induction of senescence is a protective response, as it avoids the replication of aged, damaged and cancerous cells. Senescence limits fibrosis^2^ and cancer initiation^3^, maintains normal tissue function^4^ and contributes to the outcome of many anticancer therapies^5,6^. However, lingering senescent cells drive ageing and disease^7^. Consequently, clearing senescent cells (senolysis) improves healthspan, enhances lifespan^8^ and alleviates a wide range of pathologies^9,10^, including cancer^6,11^.

Compounds that selectively kill senescent cells (senolytics) can treat different age-related pathologies^12^. Senolytic agents include natural compounds such as quercetin^13^ or fisetin^14^, β-galactosidase-activated nanoparticles and prodrugs^15–17^ or compounds that target different vulnerabilities of senescent cells, such as HSP90 inhibitors^18^, cardiac glycosides^19,20^ or NMT inhibitors^21^. BCL2 family inhibitors, such as ABT-263 and ABT-737^22–24^, that target BCL2 and BCL-XL, or S63845, an MCL-1 inhibitor^25^, are also senolytic. This is a consequence of senescent cells being primed for apoptosis and upregulating antiapoptotic mechanisms to prevent intrinsic^24^ and extrinsic^26^ apoptosis, which makes them vulnerable to the inhibition of these protective mechanisms. Whether senescent cells also activate mechanisms protecting against other types of cell death is unclear.

Covalent drug discovery has emerged as a powerful approach to target proteins that were considered undruggable. This resurgence has been driven by the discovery of novel tools and technologies that facilitate the rational design of covalent ligands and the identification of their targets^27–29^. The broad appeal of these drugs is showcased by the Food and Drug Administration’s approval of electrophilic drugs that act by forming covalent bonds against cancer-relevant targets such as BTK^30^, EGFR^31^ or KRAS^G12C^ (ref. ^32^).

Here, we describe a senolytic screen using a library of electrophilic compounds. Our screen unveiled 38 chemically diverse electrophiles with senolytic activity. Among these, we identified a subset of senolytic chloroacetamides that target glutathione peroxidase 4 (GPX4). We showed that senescent cells are primed for ferroptosis and that targeting GPX4 unlocks a vulnerability of senescent cells that can be exploited for therapeutic benefit.

## Results

### A phenotypic screen identifies electrophilic compounds with senolytic activity

**Fig. 1:**
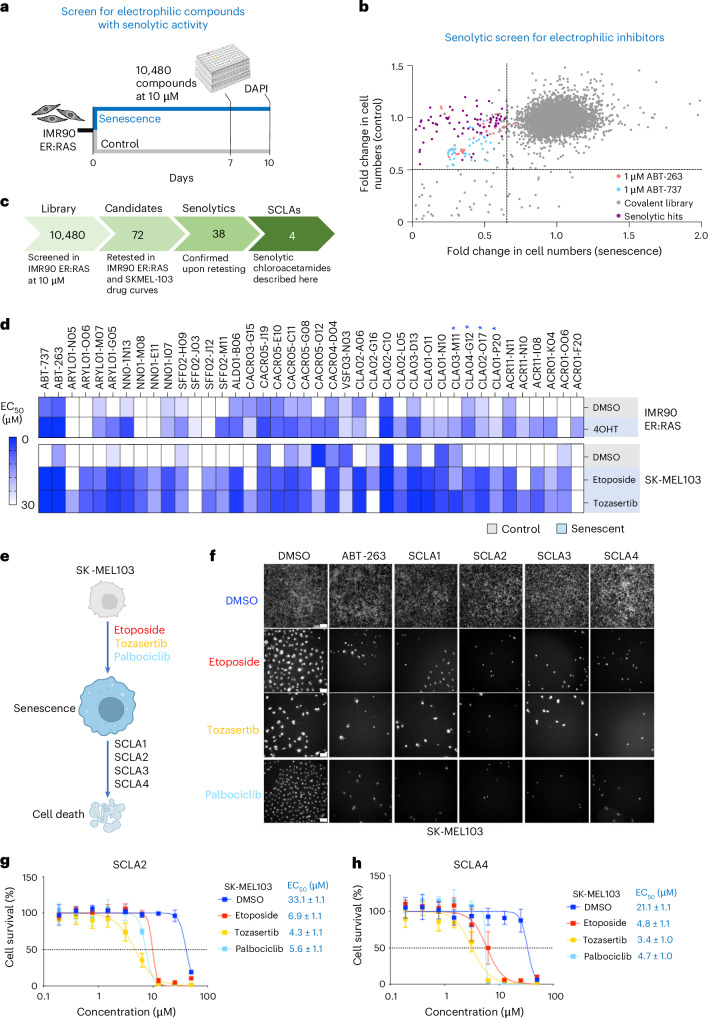
A screen of electrophilic compounds identifies four chloroacetamides with senolytic activity. **a**, Schematic of the senolytic screen in IMR90 ER:RAS cells to identify senolytic electrophilic compounds. **b**, Dot plot showing the screening results of the electrophile library, which contains 10,480 compounds. Purple dots represent the senolytic hits; light blue and pink dots represent the senolytic drugs used as controls (ABT-263 and ABT-737). The screen data are presented in Supplementary Table 1. **c**, Summary of the electrophile library screen in IMR90 ER:RAS and retest in IMR90 ER:RAS and SK-MEL-103. **d**, Heat map showing the EC_50_ of the 38 senolytic hits in non-senescent and senescent IMR90 ER:RAS and SK-MEL-103. Blue asterisks indicate the four senolytic chloroacetamides described in this study. Retesting data are presented in Supplementary Table 2. **e**, Schematic of the senolytic assay performed with the four selected chloroacetamides (SCLA1, SCLA2, SCLA3 and SCLA4) in SK-MEL-103 cells. **f**, Representative DAPI-stained images showing cell numbers after treatment with the four chloroacetamides in senescent and non-senescent cells. ABT-263 is shown as a positive control. Scale bar, 100 μm (*n* = 4 independent experiments for SCLA2 and SCLA4; *n* = 3 independent experiments for SCLA1, SCLA3 and ABT-263). **g**,**h**, Dose–response curves of SCLA2 (**g**) and SCLA4 (**h**) in SK-MEL-103 cells treated with DMSO, etoposide, tozasertib, or palbociclib for 6 days. Data represent mean ± s.d. (*n* = 4 biological replicates for SCLA2 and SCLA4). EC_50_ is obtained by calculating the geometric mean of the single replicate EC_50._ The standard error is calculated on the log-transformed data, back-transformed to the original scale. Schematic in **e** created in BioRender; D’Ambrosio, M. https://biorender.com/kagkyla (2025). Source data

**Extended Data Fig. 1:**
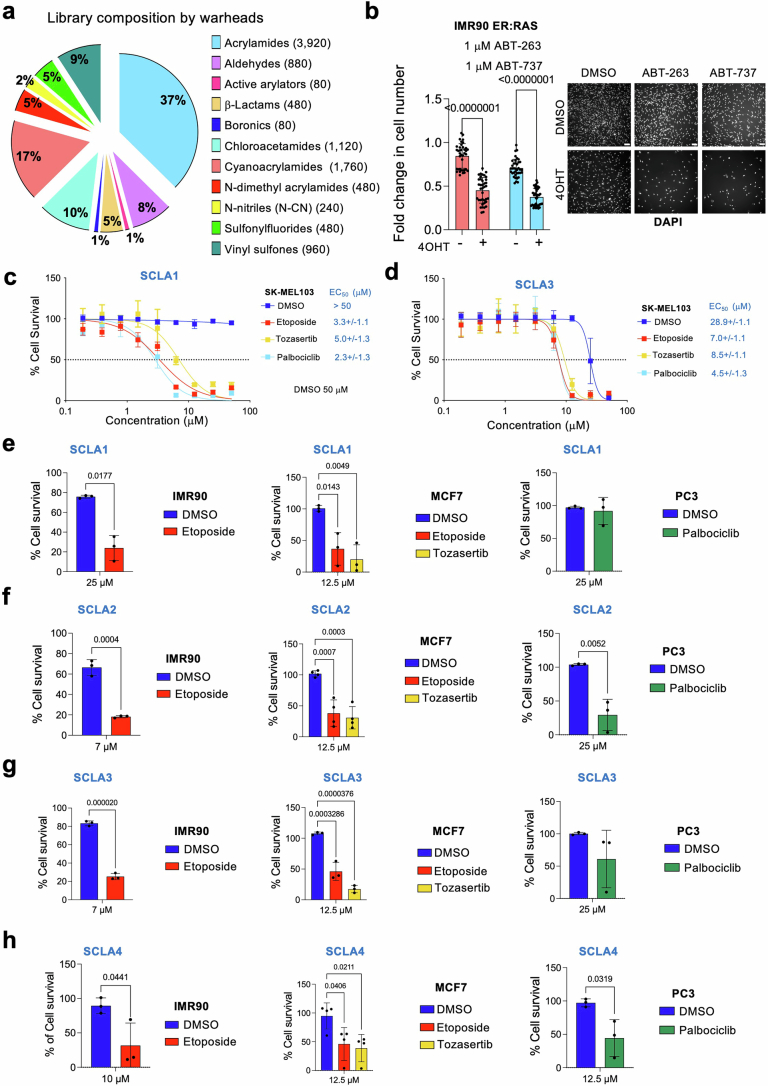
Chloroacetamides identified in the screen are senolytic in different cell models. **a**, Pie chart showing the warhead composition of the ‘Covalent Screening library’ of electrophilic compounds. **b**, Representative images (right panel) and quantification (left panel) of ABT-263 and ABT-737. The killing of senescent cells was measured by normalising the cell count of senescent IMR90 ER:RAS (treated with 4OHT) with the non-senescent counterpart (DMSO). Cell count was determined by DAPI staining. Scale bar, 100 μm. Data represent mean ± SD (n= 38 biological replicates). Statistical test used: two-way ANOVA (Dunnett’s multiple comparisons test). **c-d**, Dose-response curves of SCLA1 (**c**) and SCLA3 (**d**) in SK-MEL103 cells treated with DMSO, Etoposide, Tozasertib, or Palbociclib for 6 days. Data represent mean ± SD (n = 3 biological replicates for SCLA1 and SCLA3). EC_50_ is obtained by calculating the geometric mean of the single replicate EC_50._ The standard error is calculated on the log-transformed data, back-transformed to the original scale. **e-h**, Bar graphs showing the senolytic effects of SCLA1 (**e**), SCLA2 (**f**), SCLA3 (**g**) and SCLA4 (**h**) in different models of therapy-induced senescence (TIS). Data represent mean ± SD (n = 3 biological replicates). Statistical test used: unpaired t-test (two-tailed) for IMR90 and PC3, one-way ANOVA (Dunnett’s multiple comparisons test) for MCF-7. Source data

To identify vulnerabilities of senescent cells, we took advantage of a library of electrophilic compounds (Methods) to conduct a senolytic screen (Fig. 1a and Extended Data Fig. 1a). We screened IMR90 primary human lung fibroblasts expressing an ER:RAS fusion protein, a model of oncogene-induced senescence (OIS)^19,21,33^. Treatment with 4-hydroxytamoxifen (4-OHT) activates ER:RAS, inducing senescence (Supplementary Fig. 1). Senolytic drugs such as the BCL2 family inhibitors ABT-263 and ABT-737^23,24,34^ selectively killed senescent IMR90 ER:RAS cells when compared with non-senescent controls (Extended Data Fig. 1b).

The library comprised 10,480 electrophilic compounds with diverse warheads that target cysteines (for example, acrylamides and chloroacetamides) or other nucleophilic residues (for example, aldehydes and boronic acids) (Extended Data Fig. 1a). In the screen, we compared the effect that electrophiles (at 10 μM) had on the viability of senescence and normal cells, identifying 72 candidates with senolytic activity (Fig. 1b,c and Supplementary Table 1). We retested these compounds in IMR90 ER:RAS cells, and in a melanoma cell line, SK-MEL-103, in which we induced senescence by treatment with the chemotherapeutic agent etoposide or the Aurora kinase inhibitor tozasertib (Supplementary Fig. 2). We calculated the half maximal effective concentration (EC_50_) across dose–response series in control and senescent cells. The senolytic effects varied between inducers and cell lines, but we identified 38 fragments (with diverse warheads, including chloroacetamides, cyanoacrylamides, acrylamides, aldehydes or sulfonyl fluorides) that selectively killed senescent cells in at least one of the three senescent conditions analysed (Fig. 1c,d, and Supplementary Table 2)

### Characterizing four senolytic chloroacetamides

Among the electrophilic compounds with senolytic activity identified, we concentrated on four senolytic chloroacetamides (SCLAs; Supplementary Fig. 3), which we chose on the basis of their chemical structure and the strength of their senolytic effects. The four senolytic chloroacetamides (referred to as SCLA1, SCLA2, SCLA3 and SCLA4 herein) selectively killed SK-MEL-103 cells undergoing senescence triggered by etoposide, tozasertib or palbociclib (a CDK4/6 inhibitor) (Fig. 1e–h, Extended Data Fig. 1c,d and Supplementary Fig. 4). For example, when comparing their EC_50_, SK-MEL-103 cells undergoing palbociclib-induced senescence were killed using 20-fold lower concentrations of SCLA1 (Extended Data Fig. 1c). The four SCLAs were also senolytic against a wide range of senescent cells, including IMR90 cells treated with etoposide, MCF7 breast cancer cells treated with either etoposide or tozasertib and PC3 prostate cancer cells treated with palbociclib (Extended Data Fig. 1e–h and Supplementary Fig. 5). In conclusion, the above experiments confirm the identification of four distinct chloroacetamide chemotypes with broad senolytic activity.

### Combining pulldown experiments and functional assays to identify senolytic targets

**Fig. 2:**
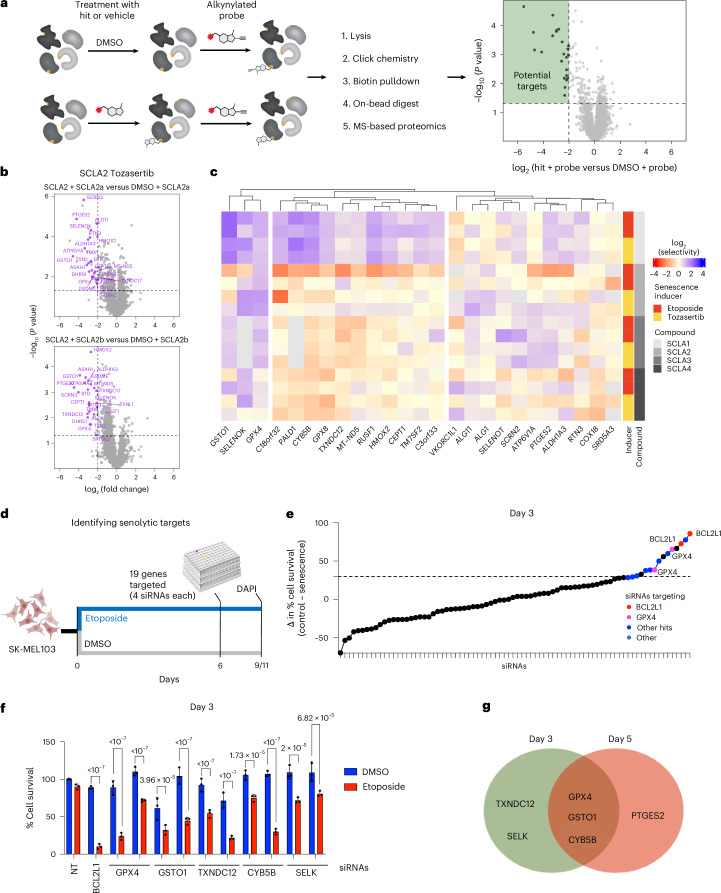
Pulldown experiments identify senolytic targets of chloroacetamides. **a**, Schematic of competitive protein-level ABPP (pulldown) experiments for the identification of potential target proteins. For all described MS-based ABPP experiments, cells were treated with the compound or probe for 2 h. **b**, Volcano plots showing proteins pulled down by SCLA2a (top) and SCLA2b (bottom) and significantly (log_2_(fold change) <−2, *P* value <0.05, Welch’s *t*-test) competed by SCLA2, respectively, in tozasertib-treated SK-MEL-103 cells (*n* = 3 or four biological replicates). Purple dots represent significantly competed proteins by SCLA2 from both SCLA2-derived probe experiments. Statistical test used: Welch’s *t*-test (two-tailed). **c**, Heat map showing the extent of senescence-selective competition of the four chloroacetamides for 25 commonly liganded proteins. Tile colour represents the fold difference in competition for each compound–protein pair in etoposide/tozasertib-treated SK-MEL-103 cells compared with non-senescent SK-MEL-103 cells. Selectivity profiles for each protein were subjected to unsupervised hierarchical clustering by Euclidean distance and grouped by cluster. The MS data are presented in Supplementary Table 4. **d**, Schematic of the siRNA senolytic screen performed in SK-MEL-103 cells. **e**, Dot plot showing the results of siRNA senolytic screen. Pink dots represent GPX4 siRNA, blue dots represent other senolytic siRNA and red dots represent BCL2L1 siRNA used as a positive control. **f**, Bar graph showing the percentage of cell survival of senescent and non-senescent SK-MEL-103 cells after 3 days of transfection with the different siRNAs. Data represent mean ± s.d. (*n* = 3 biological replicates). Statistical test used: two-way ANOVA (Šídák’s multiple comparisons test). **g**, Venn diagram summarizing the senolytic siRNAs 3 or 5 days after transfection in SK-MEL-103 cells. Schematic in **e** created in BioRender; D’Ambrosio, M. https://biorender.com/33cxkim (2025). Source data

**Extended Data Fig. 2:**
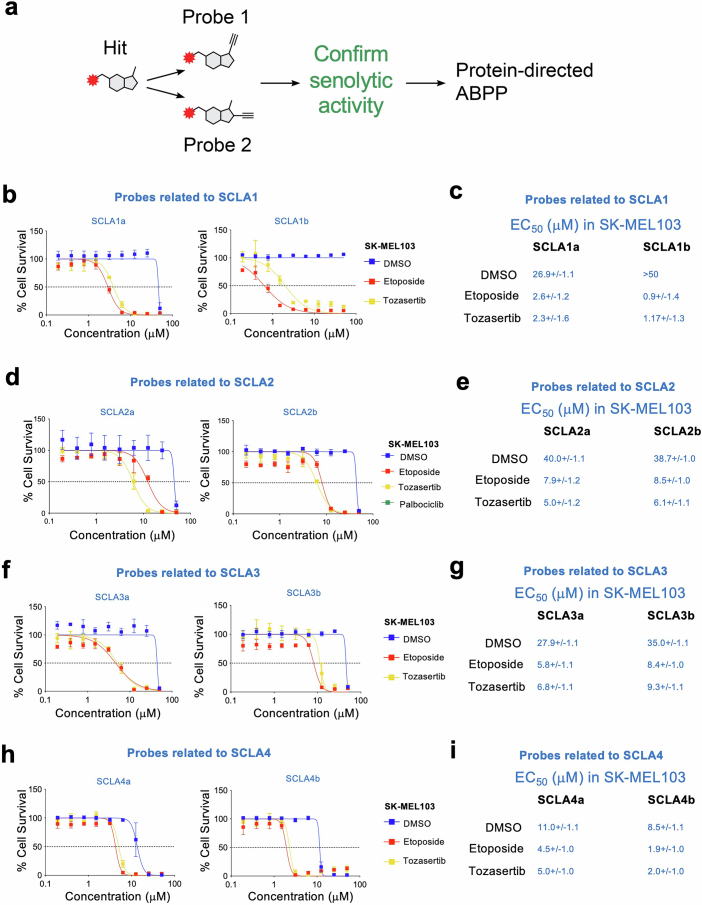
Probes retain senolytic activity. **a**, Schematic of the use of alkynylated chemical probes derived from SCLAs and subsequent in vitro testing. **b**, Dose-response curves of SCLA1a and SCLA1b in SK-MEL103 cells treated with DMSO, Etoposide, or Tozasertib. Data represent mean ± SD (n = 4 for SCLA1a and n = 3 for SCLA1b independent experiments). **c**, Table summarizing EC50 (μM) of the probes related to SCLA1 (SCLA1a and SCLA1b). **d**, Dose-response curves of SCLA2a and SCLA2b in SK-MEL103 cells treated with DMSO, Etoposide, or Tozasertib for 6 days. Data represent mean ± SD (n = 3 independent experiments). **e**, Table summarizing EC50 (μM) of the probes related to SCLA2 (SCLA2a and SCLA2b) in SK-MEL103 cells. **f**, Dose-response curves of SCLA3a and SCLA3b in SK-MEL103 cells treated with DMSO, Etoposide, or Tozasertib. Data represent mean ± SD (n = 4 for SCLA3a and n = 3 for SCLA3b independent experiments). **g**, Table summarizing EC50 (μM) of the probes related to SCLA3 (SCLA3a and SCLA3b). **h**, Dose-response curves of SCLA4a and SCLA4b in SK-MEL103 cells treated with DMSO, Etoposide, or Tozasertib. Data represent mean ± SD (n = 3 independent experiments). **i**, Table summarizing EC50 (μM) of the probes related to SCLA4 (SCLA4a and SCLA4b). For panels b, d, f, and h, EC50 was obtained by calculating the geometric mean of the single replicate EC50. The standard error is calculated on the log-transformed data, back-transformed to the original scale. Source data

To identify the targets responsible for their senolytic activity, we synthesized two alkynylated derivatives of each compound, which we used to perform target identification in competitive, protein-directed activity-based protein profiling (ABPP) experiments (Fig. 2a). We first confirmed that the addition of alkyne handles to SCLA1-4 did not ablate senolytic activity (Extended Data Fig. 2). Next, we performed competitive ABPP experiments by treating SK-MEL-103 cells first with either an SCLA compound or dimethylsulfoxide (DMSO), followed by treatment with a paired alkyne-modified clickable probe. Using click chemistry, we incorporated a biotin tag that facilitated streptavidin pulldown of proteins covalently modified by our probes, followed by quantitative mass spectrometry (MS) for target identification. Proteins highly engaged by SCLA compounds were less labelled by subsequent treatment with an alkyne-modified probe, thereby showing a reduced signal following click chemistry, streptavidin pulldown and quantitative label-free MS (Fig. 2a).

**Extended Data Fig. 3:**
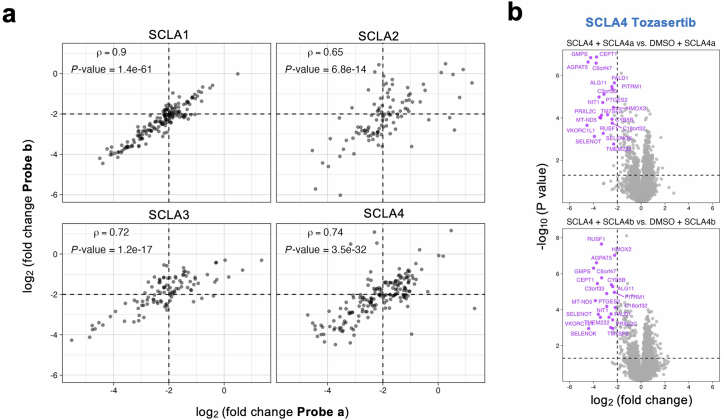
Pulldown experiments. **a**, Scatter plot showing correlation of log2(fold-change) of all proteins with at least one significant competition event (log2(fold-change) < -2; P-value < 0.05, Welch’s t-test) for a given SCLA across non-senescent and senescent SK-MEL103. X- and Y-axes, respectively, show the fold-change for both alkynylated probes derived from each SCLA compound. Spearman’s rank order correlation coefficient (ρ) and P-value are displayed for each SCLA set. (Spearman’s rank order correlation coefficient was calculated for complete fold-change values between both sets of probes. Associated P-values were calculated by asymptotic t-approximation). **b**, Volcano plots showing proteins pulled down by SCLA4a (top) and SCLA4b (bottom) and significantly (log2(fold-change) < -2, P-value < 0.05, Welch’s t-test) competed by SCLA4 respectively in tozasertib-treated SK-MEL103 cells (n = 3 or 4 biological replicates). Purple dots represent significantly competed proteins by SCLA4 from both SCLA4-derived probe experiments. MS data is presented in Table S4. Statistical test used: Welch’s t-test (two-tailed).

We applied this workflow for each of two distinct alkyne-modified probes per SCLA compound across both non-senescent and senescent (etoposide- or tozasertib-induced) SK-MEL-103 cells. We treated with concentrations of SCLA compounds and derived alkyne probes (Supplementary Table 3) at which there was a strong senolytic effect but no cytotoxic effects in non-senescent cells (Fig. 1g,h and Extended Data Figs. 1c,d and 2b–i). Applying stringent thresholds for significant competition (>4-fold reduction in signal on SCLA treatment), we observed good agreement in the target profiles for each SCLA compound with both respective alkyne probes (Fig. 2b, Extended Data Fig. 3 Supplementary Table 4). Consistent with the high reactivity of chloroacetamide warheads and the fragment-like nature of SCLA1-4, we observed a total of 134 proteins that were significantly engaged in at least one ABPP experiment. A further set of proteins commonly engaged in senescent SK-MEL-103 cells emerged, with 25 proteins engaged in >25% of senescence inducers and probes tested. This set included proteins previously established as containing hyper-reactive^35,36^ or commonly liganded^37^ cysteine residues (Supplementary Table 4). We subsequently concentrated on the subset of proteins enriched in pulldowns from senescent cells when compared with non-senescent controls. Hierarchical clustering of commonly engaged proteins by the fold increase in target engagement in senescent versus non-senescent conditions (referred to as ‘senescence selectivity’) identified broadly highly-reactive proteins, clusters of proteins with specificity to certain chemotypes and others (such as GPX4) that were enriched in probes related to three different SCLAs (Fig. 2c).

**Extended Data Fig. 4:**
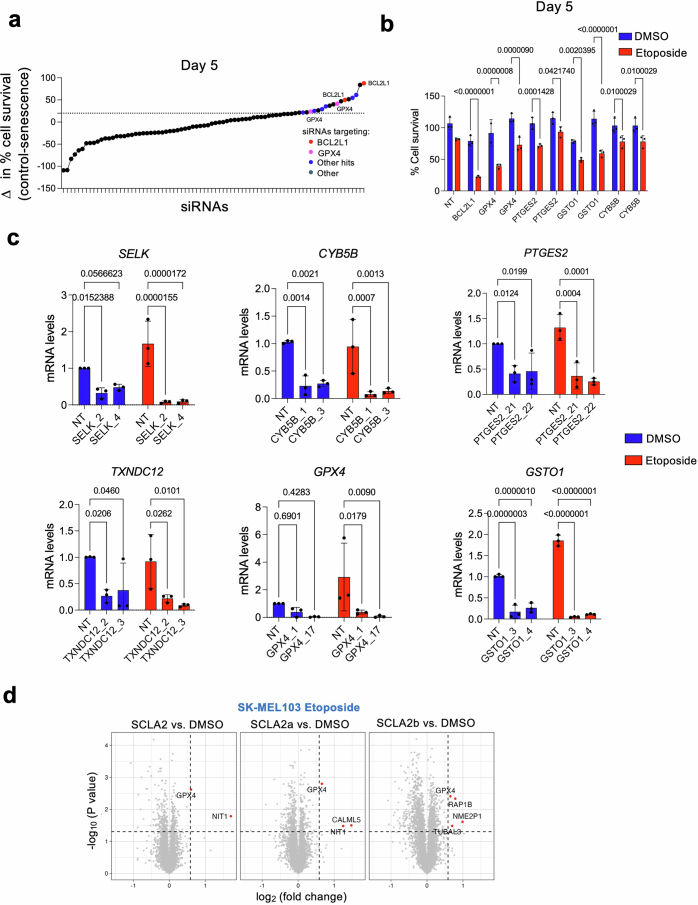
siRNA senolytic screening and siRNA validation. **a**, Dot plot showing the results of siRNA senolytic screen. Pink dots represent GPX4 siRNA, blue dots represent other senolytic siRNA, and red dots represent BCL2L1 siRNA used as a positive control. **b**, Bar graph showing the percentage of cell survival of senescent and non-senescent SK-MEL103 cells after 5 days of transfection with the different siRNAs. Data represent mean ± SD (n = 3 biological replicates). Statistical test used: two-way ANOVA (Šídák’s multiple comparisons test). **c**, Bar graphs showing mRNA levels of *SELK, CYB5B, PTGES2, TXNDC12, GPX4* and *GSTO1* in SK-MEL103 treated with DMSO or Etoposide and transfected with siRNA. Data represent mean ± SD (n = 3 independent experiments). Statistical test used: two-way ANOVA (Dunnett’s multiple comparisons test). **d**, Whole proteome profiling of senescent (etoposide) SK-MEL103 cells showing protein-level changes upon treatment with SCLA2 (10 mM) or derived probes at (2.5 mM) for 2h. Red dots show proteins with >1.5-fold upregulation and *P*-value < 0.05 (Welch’s t-test, two-tailed, n = 4 biological replicates). MS data is presented in Table S4. Source data

To understand which targets explain the senolytic activity of the SCLAs, we generated a small library of 76 small interfering (si)RNAs (4 siRNAs each against 19 targets). We screened the siRNA target library for its ability to selectively kill SK-MEL-103 cells undergoing etoposide-induced senescence (Fig. 2d). We assessed the survival of SK-MEL-103 either 3 or 5 days after siRNA transfection (Fig. 2e and Extended Data Fig. 4a) and identified five candidate senolytic targets on day 3 and four on day 5 (Fig. 2f and Extended Data Fig. 4b,c). From those, GSTO1, CYB5B and GPX4 were shared (Fig. 2g). Among those, GPX4 was of particular interest. The MS pulldown experiments had identified GPX4 as a target of three out of four SCLAs (SCLA1, SCLA2 and SCLA3; Fig. 2c and Supplementary Table 4). Moreover, GPX4 levels were elevated on treatment with SCLA2 and derived alkyne probes SCLA2a and SCLA2b (Extended Data Fig. 4d). The above results suggested that GPX4 could be a target that explained, at least in part, the senolytic activity of SCLA1, SCLA2 and SCLA3.

### A subset of senolytic chloroacetamides induces ferroptosis by engaging GPX4

**Fig. 3:**
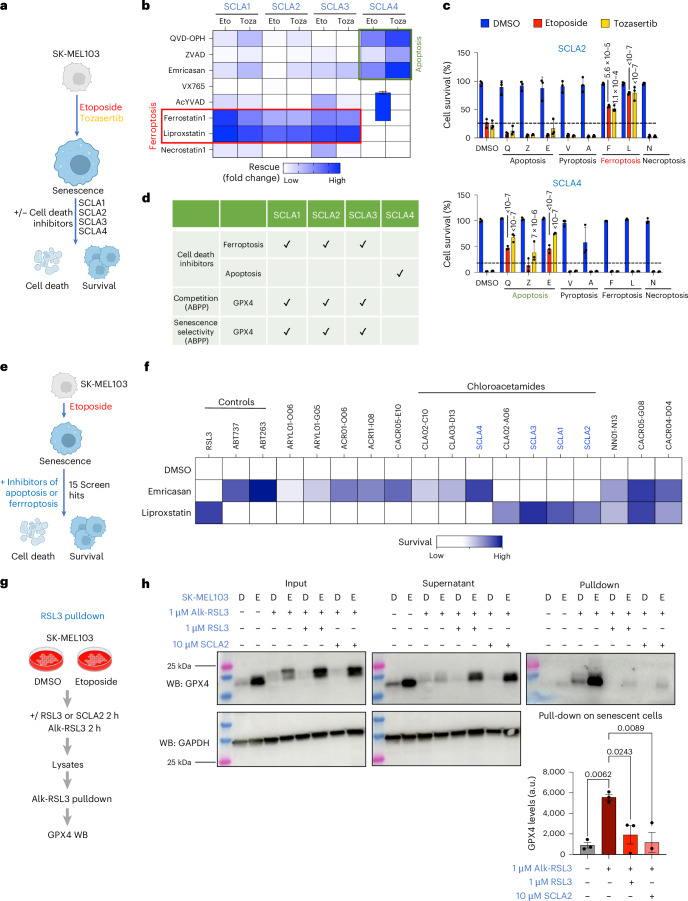
A subset of senolytic chloroacetamides induces ferroptosis by engaging GPX4. **a**, Schematic of senolytic assay performed with or without different cell death inhibitors. **b**, Heat map summarizing the results of the senolytic assay with or without different cell death inhibitors. The rescue was calculated as the % survival of senescent cells after the treatment with the different cell death inhibitors, divided by the % survival with the corresponding SCLA alone. **c**, Bar graphs showing the senolytic activity of SCLA2 and SCLA4 in the presence or absence of different cell death inhibitors. The inhibitors used are: Q-VD-OPh (Q) at 25 μM, Z-VAD-FMK (Z) at 25 μM, emricasan (E) at 25 μM, VX-765 (V) at 25 μM, Ac-YVAD-cmk (A) at 25 μM, ferrostatin 1 (F) at 10 μM, liproxstatin 1 (L) at 1 μM and necrostatin-1 at 10 μM. Data represent mean ± s.d. (*n* = 3 independent experiments). Statistical test used: two-way ANOVA (Dunnett’s multiple comparisons test). **d**, Table summarizing the results from pulldown experiments and senolytic assay with or without different cell death inhibitors. **e**, Schematic of the experiment. **f**, Heat map summarizing the results from the senolytic screen in SK-MEL-103 cells. Represented in the heat map is the % survival of senescent cells treated with electrophilic compounds in the presence of DMSO or cell death inhibitors. **g**, Schematic illustrating the Alk-RSL3 competition experiment performed in SK-MEL-103 cells. **h**, Western blot showing GPX4 levels in senescent and non-senescent SK-MEL-103 cells, either untreated or treated with Alk-RSL3 (1 μM) with or without pre-treatment with RSL3 (1 μM) or SCLA2 (10 μM). ‘Input’ represents the lysates after click chemistry; ‘supernatant’ represents the supernatant collected after pulldown. An immunoblot of GAPDH is included as a loading control. Western blots are a representative experiment out of three independent experiments. GPX4 protein quantification of the pulldown is normalized to the input. Data represent mean ± s.e.m. (*n* = 3 biological replicates). Statistical test used: one-way ANOVA (Tukey’s multiple comparisons test). Schematic created in BioRender: **a** and **e**, D’Ambrosio, M. https://biorender.com/49et4eq (2025); **g**, D’Ambrosio, M. https://biorender.com/dui3y7w. (2025). Source data

**Extended Data Fig. 5:**
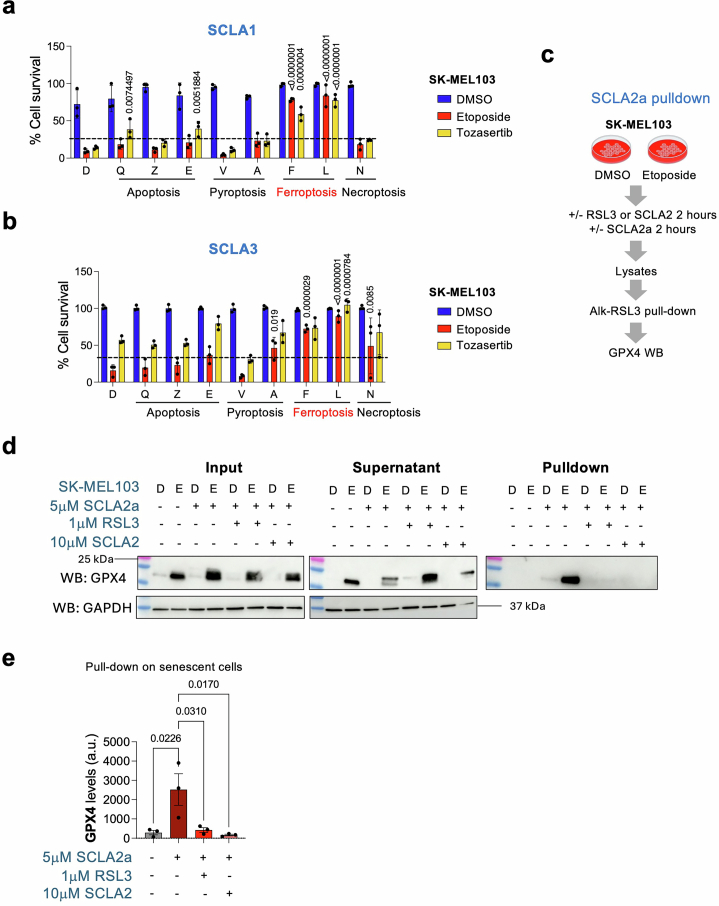
SCLAs induce senolysis by ferroptosis and engage GPX4. **a-b**, Bar graphs showing the senolytic activity of SCLA1 (**a**) and SCLA3 (**b**) in the presence or absence of different cell death inhibitors. The inhibitors used are: Q-VD-OPh (Q) at 25 mM, Z-VAD-FMK (Z) at 25 mM, Emricasan (E) at 25 mM, VX-765 (V) at 25 mM, Ac-YVAD-cmk (A) at 25 mM, Ferrostatin-1 (F) at 10 mM, Liproxstatin-1 (L) at 1 mM and Necrostatin-1 at 10 mM. Data represents mean ± SD (n = 3 independent experiments). Statistical test used: two-way ANOVA (Dunnett’s multiple comparisons test). **c**, Schematic illustrating the SCLA2a competition experiment performed in SK-MEL103 cells. **d**, Western blot showing GPX4 levels in senescent and non-senescent SKMEL-103 cells, either untreated or treated with SCLA2a (5 mM) with or without pre-treatment with SCLA2 (10 mM) or RSL3 (1 mM). “Input” represents the lysates after click chemistry; “supernatant” represents the supernatant collected after pulldown. Immunoblots against GAPDH are included as loading controls. Western blots are a representative experiment out of 3 independent experiments. **e**, Bar graph representing GPX4 pulldown quantification. GPX4 protein quantification of the pulldown is normalised to the input. Data represent mean ± SEM (n = 3 independent experiments). Statistical test used: one-way ANOVA (Tukey’s multiple comparisons test). Schematic in **c** created in BioRender; D’Ambrosio, M. https://biorender.com/dui3y7w (2025). Source data

GPX4 is a selenoprotein with glutathione peroxidase activity, which inhibits ferroptosis^38^. As GPX4 inhibitors such as 1S,3R-RSL3 (hereafter referred to as RSL3)^39^ induce ferroptosis, we investigated which type of cell death was induced by the SCLAs on senescent cells. To this end, we induced senescence in SK-MEL-103 cells and pretreated them with different cell death inhibitors before assessing the effect of SCLA1-4 (Fig. 3a). Inhibitors of pyroptosis (VX-765 or AcYVAD) or necroptosis (necrostatin-1) did not rescue death induced by any of the SCLAs (Fig. 3b,c and Extended Data Fig. 5a,b). Two inhibitors of ferroptosis (ferrostatin 1 and liproxstatin 1) prevented the senolytic effects of SCLA1, SCLA2 and SCLA3, while inhibitors of apoptosis (QVAD, Z-VAD and emricasan) prevented senolysis induced by SCLA4 (Fig. 3b,c and Extended Data Fig. 5a,b). In summary, SCLA1, SCLA2 and SCLA3 induced ferroptosis, which correlated with the identification of GPX4 as a target for these SCLAs in pulldown experiments and senescence selectivity of engagement with these SCLAs. On the contrary, GPX4 was not a target of SCLA4, which induced senolysis by apoptosis (Fig. 3c,d).

As we identified GPX4 as a functional target of three out of four senolytic chloroacetamides, we wanted to understand how widely senolytic electrophilic compounds might induce ferroptosis. Among the 38 hits from the screen, we selected the 15 with a stronger senolytic effect on etoposide-treated SK-MEL-103 cells and, taking advantage of inhibitors of apoptosis (emricasan) or ferroptosis (liproxstatin 1), assessed their mode of action (Fig. 3e). The senolytic effects of three compounds (two cyanoacrylamides and an N-nitrile) were prevented by inhibitors of either apoptosis or ferroptosis. Death induced by the other 12 compounds was prevented only by either treatment with apoptosis inhibitors (8/12) or ferroptosis inhibitors (4/12). Interestingly, the four compounds that induced ferroptosis were SCLA1, SCLA2, SCLA3 and one other chloroacetamide. However, three other chloroacetamides (including SCLA4) induced senolysis by apoptosis. Overall, these results suggest that a subset of chloracetamides with diverse chemical structures engages GPX4 to induce ferroptosis (Fig. 3f).

To confirm that GPX4 was directly targeted by SCLA2, we used an alkynylated probe of the GPX4 covalent inhibitor RSL3 (referred to as Alk-RSL3; Fig. 3g). Alk-RSL3 was conjugated to biotin using click chemistry to perform streptavidin pulldown from SK-MEL-103 extracts. GPX4 was identified by western blot in the pulldown eluate and was depleted from supernatant fractions, confirming that RSL3 binds to GPX4 (Fig. 3h). GPX4 levels were increased in senescent cells (see input), and this correlated with increased GPX4 levels in the pulldown eluates from senescent cells (Fig. 3h). Preincubation with either RSL3 or SCLA2 diminished GPX4 pulldown (Fig. 3h). We performed a similar experiment using one of our alkynylated probes (SCLA2a) with or without preincubation with RSL3 or SCLA2. (Extended Data Fig. 5c). SCLA2a was able to pull down GPX4, and GPX4 pulldown was prevented by preincubation with RSL3 or SCLA2 (Extended Data Fig. 5d,e). These competition experiments suggest that SCLA2 occupies the same site as RSL3 in GPX4. Overall, our results show that a subset of chloroacetamides (including SCLA1, SCLA2 and SCLA3) engages GPX4 to induce ferroptosis in senescent cells.

### GPX4 inhibitors are senolytic

**Fig. 4:**
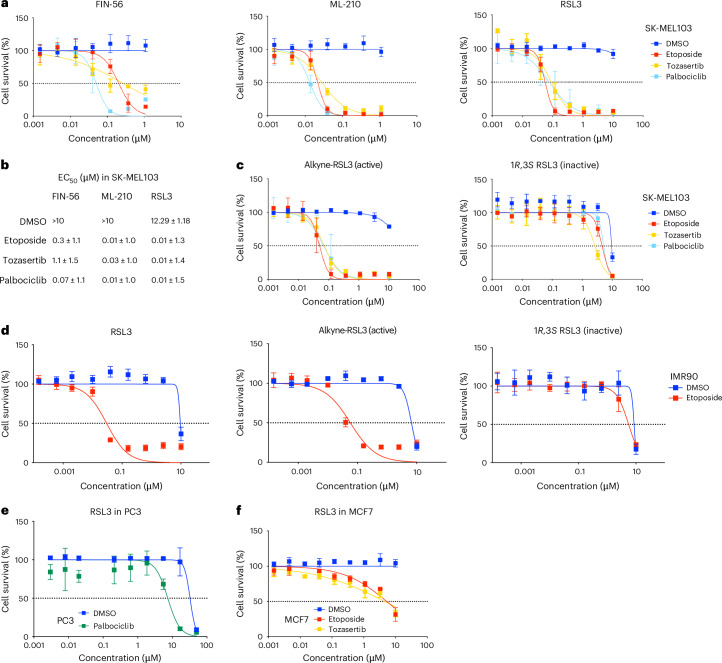
GPX4 inhibitors are senolytic. **a**, Dose–response curves of FIN-56, ML-210 and RSL3, three available GPX4 inhibitors in SK-MEL-103 cells. Data represent mean ± s.d. (*n* = 3 for FIN-56 and ML-210; *n* = 5 for RSL3 biological replicates). **b**, Table summarizing EC_50_ (μM) of the three GPX4 inhibitors in SK-MEL-103 cells. EC_50_ is obtained by calculating the geometric mean of the single replicate EC_50._ The standard error is calculated on the log-transformed data, back-transformed to the original scale. **c**, Dose–response curves of alkyne-RSL3 and the inactive isomers 1*R*, 3*S* RSL3 in SK-MEL-103 cells. Data represent mean ± s.d. (*n* = 3 biological replicates) **d**, Dose–response curves of RSL3, alkyne-RSL3 and 1*R*,3*S* RSL3 in IMR90 cells. Data represent mean ± s.d. (*n* = 3 biological replicates) **e**,**f**, Dose–response curves of RSL3 in PC3 (**e**) and MCF7 (**f**) cells. Data represent mean ± s.d. (*n* = 3 biological replicates). Source data

Previous works have suggested that GPX4 inhibitors can be senolytic^40,41^. To confirm that GPX4 is a senolytic target, we took advantage of three widely used GPX4 inhibitors: FIN-56, ML-210 and RSL3^42^. The three GPX4 inhibitors displayed senolytic activity in SK-MEL-103 cells in which senescence had been induced by either etoposide, tozasertib or palbociclib treatment (Fig. 4a). In particular, RSL3, a potent covalent GPX4 inhibitor, showed senolytic activity at concentrations more than 1,000 times lower than those needed to kill non-senescent control cells (Fig. 4b). Alk-RSL3, an alkynylated RSL3 derivative, is also senolytic (Fig. 4c, left). RSL3 is a chloroacetamide-containing covalent ligand with two stereocenters, with only one of four stereoisomers displaying highly potent GPX4 inhibition^39^. An inactive stereoisomer, (1*R*,3*S*) RSL3, showed no measurable senolytic activity (Fig. 4c, right). Similarly, RSL3 or Alk-RSL3, but not the (1*R*,3*S*) RSL3 inactive enantiomer, selectively killed IMR90 cells undergoing etoposide-induced senescence (Fig. 4d). Moreover, RSL3 also behaved as a senolytic, selectively killing PC3 cells undergoing palbociclib-induced senescence (Fig. 4e) and MCF7 cells undergoing etoposide- and tozasertib-induced senescence (Fig. 4f). The above results show that GPX4 inhibitors, such as RSL3, are senolytic.

### Senescent cells are primed for ferroptosis

**Fig. 5:**
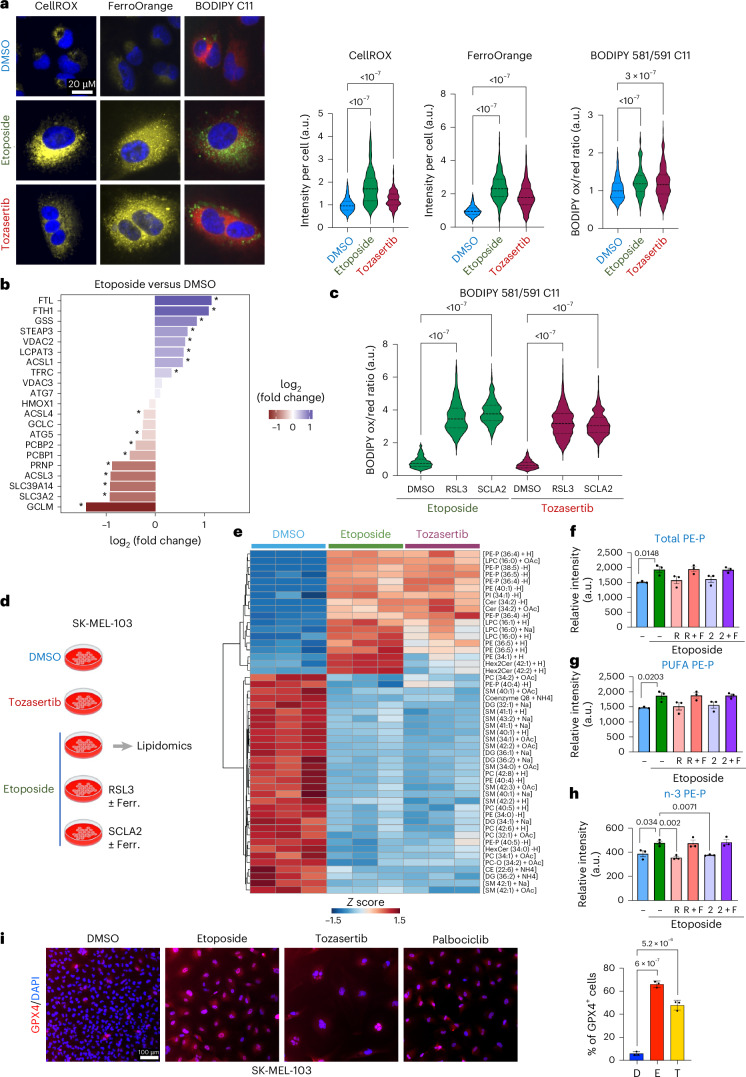
Senescent cells are primed for ferroptosis. **a**, SK-MEL-103 cells were induced to senesce for 6 days (Supplementary Table 8) and stained with either CellROX, FerroOrange or BODIPY 581/591 C11 to quantify ROS, intracellular Fe^2+^ or lipid peroxidation. Left, representative images. Scale bar, 20 μm. Right, mean fluorescence intensity per cell in one representative experiment out of three. CellROX *n* > 180 cells per condition; FerroOrange *n* > 85 cells per condition; BODIPY 581/591 C11 *n* > 149 cells per condition. Statistical test used: ordinary one-way ANOVA (Dunnett’s multiple comparisons test). **b**, Proteins annotated in the ‘Ferroptosis’ Kyoto Encyclopedia of Genes and Genomes (KEGG) pathway (hsa04216) and their associated differential abundance in SK-MEL-103 cells treated with DMSO or etoposide. *X*-axis denotes log_2_(fold change) of senescent versus non-senescent cells. Asterisks denote statistically significant differential abundance (adjusted *P* < 0.05, Welch’s *t*-test (two-tailed), Benjamini–Hochberg multiple testing correction. *n* = 4 biological replicates). **c**, SK-MEL-103 were treated for 2 h with RSL3 (1 μM) or SCLA2 (7 μM). Cells were then stained with BODIPY 581/591 C11. Mean fluorescence intensity per cell; *n* > 2,000 cells per condition. Statistical test used: one-way ANOVA (Dunnett’s multiple comparisons test). **d**, Schematic of lipidomic experiment. Lipid profiles were analysed by LC–MS (*n* = 3 biological replicates). Data are presented in Supplementary Table 5. Ferr., ferroptosis. **e**, Heat-map analysis of annotated lipid features showing the top 50 ones (on the basis of ANOVA). LPC, lysophosphatidylcholine; Cer, ceramide; Hex2Cer, dihexosylceramide; PC, phosphatidylcholine; DG, diacylglycerol; PI, phosphatidylinositol; HexCer, hexosylceramide; PC-O, ether-linked phosphatidylcholine (alkyl-PC). **f**–**h**, Relative abundance of PE-P (**f**), PUFA-containing PE-P (**g**) and n-3-containing PE-P lipids (**h**). Data (mean ± s.e.m.) were analysed by one-way ANOVA with Dunnett’s post-test: versus etoposide; or Student’s *t*-test: DMSO versus etoposide. PE-P, ether-linked phosphatidylethanolamine; PUFA PE-P, polyunsaturated ether-linked phosphatidylethanolamine; PE-P with n-3 or omega-3 fatty acids (20:5 and 22:6) were the ones that reached significance with the treatment. Data represent mean ± s.e.m. (*n* = 3 biological replicates). Statistical test used: unpaired *t*-test (two-tailed) for **f** to **g** and **h** (DMSO versus etoposide); one-way ANOVA (Dunnett’s multiple comparisons test) for **h**. **i**, Representative images of GPX4 expression (left) and quantification (right) in SK-MEL-103. Scale bar, 100 μm. Data represent mean ± s.d. (*n* = 3 biological replicates). Statistical test used: one-way ANOVA (Dunnett’s multiple comparisons test). Schematic in **d** created in BioRender; D’Ambrosio, M. https://biorender.com/dui3y7w (2025). Source data

**Extended Data Fig. 6:**
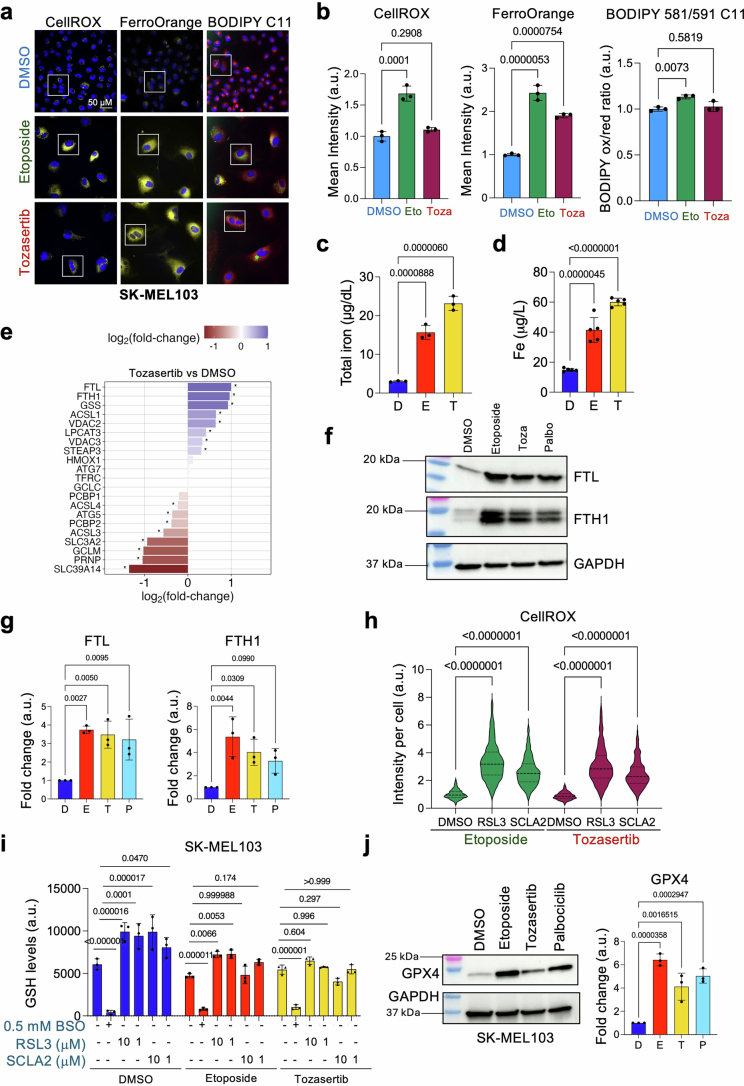
SK-MEL103 senescent cells are primed for ferroptosis. **a**, Left panel, representative images at lower magnification of Fig. 5a. Scale bar, 50 μm. **b**, Mean fluorescent intensity per cell. Mean ± SD (n = 3 biological replicates). One-way ANOVA (Dunnett’s multiple comparisons test). **c**, Total iron (mg/dL). Mean ± SD (n = 3 biological replicates). One-way ANOVA (Dunnett’s multiple comparisons test). **d**, Total cellular iron (mg/L) measured by mass spectrometry. Mean ± SD (n = 5 biological replicates). One-way ANOVA (Dunnett’s multiple comparisons test). **e**, Proteins annotated in the ‘Ferroptosis’ KEGG pathway (hsa04216) and differential abundance in SKMEL-103 cells treated with DMSO or Tozasertib. Asterisks denote statistically significant differential abundance (adjusted P < 0.05, Welch’s t-test (two-tailed), Benjamini-Hochberg multiple testing correction. n = 4 biological replicates). **f**, Western blot in SK-MEL103. GAPDH is a loading control. **g**, Quantification of FTL and FTH1. Mean ± SD (n = 3 biological replicates). One-way ANOVA (Dunnett’s multiple comparisons test). **h**, Mean fluorescent intensity per cell of CellROX^TM^ in senescent SK-MEL103 treated 2 hours with RSL3 (1mM) or SCLA2 (7 mM) (n>2000 cells per condition). One-way ANOVA (Dunnett’s multiple comparisons test). **i**, Intracellular glutathione levels in SK-MEL103 treated with BSO overnight or 4 h with RSL3 or SCLA2. Mean ± SD (n = 3 biological replicates). Two-way ANOVA (Tukey’s multiple comparisons test). **j**, Western blot images and quantification of GPX4 in SK-MEL103 cells. Mean ± SD (n =3 biological replicates). One-way ANOVA (Dunnett’s multiple comparisons test). GAPDH is a loading control. Source numerical data and unprocessed blots are available as source data. Source data

Ferroptosis is an iron-dependent form of cell death promoted by the accumulation of oxidized lipid membranes. Ferroptosis is characterized by increased levels of reactive oxygen species (ROS) and intracellular accumulation of labile ferrous ions (Fe^2+^) that contribute to unrestrained lipid oxidation^43–47^. To understand why senescent cells are more prone than normal counterparts to undergo ferroptosis on GPX4 inhibition, we used fluorescence probes to analyse ROS, levels of Fe^2+^ and lipid oxidation in senescent cells. SK-MEL-103 displayed higher levels of ROS, accumulated more intracellular Fe^2+^ and showed a moderate increase in levels of lipid oxidation (Fig. 5a and Extended Data Fig. 6a,b), all of which are hallmarks of ferroptosis. The increased accumulation of iron in senescent cells was confirmed by MS and using a colourimetric assay (Extended Data Fig. 6c,d). Previously, it has been reported that senescent cells accumulate iron, and this might correlate with an elevated expression of the transferrin receptor (TFRC) and the light and heavy chains of ferritin (FTL and FTH1)^48–50^. Proteomic analysis confirmed upregulation of TFRC, FTL and FTH1 in SK-MEL-103 cells undergoing etoposide-induced senescence (Fig. 5b). Similar induction of FTL and FTH1, but not TFRC, was observed in tozasertib-induced senescence (Extended Data Fig. 6e). The induction of FTL and FTH1 was confirmed by western blot (Extended Data Fig. 6f,g).

Treatment of SK-MEL-103 cells undergoing ether etoposide- or tozasertib-induced senescence with RSL3- or SCLA2-enhanced lipid oxidation (Fig. 5c and Supplementary Fig. 6a) and exacerbated ROS levels (Extended Data Fig. 6h and Supplementary Fig. 6b), consistent with induction of ferroptosis. The effect was due to on-target inhibition of GPX4 rather than reduction of GSH levels, as, contrary to the marked reduction in GSH observed with L-buthionine-sulfoximine (BSO), we observed only a small reduction with SCLA2 at 10 μM and no change at 1 μM (Extended Data Fig. 6i).

As lipid peroxidation is a defining characteristic of ferroptosis, we carried out untargeted lipidomics analysis in SK-MEL-103 cells undergoing etoposide or tozasertib-induced senescence (Fig. 5d and Supplementary Table 5). Unsupervised hierarchical clustering unveiled several lipid species that had increased abundance in both types of senescence, including multiple ether-linked phosphatidylethanolamines (PE) (Fig. 5e). Ether lipids promote susceptibility to ferroptosis, particularly when containing a polyunsaturated fatty acyl (PUFA) chain^51,52^. We treated senescent cells with the GPX4 inhibitor RSL3 or SCLA2 to assess their effect on levels of total ether PE (Fig. 5f) and PUFA-containing ether PE (Fig. 5g). Interestingly, we observed decreased levels of these lipids on treatment with RSL3 or SCLA2. This trend was prevented when cells were pretreated with ferrostatin 1, particularly for ether PEs containing n-3 PUFA (Fig. 5h), suggesting that the observed drop was due to lipid oxidation (Fig. 5f–h).

**Extended Data Fig. 7:**
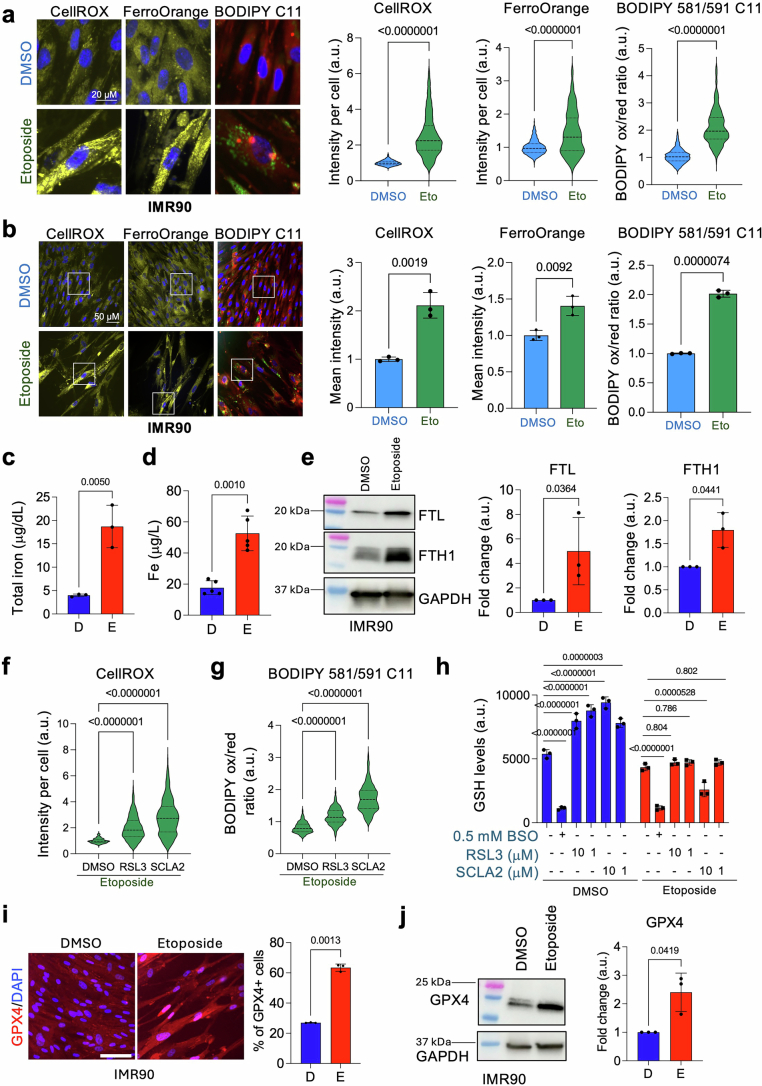
IMR90 cells undergoing etoposide-induced senescence are primed for ferroptosis. **a**, Left, representative images of CellROX^TM^, FerroOrange, or BODIPY™ 581/591 C11 (scale bar, 20 μm). Right, mean fluorescent intensity per cell. CellROX^TM^ n>350, FerroOrange n>215, BODIPY™ C11 n>419 cells per condition. Unpaired t-test (two-tailed). **b**, Left, lower magnification of the images in panel **a** (scale bar, 50 μm). Right, mean fluorescent intensity per cell. Mean ± SD (n = 3 biological replicates). Unpaired t-test (two-tailed). **c** Total iron (mg/dL). Mean ± SD (n = 3 biological replicates). Unpaired t-test (two-tailed). **d**, Total cellular iron (mg/L) measured by mass spectrometry. Mean ± SD (n = 5 biological replicates). Unpaired t-test (Welch’s correction, two-tailed). **e**, Western blots in IMR90 cells. Mean ± SD (n =3 biological replicates). Paired t-test (two-tailed). GAPDH is a loading control. **f-g**, Mean fluorescent intensity per cell of CellROX^TM^ (**f**) and BODIPY™ 581/591 C11 (**g**) in senescent IMR90 cells treated for 2 hours with RSL3 (1 μM) or SCLA2 (7 μM) (n>1200 cells per condition). Unpaired t-test (two-tailed). **h**, Intracellular glutathione levels in IMR90 cells treated with BSO overnight or for 4 h with RSL3 or SCLA2. Mean ± SD (n = 3 biological replicates). Two-way ANOVA (Tukey’s multiple comparisons test). **i**, Representatives GPX4 IF images (scale bar 100 μm) and quantification in IMR90 cells. Mean ± SD (n =3 biological replicates). Unpaired t-test (two-tailed). **j**, Western blot in IMR90 cells. Mean ± SD (n =3 biological replicates). Paired t-test (two-tailed). GAPDH is a loading control. Source numerical data and unprocessed blots are available as source data. Source data

**Extended Data Fig. 8:**
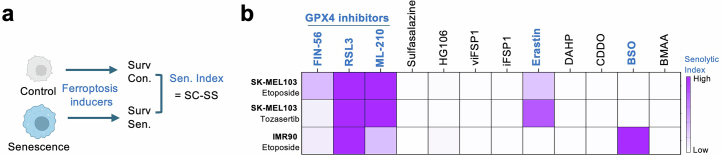
Senolytic activity of different ferroptosis inducers. **a**, Schematic of senolytic experiment performed with ferroptosis inducer in SK-MEL103 and IMR90 cells. **b**, Heatmap summarising the effect of different ferroptosis inducers on senescent SK-MEL103 and IMR90 cells. The senolytic index was calculated as the EC_50_ in non-senescent cells divided by the EC_50_ of senescent cells. Schematic in **a** created in BioRender; D’Ambrosio, M. https://biorender.com/3yd2zb9 (2025). Source data

As senescent cells seemed to be primed for ferroptosis, we reasoned that protective mechanisms against ferroptosis must be upregulated in senescent cells. Quantitative immunofluorescence (IF; Fig. 5i) and western blot analysis (Extended Data Fig. 6j) found elevated levels of GPX4 in senescent SK-MEL cells. Similar results, including elevated ROS, Fe^2+^ and GPX4 levels, were observed in IMR90 cells undergoing etoposide-induced senescence (Extended Data Fig. 7 and Supplementary Fig. 6c,d). To understand if other mechanisms might prevent ferroptosis on senescent cells, we tested a battery of ferroptosis inducers (Extended Data Fig. 8a). While erastin (an inhibitor of the cystine–glutamate antiporter system Xc) was senolytic in SK-MEL-103 cells and BSO (a sulfoximine derivative that reduces levels of glutathione) in IMR90 cells, only the three GPX4 inhibitors tested showed senolytic activity in all senescent models tested (Extended Data Fig. 8b). Overall, the above results show that senescent cells display high levels of ROS, increased Fe^2+^ and alterations in lipid metabolism that prime them for ferroptosis, a fate prevented by GPX4 upregulation.

### GPX4 inhibitors target premalignant senescent hepatocytes

**Fig. 6:**
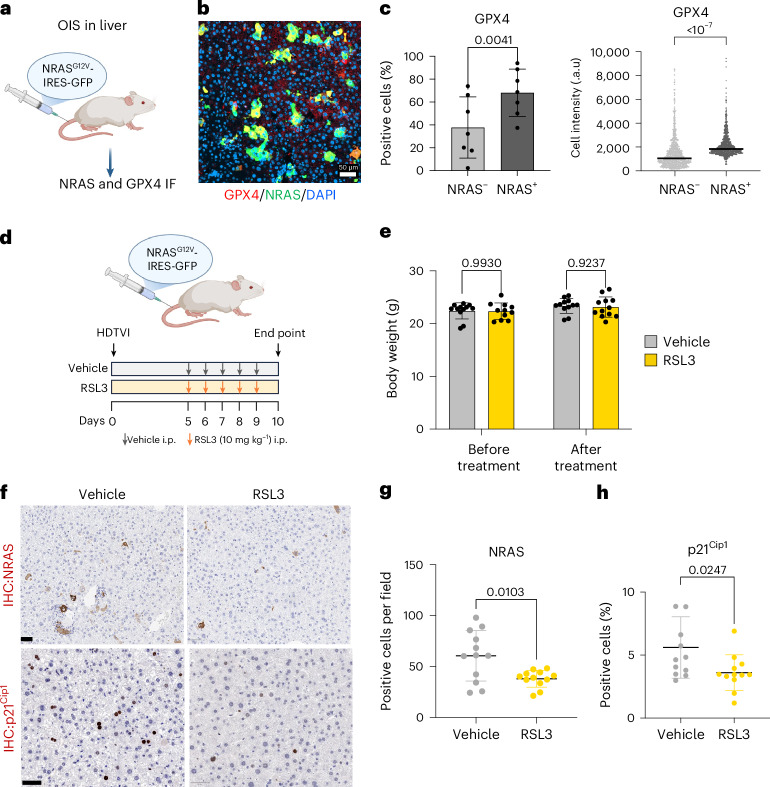
RSL3 targets premalignant senescent hepatocytes. **a**, Schematic representation of the OIS model in the liver. **b**, Representative IF images (one mouse/seven mice) of GPX4 and NRAS in liver samples from the HDTVI mouse model. Scale bar, 50 μm. **c**, Left: percentage of GPX4 positive cells in NRAS^+^ and NRAS^−^ cells. Data represent mean ± s.d. (*n* = 7 mice). Statistical test used: paired *t*-test (two-tailed). Right: GPX4 fluorescence intensity in NRAS^+^ cells and NRAS^−^ cells. Representative data from one mouse out of seven analysed (*n* = 1,000 NRAS^+^ cells and *n* = 1,000 NRAS^−^cells). Data represent mean ± s.d. Statistical test used: unpaired *t*-test with Welch’s correction (two-tailed). **d**, Experimental scheme. The NRAS^G12V^ transposon construct was delivered into mouse livers via HDTVI on day 0. Mice received daily i.p. injections of RSL3 (10mg kg^−1^) or vehicle for five consecutive days starting from day 5 (day 5 to day 9). Mice were killed 10 days after HDTVI. **e**, Mice body weight before and after treatment with RSL3 or vehicle. Data represent mean ± s.d. (*n* = 12 mice in each group). Statistical test used: two-way ANOVA (Šídák’s multiple comparisons test). **f**, Representative IHC staining of NRAS and p21^Cip1^ in mice livers (*n* = 12 mice). Scale bar, 50 μm. **g**, Quantification of NRAS positive cells. Data represent mean ± s.d. (*n* = 12 mice in each group). Statistical test used: unpaired *t*-test with Welch’s correction (two-tailed). **h**, Quantification of p21^Cip1^ staining in vehicle and RSL3-treated mice. Data represent mean ± s.d. (*n* = 12 mice in each group). Statistical test used: unpaired *t*-test with Welch’s correction (two-tailed). Schematics in **a** and **d** created in BioRender; D’Ambrosio, M. https://biorender.com/gcqqw41 (2025). Source data

Next, we investigated whether GPX4 was also upregulated in senescent cells in vivo. To this end, we analysed a model of cancer initiation in the liver in which OIS is induced in hepatocytes via transposon-induced expression of oncogenic NRAS (NRAS^G12V^)^53,54^ (Fig. 6a). Quantitative IF of liver sections showed higher expression of GPX4 in NRAS^+^ senescent cells (Fig. 6b,c).

To understand if treatment with a GPX4 inhibitor was able to eliminate senescent cells in vivo, we treated mice transduced with an NRAS^G12V^-expressing transposon with RSL3 (Fig. 6d). The treatment was well tolerated, as we did not observe a difference in weight between RSL3 and vehicle groups (Fig. 6e). Mice treated with RSL3 displayed a significant reduction in the number of both NRAS-positive cells, as assessed by reduced NRAS staining (Fig. 6f,g), and p21-positive cells (Fig. 6f,h), suggesting that treatment with RSL3 was targeting a population of NRAS-positive, p21-positive senescent hepatocytes^54^. Together, these results show that RSL3 treatment is well tolerated and can eliminate senescent cells in vivo.

### GPX4 inhibitors are senolytic in xenograft models of melanoma and prostate cancer

**Fig. 7:**
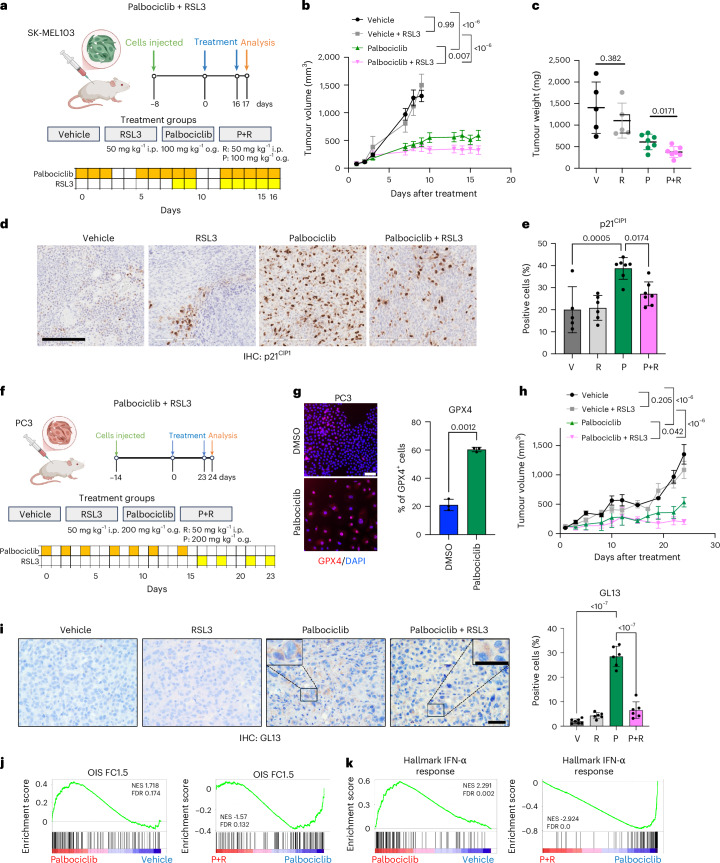
GPX4 inhibitors can be used as senolytics in one-two-punch anticancer therapies. **a**, Schematic of the SK-MEL-103 xenograft experiment. **b**, Graph showing the size (in mm^3^) of the tumours. The *P* values were determined by two-way ANOVA followed by Tukey’s multiple comparison test at the end point, separately for every single treatment. Data are represented as mean ± s.e.m. **c**, Tumour weight (mg) of mice treated with vehicle (V), RSL3 (R), palbociclib (P) and palbociclib + RSL3 (P+R). Measurements at the end point. Statistical test used: unpaired *t*-test (two-tailed) comparing V versus R groups and P versus P+R groups. Data are represented as mean ± s.d. **d**, Representative images of p21^Cip1^ IHC staining in SK-MEL-103 tumours. Scale bar, 200 μm. **e**, Quantification of p21^Cip1^ IHC staining in SK-MEL-103 tumours. Data represent mean ± s.d. Statistical test used: one-way ANOVA (Tukey’s multiple comparisons test). For panels **a**–**e**, *n* = 5 mice for V and R, *n* = 7 mice for P and P+R. **f**, Schematic of the PC3 xenograft experiment. **g**, Representative IF images of GPX4 expression (left) and quantification (right) in non-senescent and senescent PC3 cells. Scale bar, 100 μm. Data represent mean ± s.d. (*n* = 3 biological replicates). Statistical test used: unpaired *t*-test with Welch’s correction (two-tailed). **h**, Graph showing the size (in mm^3^) of the xenograft tumours. The *P* values were determined by two-way ANOVA followed by Tukey’s multiple comparison test at the end point, separately for every single treatment. Data are represented as mean ± s.e.m. **i**, Representative images and quantification of GL13 IHC staining in PC3 tumours. Scale bar, 25 μm. Data represent mean ± s.d. Statistical test used: one-way ANOVA (Tukey’s multiple comparisons test). **j**, GSEA enrichment plots of OIS signature in vehicle versus palbociclib (left) and palbociclib versus palbociclib + RSL3 (right). **k**, GSEA enrichment plots of hallmark IFN-α response signature in vehicle versus palbociclib (left) and palbociclib versus palbociclib + RSL3 (right). For **f** to **k**, *n* = 7 mice for vehicle, *n* = 6 mice for vehicle + RSL3, palbociclib and palbociclib + RSL3. o.g., oral gavage. Schematic created in BioRender: **a**, D’Ambrosio, M. https://biorender.com/22mlqo5 (2025); **f**, D’Ambrosio, M. https://biorender.com/gfr0emj (2025). Source data

**Extended Data Fig. 9:**
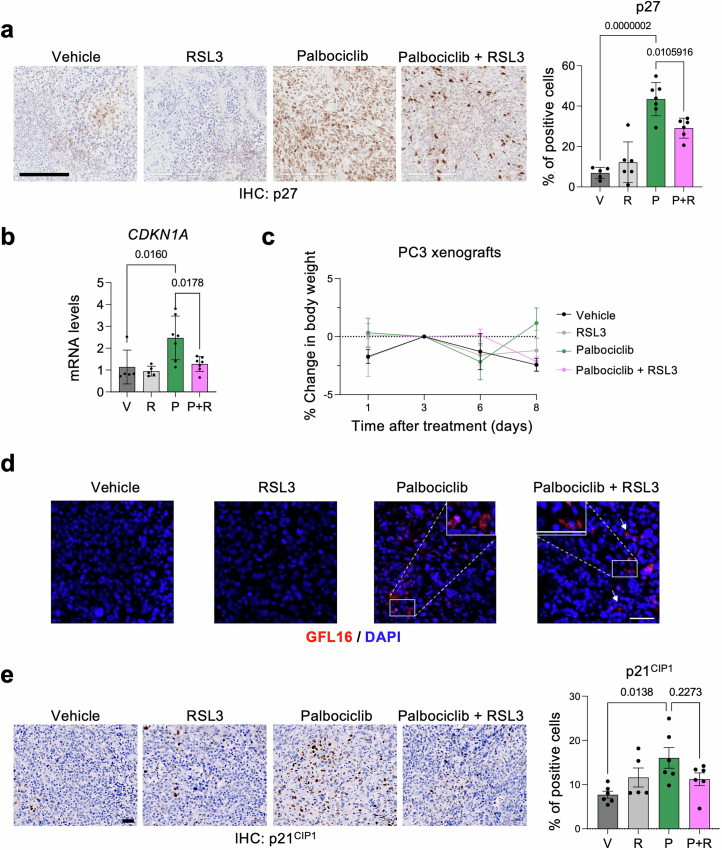
GPX4 inhibitors can be used as senolytics in one-two-punch anticancer therapies. **a**, Left panel, representative images of p27 immunohistochemical staining in SK-MEL103 tumours. Scale bar, 200 μm. Right panel, Quantification of p27 IHC staining in SK-MEL103 tumours. Data represent mean ± SD. Statistical test used: one-way ANOVA (Tukey’s multiple comparisons test). **b**, Bar graph showing *CDKN1A* mRNA levels in SK-MEL103 tumours. Data represent mean ± SD. Statistical test used: one-way ANOVA (Tukey’s multiple comparisons test). For panel **a-b**, n = 5 mice for V and R, n = 7 mice for P and P+R. **c**, Change in the PC3-injected mice’s body weight (%) during drug administration. n = 7 mice for vehicle and Palbociclib+ RSL3, n = 5 mice for RSL-3 and n = 6 mice for Palbociclib. Data are represented as mean ± SEM. **d**, Representative IF images of GFL16 in PC3 tumours. Scale bar, 25 μm in both figures and mags (n = 7 mice for V, n = 6 mice for R, P and P+R). **e**, Left panel, representative images of p21^Cip1^ IHC staining in PC3 tumours. Scale bar. 50 μm. Right panel, quantification of p21^Cip1^ IHC staining in PC3 tumours. Data represent mean ± SEM. Statistical test used: one-way ANOVA (Tukey’s multiple comparisons test). For panel **e**, n = 6 mice for V, P and P+R and n = 5mice for R. Source data

To assess whether GPX4 inhibitors could be used in combination with anticancer therapies in one-two punch approaches^5,6^, we first examined a xenograft model of melanoma in which SK-MEL-103 cells were treated with palbociclib and RSL3, as indicated (Fig. 7a). We injected SK-MEL-103 cells subcutaneously (s.c.), and when tumours became established, we started treatments. After 2 weeks of treatment, we observed a significant reduction in tumour volume in the palbociclib + RSL3-treated mice when compared with all other groups (Fig. 7b,c). Analysis of tumour samples showed an upregulation of p21^CIP1^ and p27 on palbociclib treatment and a significant reduction when compared with the palbociclib + RSL3-treated mice (Fig. 7d,e and Extended Data Fig. 9a,b).

To extend these results and understand whether RSL3 eliminated senescent tumour cells, we used a xenograft model of PC3 prostate cancer cells (Fig. 7f). Importantly, palbociclib-induced senescence in PC3 cells upregulated GPX4 (Fig. 7g). All treatments were well tolerated (Extended Data Fig. 9c) and we observed a significant reduction in the tumour volume in palbociclib + RSL3-treated mice when compared with all other groups (Fig. 7h). SenTraGor stains lipofuscin-positive cells and is used as a biomarker of senescence suitable for use in formalin-fixed material^55,56^. We observed an increase in SenTraGor-positive cells (as assessed by GL13 or GFL16 staining) in palbociclib-treated tumours. There was a reduction in SenTraGor-positive cells on treatment with palbociclib + RSL3 (Fig. 7i and Extended Data Fig. 9d). Moreover, a human-specific p21^CIP1^ antibody (that stained xenografted tumour cells but not mouse-derived cells) confirmed a significant increase in p21-positive tumour cells on palbociclib treatment and a non-significant reduction in the palbociclib + RSL3 group (Extended Data Fig. 9e). We also conducted transcriptome analysis of the tumours. By performing gene set enrichment analysis (GSEA) limited to human-derived reads, we confirmed the upregulation of senescence and IFN-α response signatures on palbociclib treatment and their downregulation when compared with palbociclib + RSL3-treated mice (Fig. 7j,k). The above results show that GPX4 inhibitors can be used in the context of one-two punch anticancer therapies.

### One-two-punch therapies with RSL3 improve the survival of a mouse model of ovarian cancer

**Fig. 8:**
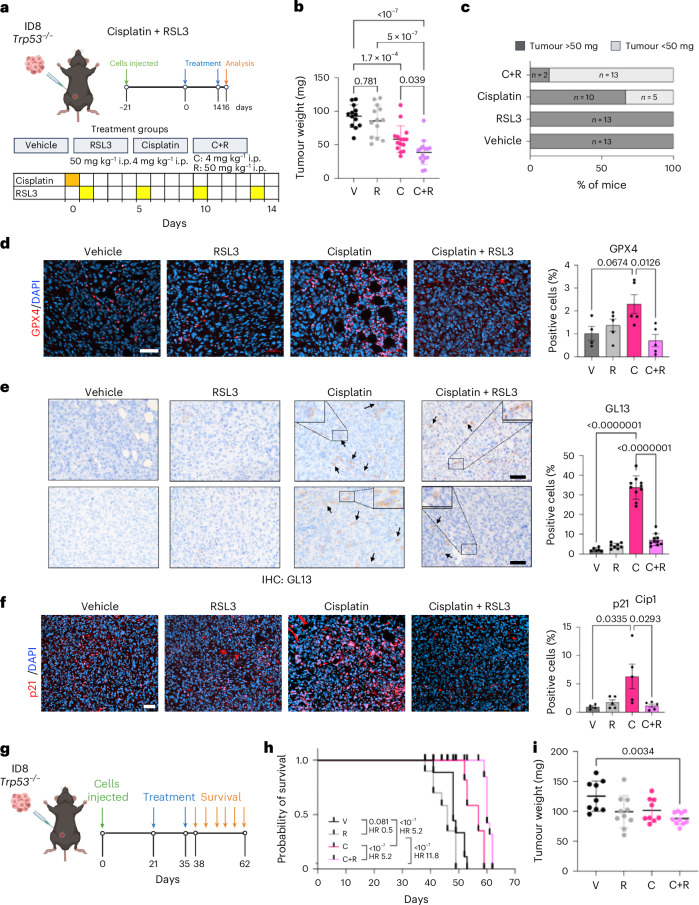
GPX4 inhibitors are senolytics and improve survival in an ovarian cancer model. **a**, Schematic of the ID8 *Trp53*^−/−^ xenograft experiment. **b**, Tumour weight (mg) of mice treated with vehicle (V), RSL3 (R), cisplatin (C) and cisplatin + RSL3 (C+R). Data represent mean ± s.d. Statistical test used: one-way ANOVA (Tukey’s multiple comparisons test). **c**, Percentage of mice with tumour weight below 50 mg in from **b**. **d**, Representative images and quantification of GPX4 IF staining in omental tumours. Scale bar, 50 μm. Data represent mean ± s.e.m. Statistical test used: one-way ANOVA (Tukey’s multiple comparisons test). **e**, Representative images and quantification of GL13 IHC staining in ID8 *Trp53*^−/−^ omental tumours. Scale bar, 25 μm in both figures and inset. Data represent mean ± s.d. Statistical test used: one-way ANOVA (Tukey’s multiple comparisons test). **f**, Representative images and quantification of p21^Cip1^ IF staining in omental tumours. Data represent mean ± s.e.m. Statistical test used: one-way ANOVA (Tukey’s multiple comparisons test). For **a** to **c**, *n* = 13 mice for the V and R groups, *n* = 15 mice for the C and C+R groups. For **d** and **f**, *n* = 4 mice for the V group and *n* = 5 mice for the R, C and C+R groups. For **e**, *n* = 6 mice for the V group, *n* = 8 mice for the R group and *n* = 10 mice for the C and C+R groups. **g**, Survival xenograft ID8 *Trp53*^−/−^ experiment. **h**, Kaplan–Meier survival curves for mice treated with V, R, C and C+R. Mice were injected with tumour cells and treated as in **a**. Mice were checked and weighed daily and killed when they showed any sign of distress. Statistical test used: log-rank (Mantel–Cox test). **i**, Tumour weight (mg) of mice treated with V, R, C and C+R. Tumour weight was taken at killing. Data represent mean ± s.d. Statistical test used: one-way ANOVA (Tukey’s multiple comparisons test). For **h** and **i**, *n* = 9 mice for V and C groups and *n* = 10 mice for R and C+R groups. Schematic created in BioRender: **a**, D’Ambrosio, M. https://biorender.com/l12nbuo (2025); **g**, D’Ambrosio, M. https://biorender.com/ag57v7e (2025). Source data

**Extended Data Fig. 10:**
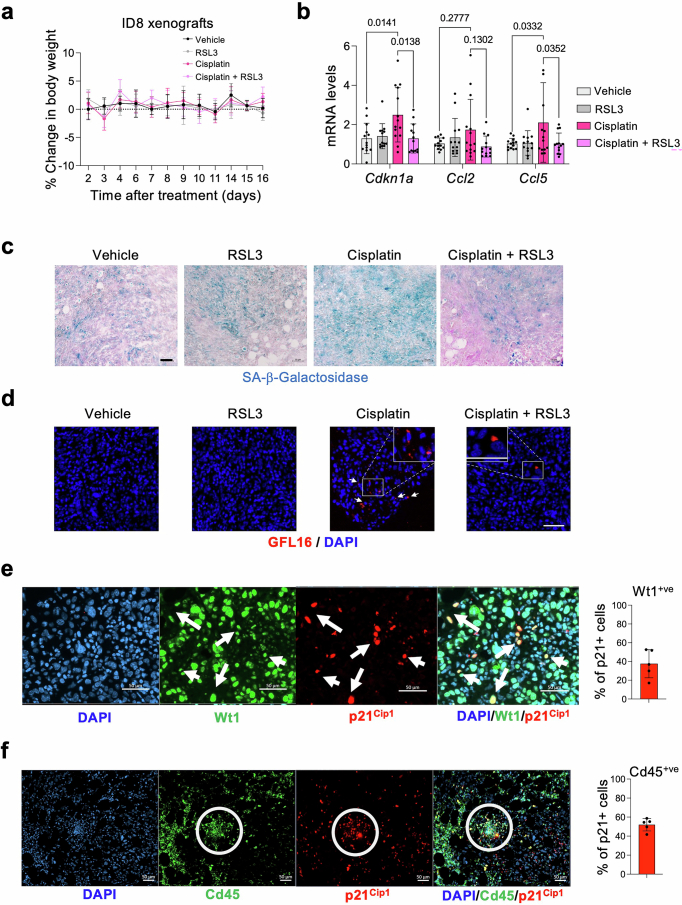
GPX4 inhibitors can be used as senolytics in one-two-punch anticancer therapies in an ovarian cancer model. **a**, Change in the ID8-injected mice’s body weight (%) during drug administration. n = 14 mice for vehicle group and n = 15 mice for RSL3, Cisplatin and Cisplatin+RSL3 groups. Data are represented as mean ± SD. **b**, *Cdkn1a*, *Ccl2* and *Ccl5* mRNA levels in ID8 *Trp53*^*−/−*^ omental tumours. Data represent mean ± SD. Statistical test used: two-way ANOVA (Tukey’s multiple comparisons test). For panel **b**, n = 13 mice for V, R and C+R groups, n = 14 mice for R, C and C+R. **c**, Representative images of Senescence-associated b-galactosidase (SA-b-gal) staining in omental tumours. Scale bar, 50 μm (n=4 mice for V, n=5 mice for R, C and C+R). **d**, Representatives IF images of GFL16 in ID8 *Trp53*^*−/−*^ omental tumours. Scale bar, 25 μm in both figures and mags (n=6 mice for V, n=8 mice for R and n=10 mice for C and C+R). **e**, Representative images (left panel) and quantification (right panel) of Wt1/p21 co-staining in ID8 *Trp53*^*−/−*^ omental tumours treated with cisplatin. Scale bar, 50 μm. Data represent mean ± SD (n = 5 mice). **f**, Representative images (left panel) and quantification (right panel) of CD45/p21 co-staining in ID8 *Trp53*^*−/−*^ omental tumours treated with cisplatin. Scale bar, 50 μm. Data represent mean ± SD (n = 5 mice). Source data

To investigate the therapeutic potential of RSL3 as a senolytic in an immunocompetent setting, we used the previously described ID8 *Trp53*^−/−^ ovarian cancer model^57^. We injected ID8 *Trp53*^−/−^ cells intraperitoneally (i.p.) and, once tumours became established, divided mice into four cohorts and examined the tumours 16 days after treatment (Fig. 8a). All treatments were well tolerated, as we did not observe any significant weight loss (Extended Data Fig. 10a). While treatment with RSL3 alone did not decrease tumour size, treatment with cisplatin reduced mean tumour size (Fig. 8b). The combination of cisplatin and RSL3 also caused a significant reduction in mean tumour size when compared with all other groups (Fig. 8b). Moreover, a higher percentage of tumours treated with the combination of cisplatin and RSL3 were smaller in size, when compared with those in the vehicle or the cisplatin-treated groups (Fig. 8c).

Transcripts encoding for *Cdkn1a* and SASP components, such as *Ccl2* and *Ccl5*, were upregulated in cisplatin-treated mice and significantly reduced in mice undergoing combination treatment (Extended Data Fig. 10b). Cisplatin treatment also upregulated GPX4 in tumour sections. Interestingly, the levels of GPX4 were reduced in the cohort treated with cisplatin + RSL3 (Fig. 8d). To assess the effect of cisplatin + RSL3 in eliminating senescent cells, we stained sections for SA-β-galactosidase or took advantage of SenTraGor. In both instances, we observed an increase in senescence on cisplatin treatment and a reduction in the cisplatin + RSL3 sections (Fig. 8e and Extended Data Fig. 10c,d). Quantification of GL13-stained sections confirmed these observations (Fig. 8e). Analysis of p21-positive cells was consistent with increased senescence on cisplatin treatment and a significant reduction on combination treatment (Fig. 8f). As the implanted tumour cells lack p53 expression (ID8 *Trp53*^−/−^), and p21 is a p53-dependent gene, we wanted to confirm that (a subset of) the p21-positive cells were tumour derived. To this extent, we costained p21 with either an epithelial tumour antigen (Wt1) or an immune marker (Cd45). While part of the p21-positive cells had an immune origin, around 40% of the p21-positive cells were tumour derived (Extended Data Fig. 10e,f).

Finally, we assessed whether combined treatment with cisplatin + RSL3 improved the survival in the ID8 *Trp53*^−/−^ model of ovarian cancer (Fig. 8g). Mice were treated as described earlier, and survival was assessed daily. Cisplatin treatment resulted in a significant survival increase, as previously described^57^ (Fig. 8h). Interestingly, mice treated with cisplatin + RSL3 displayed a significant extension of survival when compared with any of the other groups (Fig. 8h). Moreover, we also observed a significant reduction in the weight of the cisplatin + RSL3 tumours when compared with the vehicle-treated group at the end point (Fig. 8i). The above results show that GPX4 is upregulated by anticancer therapies in vivo, and GPX4 inhibitors in combination with cisplatin reduced tumour size and increased survival in a mouse model of ovarian cancer.

## Discussion

To identify emerging senolytics, we screened a library of electrophilic compounds, which can target proteins through covalent bond formation. We identified 38 electrophiles with senolytic activity. Among these, there were electrophiles with warheads that target either cysteines (for example, acrylamides or chloroacetamides) or other nucleophilic residues such as lysine (for example, aldehydes or sulfonyl fluorides).

We concentrated our studies on four chloroacetamides displaying senolytic activity in different models of senescence. GPX4 was a target of three of the four senolytic chloroacetamides. GPX4 is a glutathione peroxidase that prevents ferroptosis by reducing lipid peroxidation^38^. We observed senolysis by ferroptosis when senescent cells were treated with one of these three inhibitors. By extending the analysis to 15 electrophilic compounds, we noticed that a subset (4/7 tested) of senolytic chloroacetamides induced ferroptosis. Interestingly, one of our senolytic chloroacetamides, SCLA1 (CLA01-P20, cat no. Z56926666), was identified in a patent search for GPX4 inhibitors, further validating our target identification^58^.

As many of the signals that induce senescence can also cause apoptosis, senescent cells rely on protective mechanisms to survive. For example, senescent cells upregulate BCL2 family proteins such as BCL-XL, BCL-W^24^ or MCL-1^25^ to prevent intrinsic apoptosis. Similarly, senescent cells induce DR5 and its ligand TRAIL, being primed to die via extrinsic apoptosis, a fate prevented by cFLIP induction^26^. These antiapoptotic mechanisms constitute vulnerabilities and, consequently, targeting them with either BCL2 family inhibitors^24,25,34^ or via suppression of cFLIP^26^ kills senescent cells. Consistent with their increased oxidative stress and altered iron homeostasis^50^, senescent cells are also primed for ferroptosis, upregulating GPX4 to avoid cell death. Consequently, GPX4 inhibitors, including RSL3^42^ and the chloroacetamides described here display senolytic activity.

Despite the protective effects associated with GPX4 expression, mice heterozygote for GPX4 (*Gpx4*±) live longer^59^. This increased lifespan correlated with a decreased incidence of lymphomas and reduced glomerulonephritis. While increased sensitivity to apoptosis was detected, whether there was any effect on senescence was not analysed but it is an interesting possibility. GPX4 inhibitors have been tested as anticancer therapies with limited success, as they are associated with toxicities, including bone defects and kidney damage^60^. In this regard, their use on one-two punch approaches (in which senescence inducers are combined with senolytics) could enhance their effectiveness, as senescent cells are more sensitive to GPX4 inhibition. Using three cancer models, we show that induction of senescence by either palbociclib or cisplatin makes tumours sensitive to GPX4 inhibitors at concentrations at which toxicities are spared. While we used several markers (including SA-β-gal, lipofuscin, cyclin-dependent kinase inhibitors, SASP components and transcriptional senescence signatures) to characterize senescence in these models, we have to acknowledge the limitations of our study given the inherent heterogeneity of senescent cells. Moreover, while by using costaining with tumour markers or detecting tumour-specific proteins or transcripts, we showed that we are targeting senescent tumour cells; it is important to note that part of the effects noted might be due to targeting stromal senescent cells.

Therapy-resistant cancer cells are also more sensitive to GPX4^61,62^. These persister cancer cells were characterized by a high-mesenchymal cell state^62^. In general, a higher iron load in cancer cells, cancer stem cells or persister cells confers vulnerability to ferroptosis^45–47^. While our results are consistent with a senolytic effect of GPX4 inhibitors, the effects observed in some of the models, although significant, were modest, and we acknowledge that further investigation is needed to assess a potential translation to the clinic. Overall, GPX4 inhibitors could have a combined effect on eliminating senescent cells in the tumour and tumour microenvironment and the subset of highly plastic, mesenchymal-like cells that promote therapy resistance.

In summary, our results suggest that electrophilic compounds can expand the range of available senolytic targets. Here, we identify GPX4 as a vulnerability of senescent cells. Senescent cells are primed for ferroptosis and upregulate GPX4 as a protective mechanism. Consequently, GPX4 inhibitors behave as a alternative class of senolytic drugs that kill senescent cells by ferroptosis and can be used to treat senescence-associated diseases such as cancer.

## Methods

### Ethics

This research complied with all relevant ethical regulations and was approved and overseen by the following ethics review boards. Experiments performed in the ovarian cancer model and the liver cancer initiation model were approved by the Animal Welfare and Ethics Review Body (AWERB) at Imperial College London. The experiments conformed to UK Home Office regulations under the Animals (Scientific Procedures) Act 1986, including Amendment Regulations 2012, and adhered to the Animal Research: Reporting of in Vivo Experiments (ARRIVE) guidelines. Liver cancer initiation experiments to assess GPX4 levels were performed under project licence number PPL 70/09080. Ovarian cancer experiments were performed under the project licence number PP1321516. Prostate cancer and melanoma experiments in mice were conducted in the BIOS+ animal facility in Bellinzona under specific pathogen-free conditions, with approval from project licences 35293, 36369 and 37480. The hydrodynamic tail vein injection experiment with RSL3 was carried out according to German law and with the approval of the Regierungspräsidium Karlsruhe (approval no. G-213/20). In all the animal facilities, mice were maintained under a 12-h light–12-h dark cycle.

### Antibodies

The primary antibodies used for IF and western blotting are presented in Supplementary Table 6.

### Drugs

The compounds used in this study are presented in Supplementary Table 7.

### Alkynylated probes derived from senolytic chloroacetamides

A detailed description of the synthesis of alkynylated probes derived from senolytic chloroacetamides and the NMR spectra of the synthesized probes is provided in Supplementary Note 1.

### Cell lines

IMR90 (human, American Type Culture Collection (ATCC) CCL-186), SK-MEL-103 (ATCC, HTB-69), A549 (ATCC, CCL-185), MCF7 (ATCC, HTB-22) and PC3 (ATCC, CRL-1435) cells were obtained from the ATCC. ID8 *Trp53*^−/−^ ovarian cancer cells (derived from C57BL/6 mouse) have been described before^57^. IMR90 ER:RAS cells were generated by retroviral infection of IMR90 cells and have been described elsewhere^33^. IMR90, SK-MEL-103, A549, MCF7 and IMR90 ER:RAS were cultured in Dulbecco’s modified Eagle’s medium (DMEM; Gibco, cat. no. 41965120) supplemented with 10% fetal bovine serum (FBS; Sigma, cat. no. F7524) and 1% antibiotic–antimycotic solution (Gibco, cat. no. 15240062). ID8 *Trp53*^−/−^ was cultured in DMEM (high glucose, pyruvate; Gibco, cat. no. 11995-073) with 4% FBS (Sigma, cat. no. F7524), 1% Anti-Anti (Gibco, cat. no. 15240-062) and 1% Insulin-Transferrin-Selenium (Gibco, cat. no. 2506891). PC3 was cultured in Roswell Park Memorial Institute medium (Gibco, cat. no. 12004997) supplemented with 10% FBS and 1% antibiotic–antimycotic solution.

### Growth assays

For BrdU incorporation assays, the cells were incubated with 10 μM BrdU (Sigma, 858811) for 16–18 h and then fixed with 4% paraformaldehyde (PFA; Thermo Scientific, 033314.M1). BrdU incorporation was assessed by IF and high-content analysis microscopy. For crystal violet staining, the cells were seeded at a low density in six-well plates and fixed at the end of the treatment with 0.5% glutaraldehyde. The plates were then stained with 0.2% crystal violet.

### Cytochemical SA-β-galactosidase assay

Cells were seeded in six-well plates, fixed with 0.5% glutaraldehyde (Sigma, G5882) in PBS for 15 min, washed with 1 mM MgCl_2_/PBS (pH 6.0) and then incubated with X-Gal staining solution (1 mg ml^−1^ of X-Gal (Thermo Scientific, 2737087), 5 mM K_3_(Fe(CN)_6_) and 5 mM K_4_(Fe(CN)_6_) for 6–8 h at 37 °C. Bright-field images were captured using the DP20 digital camera attached to the Olympus CKX41 inverted light microscope. The percentage of SA-β-galactosidase-positive cells was estimated by counting at least 100 cells per replicate sample, facilitated by the ‘point picker’ tool of ImageJ software (National Institutes of Health).

Fluorescence SA-β-galactosidase assay was performed in live cells, using C_12_FDG (Abcam, ab273642). Cells were cultured in a 96-well plate for 6 or 7 days to establish senescence response, as previously described (Supplementary Table 8). Once the senescence response was established, cells were treated with 100 nM Bafilomycin (Sigma, B1793) for 1 h in the incubator, followed by the addition of 30 μM C_12_FDG for 1 h in the incubator. Cells were fixed with 4% PFA and nuclei were counterstained with 4,6-diamidino-2-phenylindole (DAPI), as described above. Image acquisition and analysis were performed as previously described.

### IF staining of cells

Cells were seeded in 96-well plates (Nunc MicroWell, 167008) and cultured, as required. At the desired time point, cells were fixed with 4% PFA for 45 min, permeabilized with 0.2% Triton X-100 (Sigma, 11332481001) diluted in phosphate-buffered saline (PBS) for 10 min and blocked with 1% bovine serum albumin (Sigma, cat. no. A2153) for 1 h at room temperature. Cells were then incubated with a primary antibody for 1–2 h at room temperature, followed by the corresponding fluorophore-conjugated secondary antibody (Alexa Fluor) for 45 min and DAPI staining (1 μg ml^−1^ DAPI; Sigma, D9542) for 15 min at room temperature. For BrdU staining, the primary BrdU antibody was added together with 0.5 U μl^−1^ DNAse (Sigma, D5025) and 1 mM MgCl_2_ (Sigma, M8266) in the blocking solution and incubated for 30 min at room temperature. A complete list of primary and secondary antibodies used is presented in Supplementary Table 6. Antibodies were diluted in a blocking solution. After every step, cells were washed with PBS three times. All the antibodies used are presented in Supplementary Table 6.

### Live intracellular fluorescence stainings

CellROX Orange Reagent, for oxidative stress detection (C10443, Invitrogen, Thermo Fisher Scientific), FerroOrange (F374, Dojindo), and BODIPY 581/591 C11 (D3861, Invitrogen, Thermo Fisher Scientific) were used according to the manufacturer’s instructions. In brief, plated cells were incubated for 30 min at 37 °C, 5% CO_2_ in the staining solution (HBSS containing 1 μg ml^−1^ Hoechst 33342 (62247, Invitrogen, Thermo Fisher Scientific) and either 5 μM CellROXTM, 1 μM FerroOrange or 1 μM BODIPY 581/591 C11). Images were acquired extemporaneously using the IN Cell 2500 Analyzer high-throughput microscope (GE Healthcare) with a 40× objective and nine fields at least per well. Staining quantification was performed using IN Carta v1.17.0412545 software (Molecular Devices).

### High-throughput microscopy and quantitative analysis

Images of wells from 96-well plates were acquired using the IN Cell Analyser 2500HS high-throughput microscope and plate reader (Cytiva). For images of only DAPI (cell count), images of wells were captured at a 10× objective. For all IF stainings, wells were imaged with a 20× objective except BrdU staining (imaged using a 10× objective). Fluorescence images of fluorophores were captured on the basis of the preset wavelength settings for ‘DAPI’ (for DAPI staining), ‘Red’ (for Alexa Fluor 594) and ‘Green’ (for Alexa Fluor 488) on the IN Cell microscope. High content analysis (HCA) of acquired images was performed using the IN Cell Investigator 2.7.3 software (GE Healthcare) or IN Carta v1.17.0412545 software (Molecular Devices). DAPI nuclear staining was used as a nuclear mask to quantify cells and allow the segmentation of cells on the basis of a Top-Hat method, according to the software guidelines. This method also allowed the detection of nuclear-localized antibody staining. Nuclear stainings were quantified by the average pixel intensity within the nuclear mask. Cytoplasmic staining was quantified after applying a collar of 6–9 μm around the DAPI mask. Cytoplasmic-localized staining was quantified either by the average pixel intensity or by measuring the coefficient of variance of pixel intensity within the cell collar. Detection of percentage positive cells was based on a threshold, typically set according to the average pixel intensity of an internal negative control or an unstained secondary antibody-only control sample, unless specified.

### Iron quantification by inductively coupled plasma–triple quadrupole mass spectrometer

SK-MEL-103 and IMR90 cells were cultured in 15-cm dishes, and senescence was induced for 6 days with etoposide and tozasertib (SK-MEL-103) or etoposide (IMR90) treatment (density and drug concentration are indicated in Supplementary Table 8). At day 6, cells were washed in PBS (Gibco, 10010-023), collected in PBS and centrifuged for 5 min at 4 °C. Then the pellet was dried and frozen at −80 °C.

#### Sample preparation

Samples were allowed to fully thaw at room temperature. The cell pellets were digested using 0.075 ml trace-metal-grade concentrated NHO_3_ (69% w/w, respectively; Fisher Scientific TraceMetal Grade acid), which was added straight into the Eppendorf tubes provided. The sample was very lightly vortexed. Samples were digested in an oven at 60 °C for 1 h. The samples were allowed to cool, and 0.0375 ml TraceMetal Grade hydrogen peroxide (30% w/w, Merck) was added. The samples were digested further at 60 °C for 1 h in an oven. Samples were allowed to cool, and 1.375 ml purified water with a resistivity ≥18.2 MΩ cm from a Milli-Q system (Merck Millipore) was added to make up to a final volume of 1.5 ml.

### Preparation of PBS

Trace-metal-grade 15-ml tubes were labelled, and 0.1 ml of the PBS was aliquoted. The sample was digested in an oven at 60 °C using 0.1 ml trace-metal-grade concentrated HNO_3_ (69% w/w, respectively; Fisher Scientific TraceMetal Grade acid). The sample was cooled, and 0.05 m TraceMetal Grade hydrogen peroxide (30% w/w, Merck) was added. The samples were digested further at 60 °C for 1 h in an oven. Samples were allowed to cool, and 1.75 ml purified water with a resistivity ≥18.2 MΩ cm from a Milli-Q system (Merck Millipore) was added to make up to a final volume of 2 ml.

### Measurements

All measurements were conducted on a PerkinElmer NexION 5000 inductively coupled plasma–triple quadrupole mass spectrometer under dynamic reaction cell (DRC) mode at the London Metallomics Facility, King’s College London. The introduction system to the instrument was a PerkinElmer S23 autosampler coupled to a SeaSpray glass nebulizer fitted to a quartz cyclonic spray chamber. Argon plasma flow and nebulizer gas flow rates were 18 l min^−1^ and 0.98 l min^−1^, respectively. Samples were introduced to the system together with a carrier solution containing 0.785 M HNO_3_ and 50 µg l^−1^ Ga (internal standard). Quality control of inductively coupled plasma–triple quadrupole mass spectrometer measurements was ensured through a combination of repeat measurements of:

- Acid blanks
- A quality control standard made from 1,000 mg l^−1^ High-Purity Standards multi-element standard solutions
- A tenfold dilution of the CRM-TMDW (trace metal drinking water) from High-Purity Standards

Analyte measurements were normalized to the internal standard Iridium (^193^Ir) to account for instrument drift and matrix effects, and measurements were subsequently blank corrected by removing the average analyte intensity of repeat blank measurements. The corrected analyte intensity was converted to concentration measurements using the linear equation generated by the calibration curve of the standards.

### Iron measurement using the Iron Assay Kit

For total iron measurement, the Iron Assay Kit (Sigma-Aldrich, MAK472) was used, according to the manufacturer’s instructions.

### Measurement of intracellular glutathione levels

SK-MEL-103 and IMR90 cells were plated in 96-well plates, and senescence was induced for 6 days (cell density and senescence induction are presented in Supplementary Table 8). At day 6, cells were treated with BSO for 16 h, and then RSL3 or SCLA2 were added for 4 h. After 4 h, glutathione levels are measured using the GSH-Glo Glutathione Assay (Promega, V6911) according to the manufacturer’s instructions.

### Transfection of siRNAs and senolytic screen

Druggable genome siRNA libraries were purchased from Horizon Discovery (human siGENOME SMARTpool). Custom-designed siRNA library plates and individual siRNAs were obtained from the siGenome Human siRNA catalogue (Dharmacon, Horizon Discovery). All plates were supplied lyophilized and coated onto 96-well plates (0.1 nM) or in a tube (for individual siRNA orders). The siRNA 96-well plates (0.1 nM) were reconstituted in 100 μL nuclease-free water and aliquoted in round-bottom 96-well (Nunc, 163320) daughter plates for storage at −20 °C. For siRNA transfection, 3.6 μl of aliquoted siRNA was dispensed to flat-bottom 96-well plates (Nunc MicroWell, 167008). A ‘transfection mix’ was prepared with 17.2 μl plain DMEM (Gibco) and 0.4 μl DharmaFECT 1 transfection reagent (Horizon Discovery, T-2005-01) per well. A total of 17.6 μl of the transfection mix was added to each well already containing 3.6 μl siRNA (or 3.6 μl water for mock controls), and the mixture was incubated at room temperature for 30 min. For senolytic siRNA screening, SK-MEL-103 cells were first plated in 10-cm dishes (Supplementary Table 9). The following day, cells were treated with DMSO or etoposide for 6 days to generate proliferating and senescent cells. On day 6, cells were counted, and 100 μl cell suspension (in DMEM media supplemented with only 10% FBS but no antibiotics) was added to the 96-well plates (containing siRNA and transfection mix) at the appropriate seeding density (12k for DMSO and 15k for etoposide-treated cells). This resulted in a final siRNA concentration of 30 nM. Plates were incubated at 37 °C, 5% CO_2_, for 18 h to allow reverse transfection and cell attachment (day −1). After this period, the transfection media were discarded and replaced with fresh DMEM medium supplemented with 0.5% FBS and 1% antibiotic–antimycotic solution (day 0). A total of 3 or 5 days later, cells were fixed in 4% PFA for 45 min and then stained with DAPI, as described above. Cells were acquired with an InCell 2500 Analyser high-throughput microscope (GE Healthcare), as previously described. All the siRNAs used in the screen are presented in Supplementary Table 9.

### cDNA generation and RT–qPCR

cDNA was generated using random hexamers and SuperScript II reverse transcriptase (Invitrogen, 18064-014), according to the manufacturer’s protocol. Quantitative real-time PCR (RT–qPCR) was performed using SYBR Green PCR master mix (Applied Biosystems, 4309155) with QuantStudio 7 Flex (Applied Biosystems). The primers used for RT–qPCR are presented in Supplementary Table 10. The delta–delta Ct method (2^−∆∆Ct^) was applied to the resulting Ct values obtained during the amplification phase to calculate relative mRNA expression levels. GAPDH (human or mouse) and HPRT1 (human) were used as the housekeeping control for normalization, and an untreated sample was used as an internal relative control (DMSO). The 2^−∆∆Ct^ method calculation is as follows:

$$\Delta \Delta \mathrm{Ct}=\Delta {\mathrm{Ct}}_{{\rm{treated}}}-\frac{\Delta {\mathrm{Ct}}_{{\rm{control}}}}{\Delta }={\mathrm{Ct}}_{{\rm{target}}}-{\mathrm{Ct}}_{{\rm{housekeeping}}}.$$

The primers used are presented in Supplementary Table 10.

### siRNA knockdown validation by qRT–PCR

For siRNA knockdown validation by qRT–PCR, senescent and non-senescent SK-MEL-103 cells were generated as described before. At day 6, the transfection mix was prepared by mixing 72 μl of selected siRNAs (1 μM) with 344 μl plain DMEM and 8 μl DharmaFECT 1 per well (a six-well plate was used; Corning, 3506). After 30 min incubation, cells were added (500,000 for DMSO and 800,000 for etoposide-treated cells) in DMEM media supplemented with only 10% FBS (no antibiotics). Plates were incubated at 37 °C, 5% CO_2_, for 18 h to allow reverse transfection and cell attachment (day −1). After this period, the transfection media were discarded and replaced with fresh DMEM medium supplemented with 0.5% FBS and 1% antibiotic–antimycotic solution. A total of 48 h after transfection, cells were collected and RNA was extracted using the RNeasy Mini Kit (Qiagen, 74106), according to the manufacturer’s instructions.

### Drug screen to determine senolytic activity of electrophilic compounds

We screened 10,480 electrophilic compounds from the covalent screening library (CSL-10480-10-Y-10, Enamine). The primary screen was performed in IMR90 ER:RAS, a model of OIS. IMR90 ER:RAS was seeded in a 96-well plate at the density presented in Supplementary Table 8. Senescence was induced with 100 nM 4-OHT for 7 days, while non-senescent cell controls were obtained by treating cells with DMSO. At that point, compounds were added at a final concentration of 10 μM, diluted in DMEM with 0.5% FBS. After 72 h, cells were fixed in 4% PFA for 45 min and then stained with DAPI for 15 min. Cells were imaged with an InCell 2500 Analyser high-throughput microscope (GE Healthcare) as previously described. A compound was classified as senolytic if it exhibited at least a 20% greater cytotoxicity in senescent cells compared with non-senescent cells.

### Retesting of senolytic compounds in SK-MEL-103 cells

SK-MEL-103 cells were used to assess the senolytic activity of the hits discovered in the primary screening. Senescence was induced with etoposide or tozasertib treatment (Supplementary Table 8). After 6 days of senescence induction, electrophilic compounds were added at different concentrations, diluted in DMEM with 0.5% FBS. After 48 h, cells were fixed with 4% PFA for 45 min and then stained with DAPI for 15 min. Cells were acquired with an InCell 2500 Analyser high-throughput microscope (GE Healthcare), as previously described.

### Drug screen in SK-MEL-103 with selected electrophilic compounds in combination with cell death inhibitors

Selected electrophilic compounds were tested in SK-MEL-103 cells in the presence of apoptosis or ferroptosis inhibitors (emricasan and liproxstatin 1, respectively). Senescence was induced with etoposide for 6 days (Supplementary Table 8). At this point, compounds were added at 5 μM, either alone or in combination with emricasan (25 μM) or liproxstatin 1 (1 μM). All senolytic compounds were diluted in DMEM with 0.5% FBS. After 48 h, cells were fixed with 4% PFA for 45 min and then stained with DAPI for 15 min. Cells were imaged using an InCell 2500 Analyser high-throughput microscope (GE Healthcare), as previously described.

### Sample preparation for ABPP

SK-MEL-103 cells were seeded in 10-cm dishes as presented in Supplementary Table 8 and treated with etoposide, tozasertib or DMSO for 6 days. On day 6, cells were treated with DMSO or the corresponding SCLA for 2 h, followed by the indicated alkyne-modified probes for 2 h, washed with ice-cold PBS three times, cell pellets collected by scraping and stored at −80 °C until further processing. See Supplementary Table 3 for the respective compound concentrations for each probe set.

Pellets were thawed on ice for 1 h and lysed in 200 μl 1% SDS in PBS supplemented with 1× protease inhibitors (cOmplete, EDTA-free Protease Inhibitor Cocktail, Roche, 11836153001) and shaken for 30 min at room temperature with 1:1,000 v/v benzonase nuclease (Sigma, E1014). Protein concentration was measured by DC Protein Assay (Bio-Rad, 5000111) and normalized to 1.5 mg ml^−1^, with 100 µg protein taken forward. Click reaction mixture was prepared by sequential addition of 1 volume 10 mM azide-PEG3-biotin (Sigma, 762024), 2 volumes 50 mM CuSO_4_ (Sigma, C8027), 2 volumes 50 mM TCEP (Sigma, 68957) and 1 volume 10 mM TBTA (Sigma, 678937). A final volume of 6 µl click reaction mixture was added per 100 µl lysate and shaken for 1 h at room temperature before quenching with 5 mM EDTA. Proteins were then precipitated by the addition of the total sample volume to 4× volume liquid chromatography–mass spectrometry (LC–MS)-grade acetonitrile, as per the SP4 protocol^63^, followed by gentle shaking for 5 min and centrifugation at 3,220*g* for 10 min. The subsequent pellet was washed with 80% LC–MS-grade ethanol in water and centrifuged at 3,220*g* for 2 min for a total of four washes. The remaining pellet was allowed to dry for 5 min and resuspended in 10 µl 10% SDS, followed by sequential dilution to 25, 50 and 100 µl final volume with 50 mM HEPES (pH 8). Samples were centrifuged at 3,220*g* for 2 min to check that no insoluble material remained.

Before biotin–streptavidin pulldown, magnetic streptavidin beads (Thermo Fisher Scientific, 88817) were washed three times in 0.2% SDS in 50 mM HEPES. In total, 10 µl washed beads were then added to each resuspended pellet and shaken at room temperature for 2 h to form the biotin–streptavidin interaction.

For western blot-based competitive ABPP with RSL3-alkyne or SCLA2a samples were washed in 500-µl solutions of: 1x 500 µl 1% SDS in 50 mM HEPES and 2x 500 µl 0.2% SDS in 50 mM HEPES. Washed beads were eluted in 20 μl 0.2% SDS in 50 mM HEPES with 4× Laemmli sample buffer (Bio-Rad, 1610747) and boiled at 95 °C for western blot analysis.

For MS-based analysis, subsequent wash steps were performed on a Kingfisher Flex equipped with a 96-well Deep Well head. Beads were sequentially washed in 500-µl solutions of: 1x 1% SDS in 50 mM HEPES, 1x 500 µl 0.2% SDS in 50 mM HEPES, 1x 500 µl 0.2% SDS, 40 mM CAA, 10 mM TCEP in 50 mM HEPES (10-min incubation with agitation) and finally 2x 500 µl 50 mM HEPES. Washed beads were finally deposited in 100 µl 50 mM HEPES. In total, 200 ng trypsin (Promega, Sequencing-Grade Trypsin, V5111) was added to each sample and shaken overnight at 37 °C. Peptide digests were acidified to pH 2–3 with LC–MS-grade formic acid (FA; Fisher, 10780320) and stored until LC–tandem MS (MS/MS) analysis. For Evotip loading, any remaining beads were removed by plate-based filtration (Merck, 10448023), and half the filtered digest volume was loaded on Evotips according to the manufacturer’s instructions.

### Total proteomics in SK-MEL-103 cells undergoing senescence and treated with SCLA2/2a/2b

SK-MEL-103 cells grown in six-well plates were treated with either etoposide or tozasertib to induce senescence or DMSO as a control for 6 days (Supplementary Table 8). On day 6, cells were treated with SCLA2, SCLA2a, SCLA2b or DMSO as indicated for 2 h at the concentrations presented in Supplementary Table 3. Cells were then lysed in 80 µl surfactant cocktail data^64^ with 4 µl benzonase added to each plate at 5 U µl^−1^ (diluted 1:50 from 250 U µl^−1^ stock) right before lysis, to prevent benzonase inactivation by the lysis buffer. Samples were transferred to a 96-well plate and frozen at −80 °C. Samples were thawed at 60 °C in a thermomixer and mixed using a Gilson Platemaster. Protein concentrations were measured with the BCA assay and adjusted to 2 µg µl^−^ in 40 µl with the Andrew Plus pipetting robot from Waters. Sample preparation was performed in Brand PCR 96-well plates. For reduction and alkylation, 20 µl of each sample corresponding to 40 µg of protein was mixed with 20 µl of a solution of 40 mM CAA–20 mM TCEP and incubated at 37 °C for 30 min. Proteins were precipitated with 160 µg of Hydroxyl beads (MR-HYX010) using the Protein Aggregation Capture (PAC) method, with precipitation carried out in 60% ethanol and three washes in 80% ethanol^65^. Samples were digested overnight at 37 °C in 50 µl of 50 mM ammonium bicarbonate, shaking the plate at 1,650 rpm with trypsin and LysC at a 1:50 and 1:100 ratio, respectively. This experiment is shown in Fig. 5b and Extended Data Figs. 4d and 6e.

### Data acquisition for ABPP

Peptides were analysed by LC–MS/MS using an Evosep One (Evosep) coupled with a timsTOF HT (Bruker) equipped with either an 8 cm × 150 µm, 1.5 µm analytical column (Evosep, 60SPD, ABPP samples) or a 15 cm × 150 µm, 1.9 µm analytical column (Evosep, 30SPD, SK-MEL-103 total proteome samples). Peptides were separated by the Evosep 30/60SPD workflow (analytical solvents A: 0.1% FA and B: acetonitrile plus 0.1% FA). The column was held at 40 °C. Data were acquired in data-independent acquisition PASEF mode with the following settings: *m*/*z* range from 100 *m*/*z* to 1,700 *m*/*z*, ion mobility range from 1/K_0_ = 1.30–0.85 V s cm^−^^2^ using equal ion accumulation and ramp times in the dual TIMS analyser of 100 ms each. Each cycle consisted of eight PASEF ramps covering 21 mass steps each with 25-Da windows, each with 2/3 non-overlapping ion mobility windows covering the 475–1,000 *m*/*z* range and 0.85–1.26 V s cm^−^^2^ ion mobility range. The collision energy was lowered as a function of increasing ion mobility from 59 eV at 1/K_0_ = 1.6 V s cm^−^^2^ to 20 eV at 1/K0 = 0.6 V s cm^−^^2^.

### Data acquisition for total proteomics in SK-MEL-103 cells treated with SCLA2/2a/2b

For each sample, 2 µg digest was analysed. Chromatographic separation was performed using an Ultimate 3000 RSLC nano LC system (Thermo Scientific) coupled to a Q Exactive HF-X MS (Thermo Scientific) via an EASY-Spray source. Electrospray nebulization was achieved by interfacing to Bruker PepSep emitters (part no. PSFSELJ20, 20 µm). Peptide solutions were injected directly onto the analytical column (self-packed column, CSH C18 1.7 µm beads, 300 μm × 30 cm) at a working flow rate of 5 μL min^−1^ for 4 min. Peptides were then separated using a 60-min stepped gradient: 0–45% of buffer B for 70 min (buffer A: 95%/5%: H_2_O/DMSO + 0.1% FA; buffer B: 75%/20%/5% MeCN/H_2_O/DMSO + 0.1% FA), followed by column conditioning and equilibration. Eluted peptides were analysed by the MS in positive polarity using a data-independent acquisition mode as follows: an initial MS1 scan was carried out at 140,000 resolution with an automatic gain control target of 3 × 10^6^ for a maximum injection time of 200 ms, *m*/*z* range: 410–1,600. This was followed by 30 30k-resolution MS2 scans covering 410–1,600 with variable window sizes^66^. The automatic gain control target was set to 3 × 10^6^ with maximum injection time on auto. Normalized collision energy was set to 27%. Total run acquisition time was 70 min.

### Data processing and downstream analysis for ABPP in SK-MEL-103 senescence

Raw.d files were searched in Spectronaut 19 in directDIA mode against the canonical UniProt human FASTA (downloaded 20240122, 20,596 entries) according to default modifications and settings with the following amendments: no cross-run normalization or imputation was applied, and protein inference was set to ‘all matching proteins’.

Downstream data analysis was performed in R (4.5.1, 2025-06-13). For ABPP analyses only, outlier samples (based on low precursor identifications or clustering by principle coordinate analysis) were removed, leaving a minimum of 3/4 samples per condition. Contaminant proteins^67^ and protein groups detected in <3 replicates per condition were removed. Protein group quantities were then log_2_transformed and median normalized per sample. Differential abundance testing was performed by Welch’s *t*-test (*n* = 3/4, as implemented in the protti R package^68^). Further data analysis and visualization were performed with tidyverse^69^, ggplot^70^ and ComplexHeatmap^71^ packages.

### Data processing and downstream analysis for total proteomics in SK-MEL-103 cells treated with SCLA2/2a/2b

Data were processed using the Spectronaut software platform (Biognosys, 19.9.250324.62635) in directDIA mode. A Pulsar Search was conducted with a missed cleavage rate set to 3. Cys carbamidomethylation was applied as a fixed modification, while variable modifications included Met oxidation, Gln deamidation, protein N-terminal acetylation and the cyclization of glutamine to pyroglutamate. Identification of peptide spectrum matches, peptides and protein groups was performed with a false discovery rate (FDR) of 0.01. Searches were carried out against the UniProt *Homo sapiens* database, containing one gene per protein sequence (downloaded on 8 January 2025, consisting of 20,650 entries), and a universal protein contaminants database (downloaded on 13 January 2025, containing 381 entries).

For the data-independent acquisition analysis, a mutated decoy database approach was used, with a protein *q*-value cut-off for the experiment set at 0.01 at the identification level. The quantification method was configured as follows: MS2-based quantification with a proteotypicity filter set to ‘only proteotypic,’ and the label-free quantification method set to MaxLFQ. No value imputation was used.

Before further analysis, protein tables were filtered to remove entries from the protein contaminants database^67^. The Uniprot ID mapping service (https://www.uniprot.org/id-mapping) was used to retrieve protein metadata, for example, Gene Ontology (GO) terms GOMF, GOCC and GOBP, against a list of protein accession IDs. Metadata were then merged into the main protein table. Further analysis was carried out in the Perseus^72^ platform (v2.1.3.0). Local normalized protein intensity was log_2_transformed before additional filtering and statistical testing. For two-group comparisons, intensities were filtered for proteins with ≥3 replicate intensities per experimental group, and Welch’s *t*-test was carried out (multiple testing correction: permutation-based FDR, threshold, 0.05). The *t*-test results were visualized as volcano plots, with significance defined by *P* value.

### RNA isolation and purification from tumour tissues

Tumours were homogenized mechanically with pestles (Corning, PES-15-B-SI) in 500 μl TRIzol (Ambion, Life Technologies, 15596026). Next, 100 μl chloroform (Sigma, 319988) was added, and the samples were centrifuged at 12,000 rpm for 30 min. The transparent phase was collected and mixed with an equal volume of 70% ethanol. The samples were then moved to the RNAeasy Mini Kit column, and the RNA was extracted according to the manufacturer’s protocol.

### RNA isolation and purification

For total RNA isolation, cells were seeded in six-well plates. Cells were dissociated using trypsin, washed with PBS and lysed. Total RNA from the cell pellet was isolated and purified using the RNeasy Mini Kit (Qiagen, 74106), according to the manufacturer’s instructions. RNA was eluted in 30–50 μl RNAse-free water, and concentration was measured using a Nanodrop ND-1000 UV-Vis spectrophotometer. RNA samples were stored at −80 °C for long-term preservation.

### RNA sequencing and analysis

Total RNA was extracted using the RNeasy Mini kit (Qiagen) following the manufacturer’s protocol. RNA sequencing was performed by the MRC LMS Genomics core facility. Library preparation was performed using the NEBNext Ultra II Directional RNA library prep kit with the NEBNext PolyA Enrichment module using the NextSeq 2000 instrument model (single end 72 base pairs (bp) + dual 8 bp indexing). RNA sequencing data were aligned using STAR. For analysis of the RNA sequence of the PC3 xenograft tumour model, sequencing reads were mapped separately to human (GRCh38) and mouse (GRCm37) genomes using the STAR aligner (version 2.7.11a)^73^. R-package XenofilteR^74^ was then used to separate mouse reads from human sequence reads. Gene-based counting was performed on the human reads using the featureCount function from the Rsubread package^75^. Differential expression analysis was performed using the DESeq2 Bioconductor package (DESeq2_1.48.2)^76^ in R. A List of differentially expressed genes was obtained along with log_2_ fold change over the control group with an adjusted *P* value for each gene. For GSEA, differentially expressed genes were ranked according to their expression using the preranked function on GSEA v4.0.3 (Broad Institute). Significant enrichment of gene sets was considered if the normalized enrichment score (NES) was >1.0, with an FDR lower than 0.25 (ref. ^77^). Gene sets used were obtained from built-in ‘Gene Ontology’ or ‘curated’ databases v7.5 (Broad Institute) or described previously^78^. Cluster and pathway analyses were performed using ClusterProfiler on R.

### Western blot

Cells were lysed in 1× RIPA buffer (Thermo Scientific, 89900) supplemented with cOmplete mini EDTA-free protease inhibitor tablets (Roche, 04693159001) and PhosSTOP phosphatase inhibitor tablets (Roche, 04906837001) and incubated on ice for 10 min. Lysates were centrifuged at 14,000 rpm for 15 min, and protein concentration was determined using the BCA kit (Thermo Fisher, 23227). Equal amounts of protein were loaded on SDS–polyacrylamide gel electrophoresis (SDS–PAGE), using Mini-PROTEAN TGX gels (Bio-Rad, 4561096) and transferred to a 0.2-mm nitrocellulose membrane (Bio-Rad, 1620112). After protein transfer, membranes were blocked in 5% milk solution and incubated overnight at 4 °C with the indicated primary antibodies. Membranes were then probed with horseradish peroxidase-conjugated secondary antibodies antirabbit IgG (Abcam, A16035, 1:5,000) or antimouse IgG (Abcam, A16011, 1:5,000) and developed using enhanced chemiluminescence substrate (Bio-Rad, 170-5060). Membranes were exposed to the Amersham imageQuant 800 imaging system (Cytiva). Blots were semiquantitatively analysed by densitometry using ImageJ 2.9/1.53t (National Institutes of Health). A list of the antibodies used in this study is presented in Supplementary Table 6.

### HDTVI

Hydrodynamic tail vein injection (HDTVI) was performed to deliver transposon-based vectors as previously described with minor modifications^53^. For assessing GPX4 expression, we used samples derived from female C57BL/6J (Charles River Laboratories UK, RRID: IMSR_JAX:000664) mice transduced at 5–6 weeks of age. For the RSL3 treatment experiment, HDTVI was carried out in 8-week-old C57BL6/J male mice. Plasmid DNA was prepared using the Qiagen Plasmid Maxi Kit or GenElute HP Endotoxin-Free Maxiprep kit (Sigma) according to the manufacturer’s instructions. On day 0, 25 µg Nras^G12V^ plasmid and 5 µg Sleeping Beauty transposase expression vector were diluted in sterile normal saline to a final volume of 2 ml (~10% of body weight). The solution was then injected into the lateral tail vein within 10 s. For the RSL3 experiment, starting from day 5, the mice then received daily i.p. injection of RSL3 (10 mg kg^−1^) or vehicle for five consecutive days. Livers were collected 10 days after HDTVI. RSL3 (MedChemExpress, HY-100218A) was dissolved in 10% DMSO and 90% 20% SBE-ß-CD in saline and was administered i.p.

### ID8 *Trp53*^−/−^ model of ovarian cancer

For the ovarian cancer experiments, C57BL/6J female mice (6–7 weeks old) were purchased from Charles River Laboratories UK (RRID: IMSR_JAX:000664) and allowed to acclimatize for at least 1 week before any regulated procedures were performed. Each mouse received an i.p. injection of 5 × 10^6^ ID8 *Trp53*^−/−^ cells suspended in sterile and filtered PBS (Thermo Fisher Scientific, J61196-AP). After 21 days, mice were randomized into four different experimental treatment groups: vehicle, cisplatin (4 mg kg^−1^ once per week for 1 week), RSL3 (50 mg kg^−1^, two times per week for 2 weeks) and cisplatin in combination with RSL3. All treatments were administered via i.p. injection. At the defined end point, omental tumours were collected. Cisplatin was dissolved in PBS. RSL3 (MedChemExpress, HY-100218A) was dissolved in 10% DMSO, 40% PEG300, 5% Tween-80 and 45% saline. For the survival experiment, mice were checked daily and were culled when they showed visible distress symptoms such as weight loss of 10% or more, ascites equivalent to full-term pregnancy, reduced or slow activity, pale feet, hunching, piloerection, closed eyes and isolation from cage mates.

### PC3 model of prostate cancer

For the prostate cancer experiments, 12-week-old NRG male mice were used. Each mouse received an s.c. injection of 2.5 × 10^6^ PC3 cells suspended in sterile and filtered PBS. When the tumours reached a volume of approximately 100 mm^3^, mice were randomized into two treatment groups: vehicle and palbociclib (200 mg kg^−1^ three times per week). Once the tumour became arrested in the palbociclib-treated group, mice were further randomized to receive either RSL3 (50 mg kg^−1^, two times per week for 2 weeks) or the vehicle, resulting in four experimental groups: vehicle, RSL3, palbociclib and palbociclib + RSL3. Mice were culled when the tumours in the vehicle groups reached a volume of 1,500 mm^3^, and tumours were collected. Palbociclib (monohydrochloride; MedChemExpress, HY-50767A) was dissolved in 10% DMSO, 40% PEG300, 5% Tween-80 and 45% saline and administered via oral gavage. RSL3 (MedChemExpress, HY-100218A) was dissolved in 10% DMSO, 40% PEG300, 5% Tween-80 and 45% saline and administered i.p. Vehicle-treated mice received 10% DMSO, 40% PEG300, 5% Tween-80 and 45% saline.

### SK-MEL-103 model of melanoma

For the melanoma experiments, 12-week-old NRG female mice were used. Each mouse received an s.c. injection of 1 × 10^6^ SK-MEL-103 cells suspended in 100 μl sterile PBS/Matrigel. When the tumours reached a volume of approximately 50 mm^3^, mice were randomized into two treatment groups: vehicle and palbociclib (100 mg kg^−1^ five times per week). Once the tumour became arrested in the palbociclib-treated group, mice were further randomized to receive either RSL3 (50 mg kg^−1^, five times per week for 2 weeks) or the vehicle, resulting in four experimental groups: vehicle, RSL3, palbociclib and palbociclib + RSL3. Palbociclib treatment was kept during RSL3 administration. Mice were culled when the tumours reached a volume of 1,500 mm^3^, and tumours were collected. Palbociclib (monohydrochloride; MedChemExpress, HY-50767A) was dissolved in 10% DMSO, 40% PEG300, 5% Tween-80 and 45% saline and administered via oral gavage. RSL3 (MedChemExpress, HY-100218A) was dissolved in 10% DMSO, 40% PEG300, 5% Tween-80 and 45% saline and administered i.p. Vehicle-treated mice received 10% DMSO, 40% PEG300, 5% Tween-80 and 45% saline.

### IF and IHC staining of tissue sections

Tumour tissue sections were deparaffinized in Histo-Clear (Scientific Laboratory Supplies) for 5 min, rehydrated through a series of decreasing ethanol concentrations, followed by a final 5 min wash in distilled water (dH_2_O). Heat-induced epitope retrieval (HIER) was performed in a pressure cooker for 20 min using either citrate-based buffer at pH 6.0 (VectorLab, H-3300-250) or tris-based buffer at pH 9.0 (VectorLab, H-3301-250), according to the antibody manufacturer’s instructions. For intracellular antigen staining, sections were permeabilized with 0.2% Triton X-100 in PBS for 10 min and washed in PBS for 5 min. Slides were then incubated in BLOXALL blocking solution (VectorLab, SP-6000) for 15 min, washed in PBS and blocked with Animal-Serum Free serum (Cell Signaling, 15019L) diluted in dH_2_O for 45 min. Tissue samples were incubated overnight with the primary antibody previously diluted in antibody diluent (Agilent Technologies, S080983-2). Sections were washed in PBS three times for 5 min each and were incubated with secondary antibody for 45 min.

#### IF

For double staining, samples were incubated in 0.02 N HCl for 20 min following the first antibody signal amplification. Sections were washed in PBS for 5 min, and peroxidase blocking was reapplied for 15 min. Animal-serum-free blocking solution (Cell Signaling Technology, 15019L) diluted in H_2_0 was applied for 1 h, followed by incubation with the second primary antibody overnight. After three 5-min washes in PBS, sections were incubated with the corresponding secondary antibody for 45 min. Tyramide signal amplification was performed using SuperBoost kit (Invitrogen, B40935 for Alexa Fluor 596 and B40932 for Alexa Fluor 488) according to the manufacturer’s instructions and applied to the section for 10 min at room temperature. Sections were then washed three times in PBS for 5 min and counterstained with DAPI (1 μg ml^−1^ DAPI in PBS) for 5 min. Following three additional PBS washes (5 min each), slides were mounted in 50% glycerol in PBS. Images were acquired using a 20× fluorescence objective on the Zeiss AxioScan Z.1 slide scanner. Stainings were quantified in QuPath by measuring positive cells as a percentage of the total tissue area. All antibodies used for IF are presented in Supplementary Table 6.

#### IHC

After incubation with secondary antibody, slides were washed in PBS and incubated in SignalStain DAB (CST, 8059) for 5 min or until the horseradish peroxidase signal was visible and the reaction stopped in dH_2_O. Cells were then stained with hematoxylin (DAKO, Mayer’s Hematoxylin, S3309) for 30 s, washed in ammonia for 30 s and then washed in dH_2_O. Slides were dehydrated in 75% ethanol for 1 min and 100% ethanol for 5 min, washed in Histo-Clear for 5 min and mounted in DPX (Sigma-Aldrich). All antibodies used for immunohistochemistry (IHC) are presented in Supplementary Table 6.

### GL13 staining

GL13 (SenTraGor) staining was performed as previously described^55^. In brief, formalin-fixed, paraffin-embedded xenograft tumour sections were deparaffinized and rehydrated through graded alcohols. Antigen retrieval was carried out by heat-mediated treatment in 10 mM citric acid buffer (pH 6.0) using a steamer for 15 min, followed by cooling in an ice bath for 20 min. Endogenous peroxidase activity was quenched by incubation with 3% hydrogen peroxide (H_2_O_2_) for 18 min at room temperature. Sections were subsequently washed and incubated sequentially in 50% and 70% ethanol for 5 min each. The samples were then treated with the SenTraGor reagent at 37 °C for 10 min. To remove residual reagent or precipitates, sections were washed with 50% ethanol for 2–3 min, followed by PBS and 0.3% Triton X-100 in PBS for 4 min. After washing with PBS, sections were incubated with a primary antibiotin antibody (1:300 dilution; Hyb-8, ab201341, Abcam, Cambridge, UK) for 1 h at room temperature. Signal development was performed using the Dako REAL EnVision Detection System (cat. no. K5007) according to the manufacturer’s instructions, using 3,3′-diaminobenzidine (DAB) as the chromogen to produce a brown signal. The mean percentage of SenTraGor-positive cells was quantified from at least five high-power fields (40× objective) per sample using a ZEISS Axiolab 5 optical microscope.

### GLF16 staining

GLF16 staining was performed as previously described^56,79^. Formalin-fixed, paraffin-embedded tissue sections were deparaffinized, rehydrated and subjected to antigen retrieval as described in the SenTraGor staining section. The GLF16 reagent (70 μg ml^−1^) was applied to the tissue sections for 10 min in dark conditions at room temperature. Samples were subsequently washed three times for 10 min each with GLF16 diluent (PBS, 2.5% DMSO and 2.5% Tween-20). Following PBS washes, tissue autofluorescence was quenched using the Vector TrueVIEW Autofluorescence Quenching Kit according to the manufacturer’s instructions. Nuclei were counterstained with DAPI, and sections were briefly rinsed with dH_2_O (30 s) before coverslipping. IF images were acquired using a Leica TCS-SP8 confocal microscope. The mean percentage of GLF16-positive cells was quantified from at least five high-power fields (40× objective) per sample.

### SA-β-galactosidase assay in tumour samples

Tumour samples frozen in OCT were cryosectioned at 15 μM. Sections were fixed in ice-cold 0.5% glutaraldehyde (w/v, PBS) for 15 min and washed with 1 mM MgCl_2_/PBS (pH 6.0) for 5 min. β-galactosidase activity was detected using X-Gal staining solution (1 mg ml^−1^ X-gal, Thermo Scientific, 5 mM K_3_(Fe(CN)_6_) and 5 mM K_4_(Fe(CN)_6_)) diluted in 1 mM MgCl_2_/PBS (pH 5.5–6) for 8–18 h at 37 °C. Slides were dehydrated, mounted using VectaMount Permanent mounting media (Vector Laboratories, H-5000-60) and imaged using ×20 bright-field objective on a Zeiss AxioScan Z.1 slide scanner.

### Lipid extraction for bulk lipidomics

Cells were quenched with ice-cold methanol after treatment and transferred to tubes. Methanol was evaporated, and pellets were sonicated for 5 min in chloroform:methanol (2:1 v/v, 1 ml). Lipid extraction was performed using the Folch method^80^. In brief, LC–MS-grade water (400 µl) was added to the homogenized cells in organic solvent and thoroughly mixed. Separation of the polar and non-polar fractions was achieved by centrifugation (13,200*g*, 15 min). The separate fractions were dried down under vacuum. The organic layer was reconstituted in chloroform:methanol (2:1 v/v, 200 µl and diluted 1:10 with 2:1:1 isopropanol:acetonitrile:water, with the addition of an internal standard mix composed of isotopically labelled compounds from major lipid classes. Internal standard mix (final concentration 0.5 µg ml^−1^) included: N-palmitoyl-d31-*d*-erythro-sphingosine (C16-d31 ceramide), pentadecanoic-d29 acid (15:0-d29 free fatty acid (FFA)), heptadecanoic-d33 acid (17:0-d33 FFA), eicosanoic-d39 acid (20:0-d39 FFA), tetradecylphosphatidylcholine-d42 (14:0-d29 LPC-d13), 1-palmitoyl(d31)-2-oleyl-*sn*-glycero-3-phosphatidylcholine (16:0-d31–18:1 PC), 1-palmitoyl(d31)-2-oleyl-*sn*-glycero-3-phosphoethanolamine (16:0-d31–18:1 PE), 1-palmitoyl-d31–2-oleoyl-*sn*-glycero-3-[phospho-rac-(1-glycerol)] (16:0-d31–18:1 PG), 1-palmitoyl-d31–2-oleoyl-*sn*-glycero-3-phosphoinositol (16:0-d31–18:1 PI), N-palmitoyl(d31)-*d*-erythro-sphingosylphosphorylcholine (16:0-d31 SM), glyceryl-tri(pentadecanoate-d29) (45:0-d87 TAG), glyceryl-tri(hexadecanoate-d31) (48:0-d93 TAG) (all from Avanti Polar Lipids). PC, phosphatidylcholine; PE, phosphatidylethanolamine; PG, phosphatidylglycerol; PI, phosphatidylinositol; SM, sphingomyelin; TAG, triacylglycerol.

### MS for lipid detection

Sample (5 µl) was injected onto a C18 BEH column (2.1 mm × 50 mm, 1.7 µm; Waters) and separated using ultra HPLC on an Accela 3000 UHPLC (Thermo Fisher Scientific) using gradient elution at 0.5 ml min^−1^ and column temperature 55 °C. Mobile phase A was 60:40 acetonitrile:water, and mobile phase B was 90:10 isopropanol:acetonitrile. Both A and B contain 10 mM ammonium acetate (negative ion mode) or 10 mM ammonium formate (positive ion mode). The starting mobile phase was 40% B, increasing to 99% B over 8.5 min, before returning to starting conditions for 1.5 min. ultra HPLC was coupled to an LTQ Orbitrap Elite (Thermo Fisher Scientific) MS. The heated electrospray ionization source was operated at 375 °C and 2 or 3.5 kV for negative and positive ion modes, respectively. The capillary temperature was 380 °C; sheath and auxiliary gases were 60 and 20 arbitrary units, respectively. Spectra were obtained over a range of 200–2,000 *m*/*z* at 60,000 mass resolution. Data were converted to mzXML using ProteoWizard. Features were identified using xcms^81^ with 10 parts per million (ppm) spectral binning. Peak areas were normalized to the total ion count. Lipid identification was performed by mass matching to an in-house database and LIPID MAPS structure database^82^, and fatty acid composition of ether PE lipids was confirmed by MS/MS where possible. Annotated lipids were imported into MetaboAnalyst software (version 6.0: https://www.metaboanalyst.ca/). The heat map was generated in Metaboanalyst using the Average clustering method with Euclidean distance measure. Features were centred and scaled using the ‘autoscale’ function, and the top 50 hits based on their significance (analysis of variance (ANOVA)) were calculated.

### Synthesis of probes

All commercially available reagents and solvents were used without additional purification unless stated otherwise. Air and moisture-sensitive reactions were carried out in dried suba-sealed flasks purged with nitrogen. Thin-layer chromatography was performed on aluminium-backed plates coated with Merck DC Kieselgel 60 F254 and visualized under ultraviolet light at 254 nm. For LC–MS, the Agilent Infinity II 1260 LC–MS system or the Agilent MSD XT coupled to 1260 Infinity II HPLC was used. MS spectra were recorded in either positive or negative mode, as indicated. Flash column chromatography was carried out using glass chromatography columns packed with 40–63 μm silica gel or with the Biotage Selekt high-performance automated flash purification system. NMR spectra were recorded at room temperature (20 ± 1 °C) on Bruker 400 or 500 MHz. ^1^H NMR data are presented with the following: Chemical shifts (δ) are reported in ppm relative to residual solvent peaks as internal standards, multiplicity (s = singlet, d = doublet, t = triplet, q = quartet, m = multiplet and br = broad), coupling constants (*J*) in Hz and integration. High-resolution MS was performed using electrospray ionization and time-of-flight mass analysis. The purity of all final compounds was >95%, as defined by LC–MS and NMR. Detailed information on the synthesis and NMR spectra of the synthesized probes is provided in Supplementary Note 1.

### Statistics and reproducibility

Statistical analyses were performed and plotted using GraphPad Prism 10.5.0 software. Details of the test used are given in the corresponding figure legends and the Source Data. Statistical analysis was performed using either an unpaired, two-tailed *t*-test, paired *t*-test or an ordinary one-way or two-way ANOVA with Dunnett’s, Šídák’s or Tukey’s multiple comparison correction. All data points are plotted together with an overlay of the mean ± s.d. or s.e.m. (as specified in the figure legends). *P* < 0.05 was taken to indicate statistical significance. The number of biological replicates is indicated in their respective figure legends. For the dose–response curves, the number of biological replicates is indicated in the respective figure legends and Source Data. EC_50_ is determined as the geometric mean of the EC_50_ values from individual replicates. The standard error of the geometric mean is calculated using the following steps:

- Take the natural logarithm (ln) of the EC_50_ for each replicate
- Calculate the s.d. of the ln-transformed EC_50_ values
- s.d. of ln-transformed EC_50_ values/number of replicates

No statistical methods were used to predetermine sample sizes for the in vivo studies, but our sample sizes are similar to what we have reported previously, which aimed to reach a statistical power of at least 80%. For in vivo studies, mice were randomized to treatment groups 21 days after injecting the tumour cells (ID8 cells) or when the tumours reached a volume of approximately 100 mm^3^ (PC3) or 50 mm^3^ (SK-MEL-103). Cell culture experiments were not randomized. Investigators were not blinded during the cell culture or in vivo experiments, but whenever possible (for example, quantitative IF and IHC analysis), analysis was performed using automated procedures.

### Reporting summary

Further information on research design is available in the Nature Portfolio Reporting Summary linked to this article.

## Online content

Any methods, additional references, Nature Portfolio reporting summaries, source data, extended data, supplementary information, acknowledgements, peer review information; details of author contributions and competing interests; and statements of data and code availability are available at 10.1038/s41556-026-01921-z.

## Supplementary information

Supplementary Information Index referring to the Supplementary Information: Tables 1–10 (included as an independent Excel file), Figs. 1–6 and Note 1 (included as a separate PDF file).

Reporting Summary

Supplementary Table 1 Screen data.

Supplementary Table 2 Retesting data.

Supplementary Table 3 Concentration of compounds and probes used in pulldown experiments.

Supplementary Table 4 Proteomics data.

Supplementary Table 5 Lipidomics data.

Supplementary Table 6 Antibodies.

Supplementary Table 7 Drugs.

Supplementary Table 8 Conditions for senescence induction.

Supplementary Table 9 siRNAs.

Supplementary Table 10 qRT–PCR primers.

Supplementary Table 11 Light microscopy reporting table.

Supplementary Note 1 Probes synthesis.

Supplementary Data 1 Supplementary source data.

## Source data

Source Data Figs. 1–8 and Extended Data Figs. 1, 2, 4 and 5–10 Statistical source data.

Source Data Fig. 3 Uncropped western blots.

Source Data Extended Data Fig. 5 Uncropped western blots.

Source Data Extended Data Fig. 6 Uncropped western blots.

Source Data Extended Data Fig. 7 Uncropped western blots.

## Data Availability

RNA sequencing data that support the findings of this study have been deposited in the Gene Expression Omnibus (GEO) under accession codes GSE307575 and GSE313741. Mass spectrometry data have been deposited in ProteomeXchange with the primary accession codes PXD068417 and PXD068572. The lipidomics data are provided in Supplementary Table 5 and have been deposited in MassIVE under reference no. MSV000099167. All other data supporting the findings of this study are available from the corresponding author on reasonable request. Source data are provided with this paper.
