# Supplementary material for: Electrophilic compound screening identifies GPX4-dependent ferroptosis as a senescence vulnerability
Source: Nat Cell Biol. 2026 Apr 24;28(5):915–29. doi: 10.1038/s41556-026-01921-z (PMC13179136; doi:10.1038/s41556-026-01921-z)
Supplement: Supplementary file 1 — Index referring to the Supplementary Information: Tables 1–10 (included as an independent Excel file), Figs. 1–6 and Note 1 (included as a separate PDF file). [file 41556_2026_1921_MOESM1_ESM.pdf]

nature cell biology

Supplementary information https://doi.org/10.1038/s41556-026-01921-z

Electrophilic compound screening identifies GPX4-dependent ferroptosis as a senescence vulnerability

In the format provided by the authors and unedited

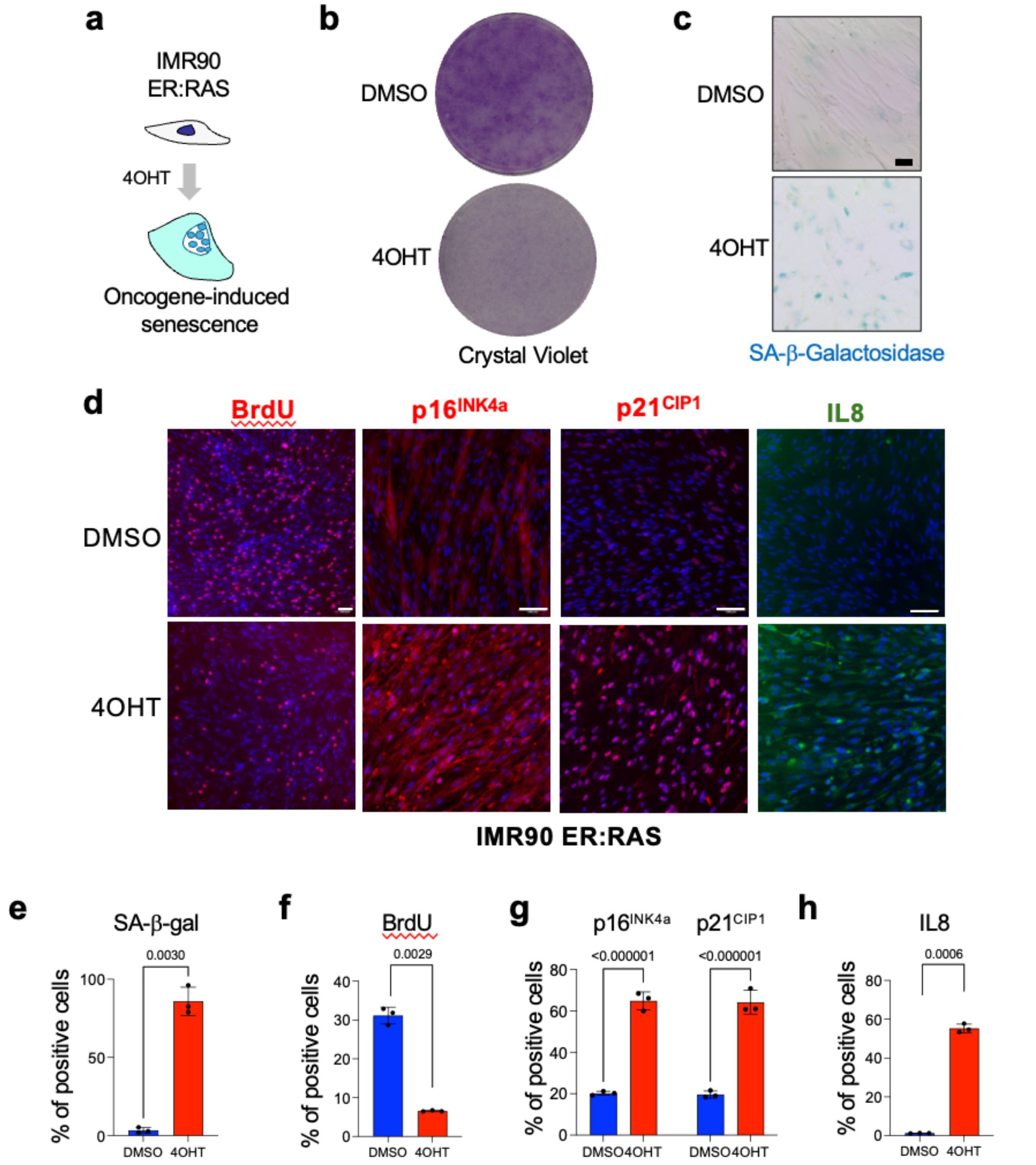

Supplementary Figure 1. IMR90 ER:RAS as a model of oncogene-induced senescence. **a,** Schematic of senescence induction in IMR90 ER:RAS cells. **b,** Crystal violet staining of DMSO and 4OHT-treated IMR90 ER:RAS cells. **c,** SA-β-gal staining images of DMSO and 4OHT-treated IMR90 ER:RAS cells. Scale bar 50 μm. **d,** Representative immunofluorescence (IF) images of BRDU, p16^{INK4a}, p21^{CIP1,} and IL8 in IMR90 ER:RAS cells after 7 days of DMSO or 4OHT treatment. Scale bar, 100 μm. **e,** Quantification of SA-β-gal staining. Data represent mean ± SD (n = 3). Statistical test used: unpaired t-test (two-tailed). **f,** Quantification of BrdU incorporation by IF staining in IMR90 ER:RAS cells. Data represent mean ± SD (n = 3). Statistical test used: paired t-test (two-tailed). **g,** Quantification of p16^{INK4a} and p21^{CIP1} IF staining. Data represent mean ± SD (n = 3). Statistical test used: two-way ANOVA (Šídák's multiple comparisons test). **h,** Quantification of IL8 IF staining. Data represent mean ± SD (n = 3). Statistical test used: paired t-test (two-tailed).

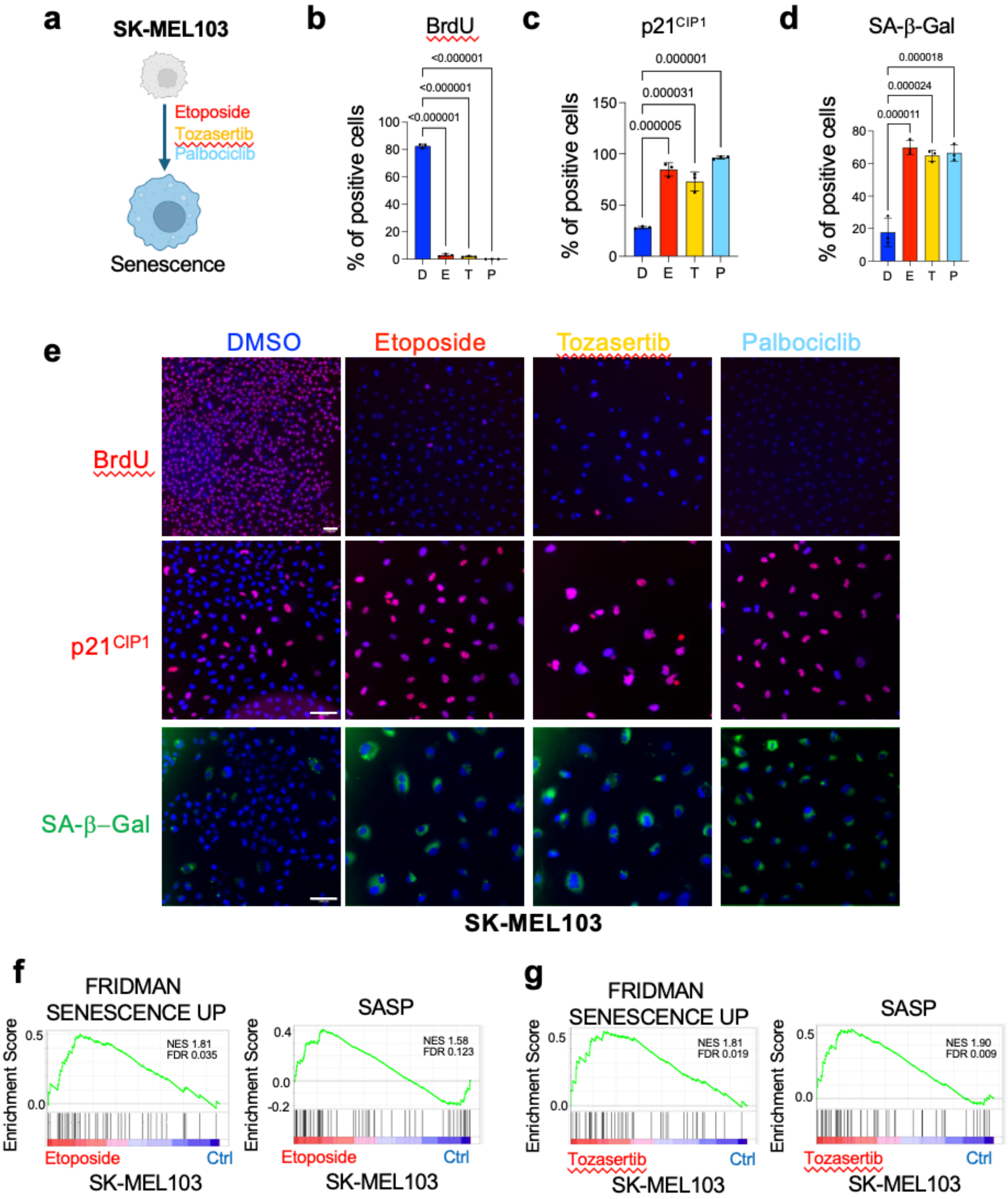

Supplementary Figure 2. SK-MEL103 as a system of therapy-induced senescence. a, Schematic of senescence induction in SK-MEL103 cells. Created in BioRender. D'Ambrosio, M. (2025) https://BioRender.com/3yd2zb9 **b,** Quantification of BrdU incorporation by IF staining in SK-MEL103 cells. Data represent mean ± SD (n = 3). Statistical test used: one-way ANOVA (Dunnett's multiple comparisons test). **c,** Quantification of p21^{CIP1} IF staining in SK-MEL103 cells. Data represent mean ± SD (n = 3). Statistical test used: one-way ANOVA (Dunnett's multiple comparisons test). **d,** Quantification of SA-β-gal staining in SK-MEL103 cells. Data represent mean ± SD (n = 3). Statistical test used: one-way ANOVA (Dunnett's multiple comparisons test). **e,** Representative IF images of BRDU, p21^{CIP1,} and SA-β-gal in SK-MEL103 cells after 6 days of treatment with DMSO, Etoposide, Tozasertib, or Palbociclib. Scale bar, 100 μm. **f-g,** GSEA enrichment plots of Senescence and SASP signature in DMSO vs Etoposide (**f**) and DMSO vs Tozasertib (**g**) in SK-MEL103 cells.

a

Library name	Catalog ID	Name on this study	Related probe 1	Related probe 2
CLA01-P20	Z56926666	SCLA1	SCLA1a	SCLA1b
CLA02-O17	Z203045204	SCLA2	SCLA2a	SCLA2b
CLA04-G12	Z1562151884	SCLA3	SCLA3a	SCLA3b
CLA03-M11	Z57046997	SCLA4	SCLA4a	SCLA4b

b

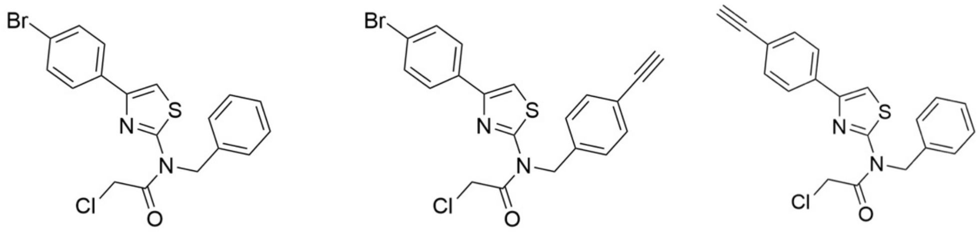

SCLA1 **SCLA1a** **SCLA1b**

c

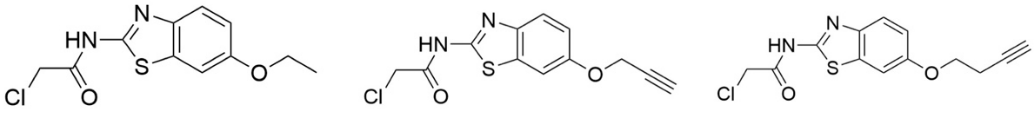

SCLA2 **SCLA2a** **SCLA2b**

d

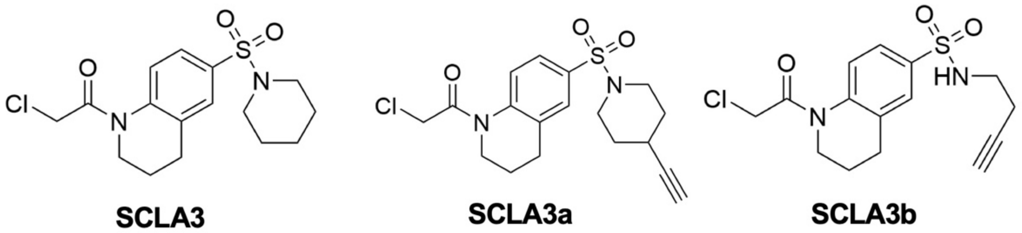

SCLA3 **SCLA3a** **SCLA3b**

e

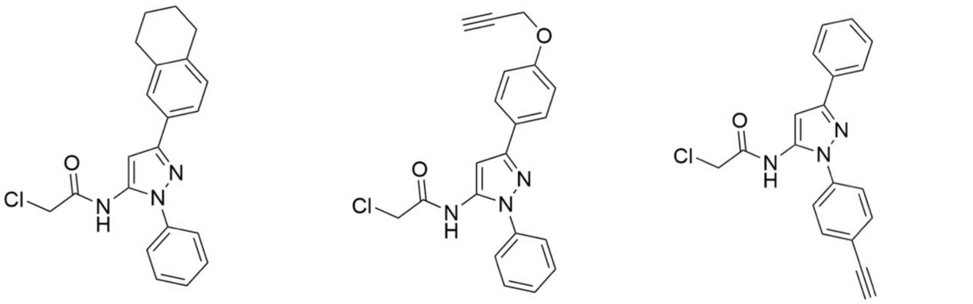

SCLA4 **SCLA4a** **SCLA4b**

Supplementary Figure 3. Chemical structures of senolytic chloroacetamides (SCLAs) and their alkynylated derivatives. a, Table listing the four selected chloroacetamides and their corresponding alkynylated probes. **b-e,** Chemical structures of the four senescence chloroacetamides and their respective alkynylated probes.

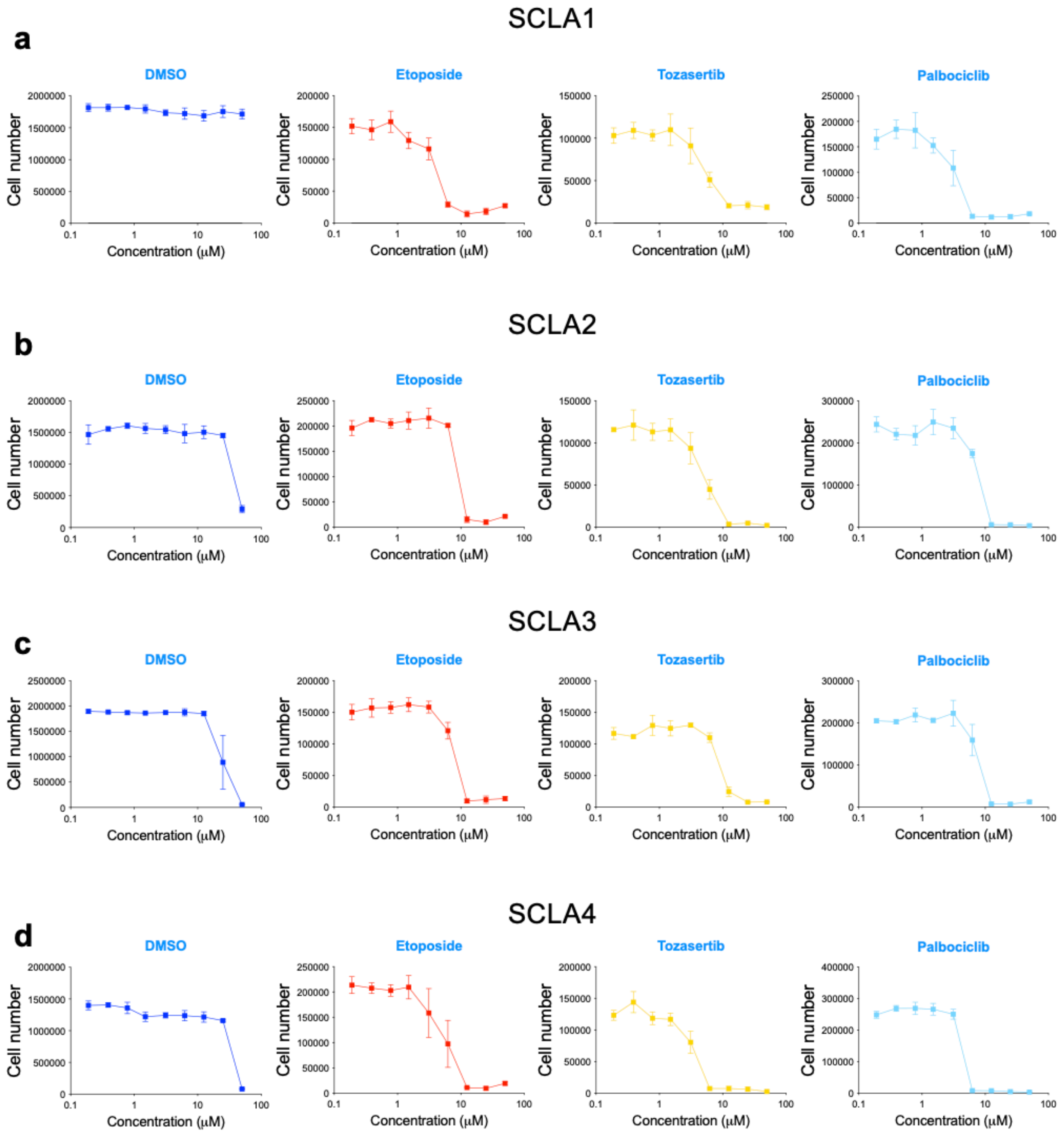

Supplementary Figure 4. Dose-response curve based on cell numbers. a-d, Dose-response curves based on cell numbers of SCLA1 (**a**), SCLA2 (**b**), SCLA3 (**c**) and SCLA4 (**d**) in SK-MEL103 cells treated with DMSO, Etoposide, Tozasertib, or Palbociclib for 6 days. Data represent mean ± SD.

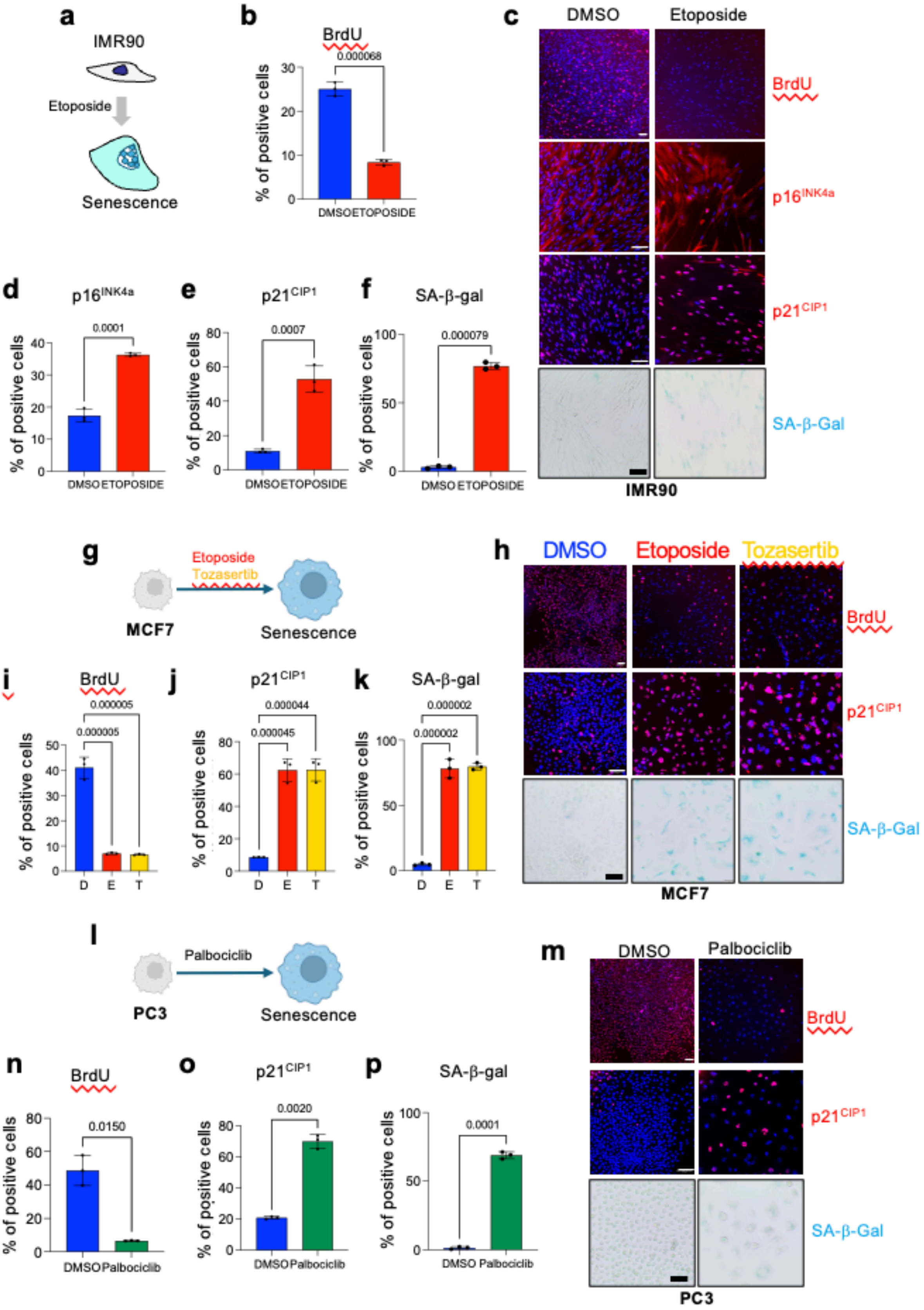

Supplementary Figure 5. Therapy-induced senescence in IMR90, MCF7, and PC3 cells. a, Schematic of senescence induction in IMR90 cells. **b,** Quantification of BrdU incorporation by IF staining in IMR90 cells treated with DMSO or Etoposide. Data represent mean ± SD (n = 3). Statistical test used: unpaired t-test (two-tailed). **c,** Representative images of BRDU,

$p16^{INK4a}$, $p21^{CIP1,}$ and SA-β-gal in IMR90 cells after treatment with DMSO or Etoposide. Scale bar, 100 μm for IF; 50 μm for SA-β-gal. **d,** Quantification of $p16^{INK4a}$ IF staining in IMR90 cells. Data represent mean ± SD (n = 3). Statistical test used: unpaired t-test. **e,** Quantification of $p21^{CIP1}$ IF staining in IMR90 cells. Data represent mean ± SD (n = 3). Statistical test used: unpaired t-test (two-tailed). **f,** Quantification of SA-β-gal staining. Data represent mean ± SD (n = 3). Statistical test used: unpaired t-test (two-tailed). **g,** Schematic of senescence induction in MCF7 cells. Created in BioRender. D'Ambrosio, M. (2025) https://BioRender.com/3yd2zb9 **h,** Representative images of BrdU, $p21^{CIP1,}$ and SA-β-gal in MCF7 cells after 6 days of treatment with DMSO, Etoposide, or Tozasertib. Scale bar, 100 μm for IF; 50 μm for SA-β-gal. **i,** Quantification of BrdU incorporation by IF staining in MCF7 cells. Data represent mean ± SD (n = 3). Statistical test used: one-way ANOVA (Dunnett's multiple comparisons test). **j,** Quantification of $p21^{CIP1}$ IF staining in MCF7 cells. Data represent mean ± SD (n = 3). Statistical test used: one-way ANOVA (Dunnett's multiple comparisons test). **k**, Quantification of SA-β-gal staining. Data represent mean ± SD (n = 3). Statistical test used: one-way ANOVA (Šídák's multiple comparisons test). **l,** Schematic of senescence induction in PC3 cells. Created in BioRender. D'Ambrosio, M. (2025) https://BioRender.com/3yd2zb9 **m,** Representative images of BRDU, $p21^{CIP1,}$ and SA-β-gal in PC3 cells after 6 days of treatment with DMSO or Palbociclib. Scale bar, 100 μm for IF; 50 μm for SA-β-gal. **n,** Quantification of BrdU incorporation by IF staining in PC3 cells. Data represent mean ± SD (n = 3). Statistical test used: unpaired t-test (two-tailed). **o,** Quantification of $p21^{CIP1}$ IF staining in PC3 cells. Data represent mean ± SD (n = 3). Statistical test used: unpaired t-test (two-tailed). **p,** Quantification of SA-β-gal staining. Data represent mean ± SD (n = 3). Statistical test used: unpaired t-test (two-tailed).

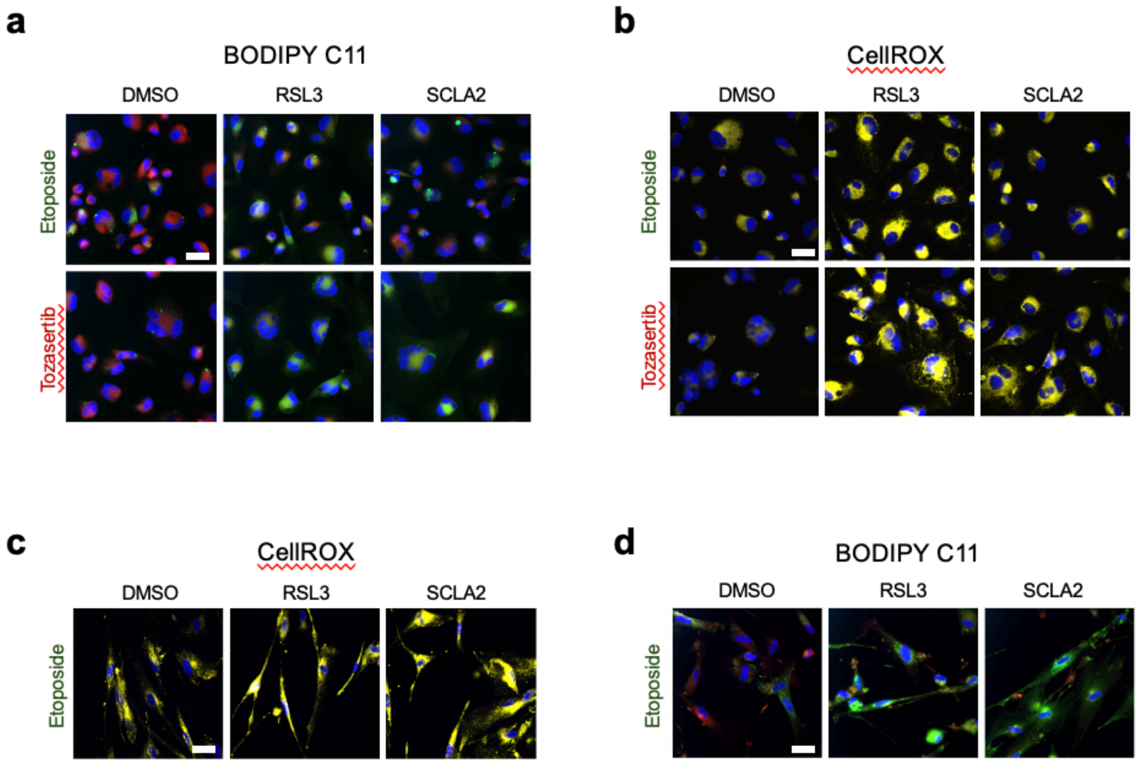

Supplementary Figure 6. Bodipy C11 and CellROX staining after RSL3 and SCLA2 treatment. a, Representative images of BODIPY™ 581/591 C11 in senescent SK-MEL103 cells after treatment with RSL3 and SCLA2. Scale bar, 50 μm. **b,** Representative images of CellROX in senescent SK-MEL103 cells after treatment with RSL3 and SCLA2. Scale bar, 50 μm. **c,** Representative images of CellROX in senescent IMR90 cells after treatment with RSL3 and SCLA2. Scale bar, 50 μm. **d,** Representative images of BODIPY™ 581/591 C11 in senescent IMR90 cells after treatment with RSL3 and SCLA2. Scale bar, 50 μm.
