# Supplementary material for: Electrophilic compound screening identifies GPX4-dependent ferroptosis as a senescence vulnerability
Source: Nat Cell Biol. 2026 Apr 24;28(5):915–29. doi: 10.1038/s41556-026-01921-z (PMC13179136; doi:10.1038/s41556-026-01921-z)
Supplement: Supplementary file 2 — Reporting Summary [file 41556_2026_1921_MOESM2_ESM.pdf]

nature portfolio

Corresponding author(s): Jesus Gil

Last updated by author(s): Feb 23, 2026

Reporting Summary

Nature Portfolio wishes to improve the reproducibility of the work that we publish. This form provides structure for consistency and transparency in reporting. For further information on Nature Portfolio policies, see our Editorial Policies and the Editorial Policy Checklist.

Statistics

For all statistical analyses, confirm that the following items are present in the figure legend, table legend, main text, or Methods section.

n/a	Confirmed	
☐	☒	The exact sample size (*n*) for each experimental group/condition, given as a discrete number and unit of measurement
☐	☒	A statement on whether measurements were taken from distinct samples or whether the same sample was measured repeatedly
☐	☒	The statistical test(s) used AND whether they are one- or two-sided<br>*Only common tests should be described solely by name; describe more complex techniques in the Methods section.*
☒	☐	A description of all covariates tested
☒	☐	A description of any assumptions or corrections, such as tests of normality and adjustment for multiple comparisons
☐	☒	A full description of the statistical parameters including central tendency (e.g. means) or other basic estimates (e.g. regression coefficient) AND variation (e.g. standard deviation) or associated estimates of uncertainty (e.g. confidence intervals)
☐	☒	For null hypothesis testing, the test statistic (e.g. *F*, *t*, *r*) with confidence intervals, effect sizes, degrees of freedom and *P* value noted<br>*Give P values as exact values whenever suitable.*
☒	☐	For Bayesian analysis, information on the choice of priors and Markov chain Monte Carlo settings
☒	☐	For hierarchical and complex designs, identification of the appropriate level for tests and full reporting of outcomes
☒	☐	Estimates of effect sizes (e.g. Cohen's *d*, Pearson's *r*), indicating how they were calculated

Our web collection on statistics for biologists contains articles on many of the points above.

Software and code

Policy information about availability of computer code

Data collection	IN Cell Analyzer 2000 version 5-2-14311 (64-bit) GE Healthcare<br>Microsoft Excel for Office 365 MSO (16.01.12527.21378) 63-bit
Data analysis	GraphPad Prism 10 . IN Cell Investigator 1000 workstation 3.7.2, build 1860. IN Carta v1.17.0412545 software (Molecular Devices). ImageJ 2.0.0-rc-54/1.53c. HTSeq v0.5.3p9. DESeq2_1.48.2 GSEA v4.10 (Broad Institute). R

For manuscripts utilizing custom algorithms or software that are central to the research but not yet described in published literature, software must be made available to editors and reviewers. We strongly encourage code deposition in a community repository (e.g. GitHub). See the Nature Portfolio guidelines for submitting code & software for further information.

Data

Policy information about availability of data

All manuscripts must include a data availability statement. This statement should provide the following information, where applicable:

- Accession codes, unique identifiers, or web links for publicly available datasets
- A description of any restrictions on data availability
- For clinical datasets or third party data, please ensure that the statement adheres to our policy

Source numerical data and unprocessed blots are available as source data. RNA-seq data have been deposited in the GEO database: GSE307575 (private token: ibspwceuxbgxvel) and GSE313741 (private token: etapegcsrhipzar). The screen data and retesting data are provided in Tables S1 and S2, respectively. The mass

spectroscopy data are provided in Table S4 and are available via ProteomeXchange with identifiers PXD068417 (token: pcV2Wo3s1Hc2) and PXD068572 (token: ViaDHeZTIpv5). The lipidomics data are provided in Table S5 and have been deposited in MassIVE under reference: MSV000099167 (password: Ferr_lipids; https://doi.org/doi:10.25345/C5V11VZ97).

Research involving human participants, their data, or biological material

Policy information about studies with human participants or human data. See also policy information about sex, gender (identity/presentation), and sexual orientation and race, ethnicity and racism.

Reporting on sex and gender	n/a
Reporting on race, ethnicity, or other socially relevant groupings	n/a
Population characteristics	*Describe the covariate-relevant population characteristics of the human research participants (e.g. age, genotypic information, past and current diagnosis and treatment categories). If you filled out the behavioural & social sciences study design questions and have nothing to add here, write "See above."*
Recruitment	n/a
Ethics oversight	n/a

Note that full information on the approval of the study protocol must also be provided in the manuscript.

Field-specific reporting

Please select the one below that is the best fit for your research. If you are not sure, read the appropriate sections before making your selection.

☒ Life sciences ☐ Behavioural & social sciences ☐ Ecological, evolutionary & environmental sciences

For a reference copy of the document with all sections, see nature.com/documents/nr-reporting-summary-flat.pdf

Life sciences study design

All studies must disclose on these points even when the disclosure is negative.

Sample size	No statistical methods were used to pre-determine sample size, but our sample size are similar to those reported previously.
Data exclusions	No data exclusions
Replication	Every figure legend mentions how many times each experiment was performed.
Randomization	For the ID8 studies, mice were randomized in the different experimental groups 21 days after cells injections. The tumors are intraperitoneal injected, so the randomization is blind. For PC3 subcoutaneus model, mice were randomized when the tumors reached a volume of approximately 100 mm3. For SK-MEL103 subcoutaneus model, mice were randomized when the tumors reached a volume of approximately 50 mm.<br>For cell culture experiment, there was no randomization because the treatment groups were always compared to DMSO- treated controls.
Blinding	Investigators were not blinded during the cell culture or in vivo experiments, but whenever possible (e.g., quantitative IF and IHC analysis), analysis was performed using automated procedures.

Reporting for specific materials, systems and methods

We require information from authors about some types of materials, experimental systems and methods used in many studies. Here, indicate whether each material, system or method listed is relevant to your study. If you are not sure if a list item applies to your research, read the appropriate section before selecting a response.

Materials & experimental systems

n/a	Involved in the study
☐	☒ Antibodies
☐	☒ Eukaryotic cell lines
☒	☐ Palaeontology and archaeology
☐	☒ Animals and other organisms
☒	☐ Clinical data
☒	☐ Dual use research of concern
☒	☐ Plants

Methods

n/a	Involved in the study
☒	☐ ChIP-seq
☒	☐ Flow cytometry
☒	☐ MRI-based neuroimaging

Antibodies

Antibodies used

The following primary antibodies were used in this study: GPX4 (EPNCIR144, Rb, Abcam, ab125066, lot 1078033-048), 1:500 for IF and 1:1000 for WB; p21 (EPR18021 , Rb, Abcam, ab188224, lot 1013656-10), 1:500; BRDU (3D4 , Mouse, BD Biosciences, BD 555627, lot 2301623), 1:2000; p16 (JC8,CRUK), 1:1000; p21 (12D1, Rb, CST 2947S, lot 15) 1:2000 for IF and 1:400 for IHC; IL8 (6217 ,Mouse, R&D MAB-208, lot ASJ3822071), 1;250; GAPDH (Rb, Sigma G9545, lot 0000-241509) 1:2000; p27 (Y236, Rb, Abcam, ab32034), 1:2000; N-RAS (F155, Mouse, Santa Cruz Technology,MABC1152), 1:500; FTH1 (D1D4, Rb, CST 4393S, lot 6), 1:1000; FTL (F4T8H, Rb, CST 68106, lot 1), 1:1000; CD45 (EPR20033, Rb, Abcam, ab208022, lot 1089036-3) 1:5000; WT1 (CAN-R9-56-2, Rb, Abcam, ab89901. lot 1092014-48), 1:500.. The following secondary antibodies were used in this study: goat anti-rabbit AF544 (Invitrogen, A11037, lot 2841610); goat anti-mouse AF594 (invitrogen, A11032, lot 3071410); rabbit anti-mouse AF488 (Invitrogen, A11059, lot 2261403); Rabbit igG (CST, 8114S, lot 30); Mouse igG (CST, 8124S, lot 32); anti-rabbit IgG -HRP conjugated (Donkey, Abcam, A16035, 1:5000); anti-mouse IgG HRP-conjugated(Donkey, Abcam, A16011, 1:5000)

Validation

Validation has been described in previous papers or manufacturer's website

Eukaryotic cell lines

Policy information about cell lines and Sex and Gender in Research

Cell line source(s)

IMR90 (human, ATCC CCL-186), SK-MEL103 (ATCC, HTB-69), MCF7 (ATCC, HTB-22), and PC3 (ATCC, CRL-1435)

Authentication

Describe the authentication procedures for each cell line used OR declare that none of the cell lines used were authenticated.

Mycoplasma contamination

All cells were routinely tested for mycoplasma contamination and were negative.

Commonly misidentified lines
(See ICLAC register)

none

Animals and other research organisms

Policy information about studies involving animals; ARRIVE guidelines recommended for reporting animal research, and Sex and Gender in Research

Laboratory animals

Ovarian cancer experiments were performed in C57BL/6J female mice (6-7 weeks old).
Prostate cancer experiments were performed in NRG male mice (12-weeks old).
Melanoma experiments were performed in NRG female mice (12-weeks old)
HDTVI experiments were performed in C57BL/6J male mice (8 weeks old).

Wild animals

n/a

Reporting on sex

Sex was related to the type of cancer cells used.

Field-collected samples

n/a

Ethics oversight

This research complied with all relevant ethical regulations and was approved and overseen by the following ethics review boards. Experiments performed in the ovarian cancer model were approved by the animal welfare and ethics review body (AWERB) at Imperial College London. All experiments conformed to UK Home Office regulations under the Animals (Scientific Procedures) Act 1986, including Amendment Regulations 2012, and adhered to ARRIVE guidelines. Liver cancer initiation experiments were performed under project licence number PPL 70/09080. Ovarian cancer experiments were performed under the project licence number PP1321516. Prostate cancer and melanoma cancer experiments in mice were carried out in the BIOS+ animal facility under specific pathogen-free (SPF) conditions, with approval from project licenses 35293, 36369, and 37480.The hydrodynamic tail vein injection experiment with RSL3 was carried out according to German law and with the approval of the Regierungspräsidium Karlsruhe (G-213/20).

Note that full information on the approval of the study protocol must also be provided in the manuscript.

Plants

Seed stocks	n/a
Novel plant genotypes	n/a
Authentication	n/a
