# Supplementary material for: Electrophilic compound screening identifies GPX4-dependent ferroptosis as a senescence vulnerability
Source: Nat Cell Biol. 2026 Apr 24;28(5):915–29. doi: 10.1038/s41556-026-01921-z (PMC13179136; doi:10.1038/s41556-026-01921-z)

Synthesis of probes

Probes SCLA1a and SCLA1b

Based on the structure of **SCLA1**, two probes were designed by adding the alkyne group in the 4-unsubstituted position of the benzyl group **(SCLA1a)** or by replacing the bromo-substituent on the phenyl ring **(SCLA1b)**.

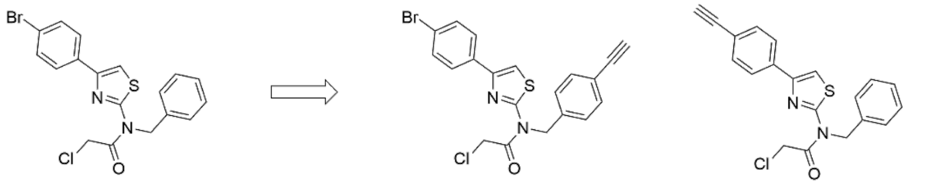

SCLA1 **SCLA1a** **SCLA1b**

Scheme 1. Synthesis of probe SCLA1a.

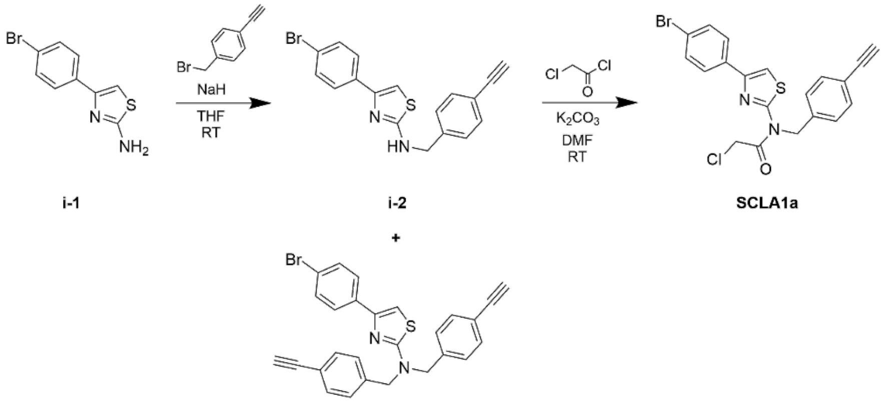

i-1 **i-2** **SCLA1a** **i-3**

<u>4-(4-bromophenyl)-*N*-(4-ethynylbenzyl)thiazol-2-amine (i-2) and 4-(4-bromophenyl)-*N,N*-bis(4-ethynylbenzyl)thiazol-2-amine (i-3)</u>

To a solution of 4-(4-bromophenyl)thiazol-2-amine (200 mg, 0.78 mmol) in anhydrous THF (5 mL) under nitrogen, was added sodium hydride (31 mg, 0.78 mmol, 60%) at 0 °C. After 15 min, 1-(bromomethyl)-4-ethynylbenzene (153 mg, 0.76 mmol) was added and the reaction stirred at room temperature for 24 h. The mixture was concentrated, water was added to the residue, and after extraction with ethyl acetate (x3), the combined organic layers were washed with brine, dried over sodium sulfate, and concentrated under reduced pressure. Purification with flash column chromatography (5-20% ethyl acetate in hexane) provided the two products (mono and di-substituted):

4-(4-bromophenyl)-*N*-(4-ethynylbenzyl)thiazol-2-amine (33 mg, yield 11%) as a yellow solid. R_f: 0.5 (hexane/ethyl acetate: 8/2). ^{1}H NMR (400 MHz, $CDCl_3$) δ 7.68 – 7.60 (m, 2H), 7.50 – 7.39 (m, 4H), 7.30 (d, *J* = 8.1 Hz, 2H), 6.68 (s, 1H), 6.22 (s, 1H), 4.48 (s, 2H), 3.09 (s, 1H). ^{13}C NMR (101 MHz, $CDCl_3$) δ 169.5, 150.4, 138.5, 133.8, 132.6, 131.8, 127.7, 127.6, 121.7, 121.6, 101.7, 83.4, 77.6, 49.6. ES(-) *m/z* 368.8 [M-H]$^-$.

4-(4-bromophenyl)-*N,N*-bis(4-ethynylbenzyl)thiazol-2-amine (55 mg, yield 15%) as a yellow solid. R_f: 0.7 (hexane/ ethyl acetate: 8/2). ^{1}H NMR (400 MHz, $CDCl_3$) δ 7.79 – 7.72 (m, 2H), 7.55 – 7.50 (m, 2H), 7.49 (d, *J* = 8.1 Hz, 4H), 7.26 (d, *J* = 8.0 Hz, 4H), 6.76 (s, 1H), 4.71 (s, 4H), 3.12 (s, 2H). ^{13}C NMR (101 MHz, $CDCl_3$) δ 170.6, 150.7, 137.4, 134.0, 132.6, 131.7, 127.9, 127.8, 121.6, 101.7, 83.4, 53.7.

<u>*N*-(4-(4-bromophenyl)thiazol-2-yl)-2-chloro-*N*-(4-ethynylbenzyl)acetamide (SCLA1a)</u>

To a solution of 4-(4-bromophenyl)-*N*-(4-ethynylbenzyl)thiazol-2-amine (30 mg, 0.081 mmol) in anhydrous DMF (2 mL) under nitrogen, potassium carbonate (22 mg, 0.16 mmol) and chloroacetyl chloride (9.7 µL, 0.12 mmol) were added at 0 °C and the reaction stirred at room temperature for 24 h. Then, chloroacetyl chloride (9.7 µL, 0.12 mmol) was added again, and the reaction was stirred for another 24 h. Saturated ammonium chloride solution was added to the reaction mixture, and after extraction with ethyl acetate (x3), the combined organic layers were washed with brine, dried over sodium sulfate, and concentrated under reduced pressure. Purification with flash column chromatography (5-30% ethyl acetate in hexane) afforded the title compound (23 mg, yield 64%) as a white solid. R_f: 0.5 (hexane/ethyl acetate: 8/2). ^{1}H NMR (400 MHz, $CDCl_3$) δ 7.71 – 7.63 (m, 2H), 7.49 (dd, *J* = 8.3, 6.1 Hz, 4H), 7.28 (s, 1H), 7.23 (d, *J* = 8.0 Hz, 2H), 5.68 (s, 2H), 4.24 (s, 2H), 3.09 (s, 1H). ^{13}C NMR (126 MHz, $CDCl_3$) δ 166.3, 159.3, 148.4, 136.8, 133.3, 133.0, 131.9, 127.7, 126.4, 122.2, 110.5, 83.0, 78.1, 50.6, 42.0. HRMS (ES+) *m/z* calc. for $C_{20}H_{15}BrClN_2OS$ [M+H]$^+$: 446.9757, found 446.9756.

Scheme 2. Synthesis of probe SCLA1b.

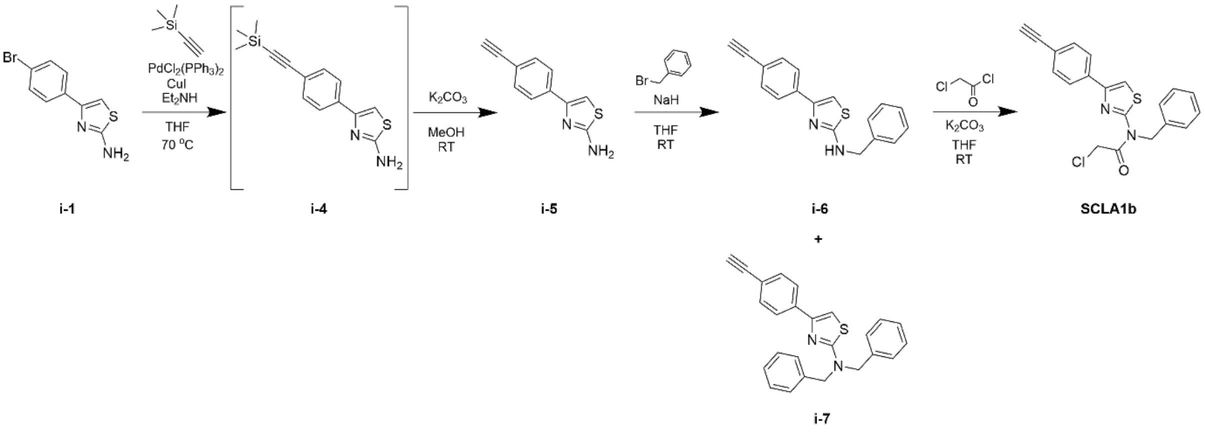

<u>4-(4-ethynylphenyl)thiazol-2-amine (i-5)</u>

To a solution of 4-(4-bromophenyl)thiazol-2-amine (1000 mg, 3.92 mmol) in anhydrous tetrahydrofuran (10 mL), evacuated and purged with nitrogen, were added diethylamine (2.03 mL, 19.6 mmol), (trimethylsilyl)acetylene (1.63 mL, 11.8 mmol), copper(I) iodide (149 mg, 0.78 mmol) and bis(triphenylphosphine)palladium(II) dichloride (275 mg, 0.39 mmol) and the reaction was heated to 70 °C for 24 h. The reaction mixture was filtered through a pad of celite, and the filtrate was concentrated under reduced pressure. To form the deprotected product, the residue was dissolved in methanol (30 mL), and potassium carbonate (2.71 g, 19.6 mmol) was added. The reaction was stirred at room temperature for 1 h. Then, the mixture was concentrated, water was added to the residue, and after extraction with ethyl acetate (x3), the combined organic layers were washed with brine, dried over sodium sulfate, and concentrated under reduced pressure. Purification with flash column chromatography (10-50% ethyl acetate in hexane) afforded the title compound (320 mg, yield 41%) as a brown solid. R_f: 0.5 (hexane/ethyl acetate: 1/1). ^{1}H NMR (400 MHz, DMSO-d_6) δ 7.82 – 7.77 (m, 2H), 7.49 – 7.44 (m, 2H), 7.12 (s, 2H), 7.11 (s, 1H), 4.21 (s, 1H). ^{13}C NMR (101 MHz, DMSO-d_6) δ 168.3, 148.9, 135.2, 131.9, 125.6, 120.2, 120.1, 103.1, 83.6, 81.2. ES(+) *m/z* 200.8 $[M+H]^+$.

***N*-benzyl-4-(4-ethynylphenyl)thiazol-2-amine (i-6) and *N,N*-dibenzyl-4-(4-ethynylphenyl)thiazol-2-amine (i-7)**

To a solution of 4-(4-ethynylphenyl)thiazol-2-amine (200 mg, 1.00 mmol) in anhydrous THF (8 mL) under nitrogen, was added sodium hydride (40 mg, 1.0 mmol, 60%) at 0 °C. After 15 min, benzyl bromide (119 µL, 1.00 mmol) was added, and the reaction was stirred at room temperature for 18 h. The mixture was concentrated, saturated ammonium chloride solution was added to the residue, and after extraction with ethyl acetate (x3), the combined organic layers were washed with brine, dried over sodium sulfate, and concentrated under reduced pressure. Purification with flash column chromatography (5-50% ethyl acetate in hexane) provided the two products (mono and di-substituted):

N-benzyl-4-(4-ethynylphenyl)thiazol-2-amine (35 mg, yield 12%) as a yellow solid. R_f: 0.5 (hexane/ethyl acetate 8/2). ^{1}H NMR (400 MHz, $CDCl_3$) δ 7.76 (d, *J* = 8.0 Hz, 2H), 7.48 (d, *J* = 8.0 Hz, 2H), 7.39 – 7.30 (m, 6H), 6.74 (s, 1H), 5.93 (s, 1H), 4.50 (d, *J* = 4.3 Hz, 2H), 3.12 (s, 1H). ^{13}C NMR (101 MHz, $CDCl_3$) δ 169.5, 150.7, 137.7, 135.3, 132.5, 128.9, 127.9, 127.8, 126.0, 121.1, 102.3, 83.9, 77.8, 49.9. ES(+) *m/z* 291.1 $[M+H]^+$.

N,N-dibenzyl-4-(4-ethynylphenyl)thiazol-2-amine (55 mg, yield 14%) as a yellow solid. R_f: 0.8 (hexane/ethyl acetate 8/2). ^{1}H NMR (400 MHz, $CDCl_3$) δ 7.88 – 7.82 (m, 2H), 7.54 – 7.48 (m, 2H), 7.37 – 7.27 (m, 10H), 6.77 (s, 1H), 4.72 (s, 4H), 3.13 (s, 1H). ^{13}C NMR (101 MHz, $CDCl_3$) δ 170.9, 150.9, 136.8, 135.6, 132.5, 128.8, 128.1, 127.7, 126.0, 121.0, 102.0, 84.1, 77.7, 53.7. ES(+) *m/z* 381.1 $[M+H]^+$.

***N*-benzyl-2-chloro-*N*-(4-(4-ethynylphenyl)thiazol-2-yl)acetamide (SCLA1b)**

To a solution of *N*-benzyl-4-(4-ethynylphenyl)thiazol-2-amine (30 mg, 0.10 mmol) in anhydrous DMF (2 mL) under nitrogen, potassium carbonate (43 mg, 0.31 mmol) and chloroacetyl chloride (25 µL, 0.31 mmol) were added at 0 °C and the reaction stirred at room temperature for 24 h. Saturated ammonium chloride solution was added to the reaction mixture, and after extraction with ethyl acetate (x3), the combined organic layers were washed with brine, dried over sodium sulfate, and concentrated under reduced pressure. Purification with flash column chromatography (5-30% ethyl acetate in hexane) afforded the title compound (12 mg, yield 32%) as a white solid. R_f: 0.5 (hexane/ethyl acetate: 8/2). ^{1}H NMR (400 MHz, $CDCl_3$) δ 7.81 –

7.77 (m, 2H), 7.52 – 7.48 (m, 2H), 7.39 – 7.26 (m, 6H), 5.70 (s, 2H), 4.27 (s, 2H), 3.13 (s, 1H). ^{13}C NMR (101 MHz, $CDCl_3$) δ 166.4, 136.1, 134.7, 132.6, 129.3, 128.2, 126.4, 126.0, 121.6, 111.0, 83.7, 78.1, 50.7, 42.3. HRMS (ES+) *m/z* calc. for $C_{20}H_{16}ClN_2OS$ $[M+H]^+$: 367.0672, found 367.0662.

Probes SCLA2a and SCLA2b

Based on the structure of **SCLA2**, two probes were designed by adding the alkyne in the place of the ethoxy-group using chains with one **(SCLA2a)** or two **(SCLA2b)** methylene groups.

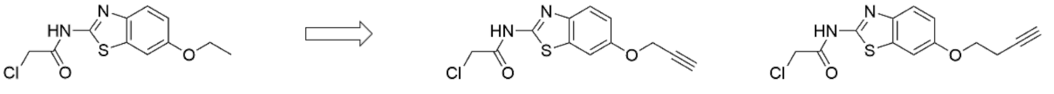

Scheme 3. Synthesis of probe SCLA2a.

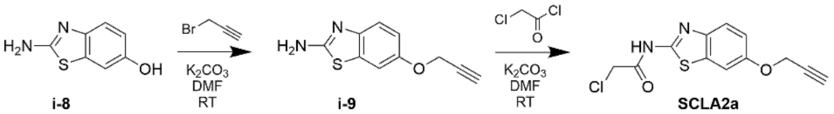

<u>6-(prop-2-yn-1-yloxy)benzo[*d*]thiazol-2-amine (i-9)</u>

To a solution of 2-amino-6-hydroxybenzothiazole (200 mg, 1.20 mmol) in anhydrous DMF (4 mL) under nitrogen, was added potassium carbonate (333 mg, 2.41 mmol) and propargyl bromide 80% w/w in toluene (141 µL, 1.26 mmol), and the reaction was stirred at room temperature for 18 h. Saturated ammonium chloride solution was added to the reaction mixture, and after extraction with ethyl acetate (x3), the combined organic layers were washed with brine, dried over sodium sulfate, and concentrated under reduced pressure. Purification with flash column chromatography (20-100% ethyl acetate in hexane) afforded the title compound (145 mg, yield 59%) as a light brown solid. R_f: 0.5 (hexane/ethyl acetate: 1/2). ^{1}H NMR (400 MHz, DMSO-d_6) δ 7.34 (d, J = 2.7 Hz, 1H), 7.28 (s, 2H), 7.24 (d, J = 8.7 Hz, 1H), 6.86 (dd, J = 8.7, 2.6 Hz, 1H), 4.75 (d, J = 2.4 Hz, 2H), 3.55 (t, J = 2.4 Hz, 1H). ^{13}C NMR (101 MHz, DMSO-d_6) δ 165.1, 152.1, 147.5, 131.8, 118.0, 113.8, 107.1, 79.6, 78.1, 56.1. *NOE peaks of CH_2 with Ar-H confirm that the -OH was alkylated.* ES(+) *m/z* 205.1 $[M+H]^+$. *Synthesized according to* [23].

<u>2-chloro-*N*-(6-(prop-2-yn-1-yloxy)benzo[*d*]thiazol-2-yl)acetamide (SCLA2a)</u>

To a solution of 6-(prop-2-yn-1-yloxy)benzo[*d*]thiazol-2-amine (70 mg, 0.34 mmol) in anhydrous DMF (2 mL) under nitrogen at 0 °C, was added potassium carbonate (95 mg, 0.69 mmol) and chloroacetyl chloride (41 µL, 0.51 mmol) and the reaction stirred at room temperature for 4 h. Saturated ammonium chloride solution was added to the reaction mixture, and after extraction with ethyl acetate (x3), the combined organic layers were washed with brine, dried over sodium sulfate and concentrated under reduced pressure. Purification with flash column chromatography (5-100% ethyl acetate in hexane) afforded the title compound (60 mg, yield 62%) as a white solid. R_f: 0.5 (hexane/ethyl acetate: 1/1). ^{1}H NMR (400 MHz, DMSO-d_6) δ 12.64 (s, 1H), 7.69 (d, J = 8.9 Hz, 1H), 7.64 (d, J = 2.6 Hz, 1H), 7.10 (dd, J = 8.9,

2.7 Hz, 1H), 4.85 (d, *J* = 2.5 Hz, 2H), 4.45 (s, 2H), 3.59 (s, 1H). ^{13}C NMR (101 MHz, DMSO-*d*$_6$) δ 165.7, 155.9, 154.1, 143.1, 132.6, 121.4, 115.6, 106.5, 79.2, 78.4, 56.1, 42.5. ES(-) *m/z* 278.9 $[M-H]^-$.

Scheme 4. Synthesis of probe SCLA2b.

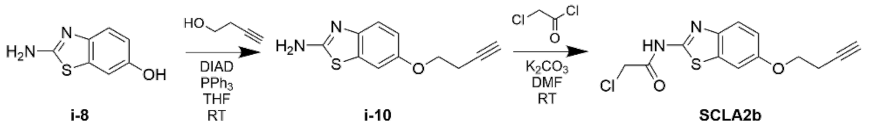

<u>6-(but-3-yn-1-yloxy)benzo[*d*]thiazol-2-amine (i-10)</u>

To a solution of 2-aminobenzo[*d*]thiazol-6-ol (100 mg, 0.60 mmol) in dry THF (5 mL) under nitrogen, was added but-3-yn-1-ol (68 µL, 0.90 mmol), diisopropyl azodicarboxylate (118 µL, 0.60 mmol), and triphenylphosphine (158 mg, 0.60 mmol), and the reaction was stirred at room temperature for 24 h. The reaction mixture was concentrated, the residue was dissolved in ethyl acetate, washed with NaOH 1M (x2) and brine, and the organic phase was dried over sodium sulfate and concentrated under reduced pressure. Purification with flash column chromatography (0-10% methanol in dichloromethane) afforded the title compound (8.0 mg, yield 6%) as a brown solid. R_f: 0.4 (hexane/ethyl acetate: 1/1). ^{1}H NMR (400 MHz, $CDCl_3$) δ 7.44 (d, *J* = 8.8 Hz, 1H), 7.14 (d, *J* = 2.6 Hz, 1H), 6.92 (dd, *J* = 8.8, 2.6 Hz, 1H), 5.49 – 5.09 (br, 2H), 4.10 (t, *J* = 7.0 Hz, 2H), 2.68 (td, *J* = 7.0, 2.7 Hz, 2H), 2.05 (t, *J* = 2.7 Hz, 1H). ES(+) 219.1 $[M+H]^+$.

<u>*N*-(6-(but-3-yn-1-yloxy)benzo[*d*]thiazol-2-yl)-2-chloroacetamide (SCLA2b)</u>

To a solution of 6-(but-3-yn-1-yloxy)benzo[*d*]thiazol-2-amine (7.0 mg, 0.032 mmol) in anhydrous DMF (1 mL) under nitrogen at 0 °C, was added potassium carbonate (13 mg, 0.096 mmol) and chloroacetyl chloride (7.7 µL, 0.096 mmol), and the reaction was stirred at room temperature for 2 h. Saturated ammonium chloride solution was added to the reaction mixture, and after extraction with ethyl acetate (x3), the combined organic layers were washed with brine, dried over sodium sulfate, and concentrated under reduced pressure. Purification with flash column chromatography (10-100% ethyl acetate in hexane) afforded the title compound (3.5 mg, yield 37%) as an off-white solid. R_f: 0.4 (hexane/ethyl acetate: 1/1). ^{1}H NMR (400 MHz, $CDCl_3$) δ 7.71 (d, *J* = 8.9 Hz, 1H), 7.33 (d, *J* = 2.3 Hz, 1H), 7.09 (dd, *J* = 8.6, 2.5 Hz, 1H), 4.31 (s, 2H), 4.16 (t, *J* = 6.9 Hz, 2H), 2.72 (td, *J* = 6.9, 2.7 Hz, 2H), 2.06 (t, *J* = 2.7 Hz, 1H). ^{13}C NMR (126 MHz, $CDCl_3$) δ 164.4, 156.2, 155.0, 122.1, 116.2, 105.6, 80.4, 70.2, 66.9, 42.2, 19.7. ES(-) *m/z* 293.0 $[M-H]^-$.

Probes SCLA3a and SCLA3b

Based on the structure of **SCLA3**, two probes were designed by adding the alkyne group in the 4-position of the piperidine ring **(SCLA3a)** or by replacing the piperidine with an acyclic amine carrying an alkyne group **(SCLA3b)**.

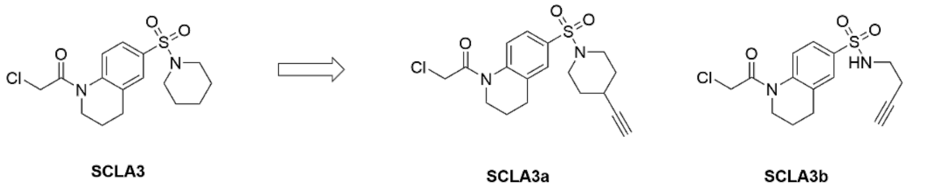

Scheme 5. Synthesis of probe SCLA3a.

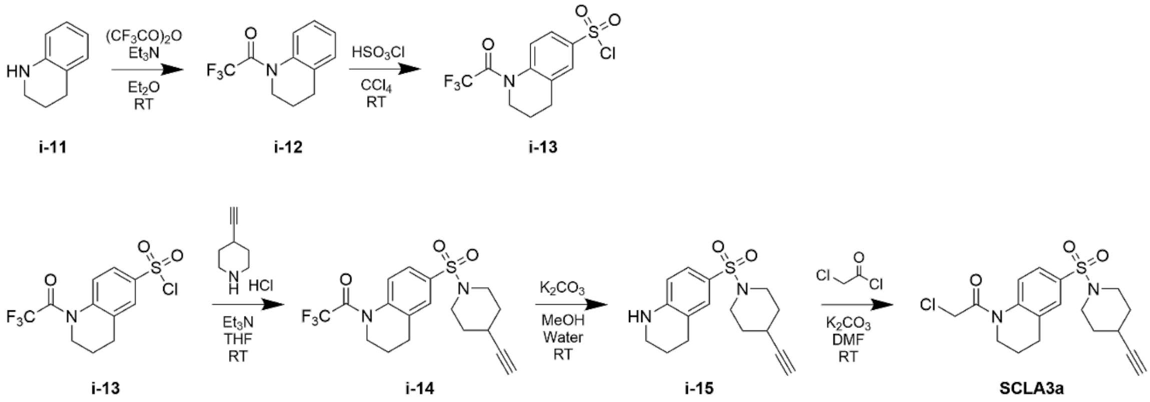

<u>1-(3,4-dihydroquinolin-1(2*H*)-yl)-2,2,2-trifluoroethan-1-one (i-12)</u>

To a solution of 1,2,3,4-tetrahydroquinoline (1.00 g, 7.51 mmol) in diethyl ether (10 mL), were added at 0 °C, triethylamine (3.14 mL, 22.5 mmol) and a solution of trifluoroacetic anhydride (2.09 mL, 15.0 mmol) in diethyl ether (10 mL) dropwise, and the reaction was stirred at room temperature for 24 h. Water was added to the mixture, and the phases separated. The aqueous layer was further extracted (x2) with ethyl acetate, and the combined organic layers were washed with brine, dried over sodium sulfate, and concentrated under reduced pressure. Purification with flash column chromatography (5-50% ethyl acetate in hexane) afforded the title compound (1.67 g, yield 97%) as a colourless oil. R_f: 0.5 (hexane/ethyl acetate: 8/2). ^{1}H NMR (400 MHz, $CDCl_3$) δ 7.77 (s, 1H), 7.28 – 7.18 (m, 3H), 3.86 (t, *J* = 6.2 Hz, 2H), 2.91 (s, 2H), 2.10 (p, *J* = 6.6 Hz, 2H). ^{19}F NMR (377 MHz, $CDCl_3$) δ -68.83. ^{13}C NMR (101 MHz, $CDCl_3$) δ 137.1, 129.4, 126.3, 124.5, 118.2, 115.4, 45.1, 26.1, 23.6. ES(+) *m/z* 230.1 $[M+H]^+$. *Synthesized according to* [24].

<u>1-(2,2,2-trifluoroacetyl)-1,2,3,4-tetrahydroquinoline-6-sulfonyl chloride (i-13)</u>

To a solution of 1-(3,4-dihydroquinolin-1(2*H*)-yl)-2,2,2-trifluoroethan-1-one (1650 mg, 7.20 mmol) in anhydrous carbon tetrachloride (20 mL) under nitrogen at 0 °C, was added chlorosulfonic acid (3.51 mL, 36.0 mmol) and the reaction mixture stirred at room temperature for 2 h. Water was added to the reaction mixture, and the phases separated. The aqueous layer was further extracted with dichloromethane (x2). The combined organic layers were washed with brine, dried over sodium sulfate, filtered, and concentrated under reduced pressure. Purification with flash column chromatography (10-100% ethyl acetate in hexane) afforded the title compound (450 mg, yield 19%) as a colourless oil which slowly crystallised to a white solid. R_f: 0.6 (hexane/ethyl acetate: 8/2). ^{1}H NMR (400 MHz, $CDCl_3$) δ 8.00 (d, *J* = 8.9 Hz, 1H), 7.92 – 7.84 (m, 2H), 3.91 (t, *J* = 5.9 Hz, 2H), 3.02 (t, *J* = 6.9 Hz, 2H), 2.22 – 2.10

(m, 2H). ^{19}F NMR (377 MHz, $CDCl_3$) δ -68.53. ES(+) *m/z* 327.9 $[M+H]^+$. *Synthesized according to* [24].

<u>1-(6-((4-ethynylpiperidin-1-yl)sulfonyl)-3,4-dihydroquinolin-1(2*H*)-yl)-2,2,2-trifluoroethan-1-one (i-14)</u>

To a solution of 1-(2,2,2-trifluoroacetyl)-1,2,3,4-tetrahydroquinoline-6-sulfonyl chloride (100 mg, 0.31 mmol) in anhydrous THF (5 mL) under nitrogen at 0 °C, was added triethylamine (0.13 mL, 0.92 mmol) and 4-ethynylpiperidine hydrochloride (53 mg, 0.37 mmol), and the reaction stirred at room temperature for 6 h. The mixture was concentrated, water was added to the residue, and after extraction with ethyl acetate (x3), the combined organic layers were washed with brine, dried over sodium sulfate, and concentrated under reduced pressure. Purification with flash column chromatography (5-60% ethyl acetate in hexane) afforded the title compound (105 mg, yield 86%) as a white solid. R_f: 0.7 (hexane/ethyl acetate: 1/1). ^{1}H NMR (400 MHz, DMSO-d_6) δ 7.85 (s, 1H), 7.64 (s, 1H), 7.59 (dd, *J* = 8.7, 2.2 Hz, 1H), 3.84 (t, *J* = 6.0 Hz, 2H), 3.29 – 3.19 (m, 2H), 2.98 – 2.90 (m, 3H), 2.73 – 2.63 (m, 2H), 2.08 – 1.97 (m, 2H), 1.89 – 1.78 (m, 2H), 1.61 – 1.48 (m, 2H). (1xH obscured by DMSO peak). ^{19}F NMR (377 MHz, DMSO-d_6) δ -67.96. ^{13}C NMR (101 MHz, DMSO-d_6) δ 140.3, 132.7, 128.4, 125.1, 125.0, 86.2, 72.5, 44.9, 44.9, 44.4, 30.4, 25.6, 25.0, 22.5. ES(+) *m/z* 401.0 $[M+H]^+$.

<u>4-ethynylpiperidine hydrochloride was synthesized using the commercially available Boc-protected reagent</u>

To a solution of tert-butyl 4-ethynylpiperidine-1-carboxylate (300 mg, 1.43 mmol) in dioxane (5 mL), was added HCl in dioxane 4 M (15 mL) at 0 °C, and the reaction stirred at room temperature for 3 h. The mixture was concentrated under reduced pressure, and the residue was triturated with diethyl ether to provide 4-ethynylpiperidine hydrochloride (160 mg, yield 77%) as a white solid. ^{1}H NMR (400 MHz, DMSO-d_6) δ 9.00 (s, 2H), 3.18 – 3.05 (m, 3H), 2.99 – 2.86 (m, 2H), 2.77 – 2.66 (m, 1H), 2.00 – 1.88 (m, 2H), 1.76 – 1.65 (m, 2H).

<u>6-((4-ethynylpiperidin-1-yl)sulfonyl)-1,2,3,4-tetrahydroquinoline (i-15)</u>

To a solution of 1-(6-((4-ethynylpiperidin-1-yl)sulfonyl)-3,4-dihydroquinolin-1(2*H*)-yl)-2,2,2-trifluoroethan-1-one (95 mg, 0.24 mmol) in methanol (10 mL) and water (4 mL), was added potassium carbonate (656 mg, 4.75 mmol) and the reaction stirred at room temperature for 2 h. The mixture was concentrated, water was added to the residue, and after extraction with ethyl acetate (x3), the combined organic layers were washed with brine, dried over sodium sulfate, and concentrated under reduced pressure to afford the title compound (65 mg, yield 90%) as a colourless oil which slowly crystallised to a white solid. R_f: 0.5 (hexane/ethyl acetate: 1/1). ^{1}H NMR (400 MHz, DMSO-d_6) δ 7.20 – 7.12 (m, 2H), 6.69 (s, 1H), 6.50 (d, *J* = 8.4 Hz, 1H), 3.26 – 3.20 (m, 2H), 3.19 – 3.10 (m, 2H), 2.92 (d, *J* = 2.3 Hz, 1H), 2.69 (t, *J* = 6.3 Hz, 2H), 2.56 – 2.51 (m, 2H), 2.45 – 2.40 (m, 1H), 1.87 – 1.74 (m, 4H), 1.58 – 1.45 (m, 2H). ^{13}C NMR (101 MHz, DMSO-d_6) δ 149.3, 128.6, 127.0, 119.1, 118.8, 112.1, 86.5, 72.3, 44.4, 40.4, 30.5, 26.5, 25.1, 20.4. ES(+) *m/z* 305.1 $[M+H]^+$.

<u>2-chloro-1-(6-((4-ethynylpiperidin-1-yl)sulfonyl)-3,4-dihydroquinolin-1(2*H*)-yl)ethan-1-one (SCLA3a)</u>

To a solution of 6-((4-ethynylpiperidin-1-yl)sulfonyl)-1,2,3,4-tetrahydroquinoline (60 mg, 0.20 mmol) in anhydrous DMF (2 mL) under nitrogen at 0 °C, was added potassium carbonate (54 mg, 0.39 mmol) and chloroacetyl chloride (24 µL, 0.30 mmol), and the reaction stirred at

room temperature for 1 h. Saturated ammonium chloride solution was added to the reaction mixture, and after extraction with ethyl acetate (x3), the combined organic layers were washed with brine, dried over sodium sulfate, and concentrated under reduced pressure. Purification with flash column chromatography (10-100% ethyl acetate in hexane) afforded the title compound (55 mg, yield 73%) as a white solid. R_f: 0.5 (hexane/ethyl acetate: 1/1). ^{1}H NMR (400 MHz, DMSO-d_6) δ 7.90 (d, J = 8.5 Hz, 1H), 7.56 (s, 1H), 7.52 (dd, J = 8.8, 2.2 Hz, 1H), 4.64 (s, 2H), 3.75 (t, J = 6.1 Hz, 2H), 3.29 – 3.19 (m, 2H), 2.93 (d, J = 2.4 Hz, 1H), 2.83 (t, J = 6.5 Hz, 2H), 2.65 (t, J = 10.3 Hz, 2H), 2.49 – 2.41 (m, 1H), 1.93 (p, J = 6.2 Hz, 2H), 1.88 – 1.78 (m, 2H), 1.61 – 1.47 (m, 2H). ^{13}C NMR (101 MHz, DMSO-d_6) δ 166.2, 141.8, 132.5, 130.8, 127.9, 125.2, 124.4, 86.2, 72.5, 44.6, 44.4, 43.7, 30.4, 26.4, 25.0, 22.7. ES(+) m/z 381.1 $[M+H]^+$.

Scheme 6. Synthesis of probe SCLA3b.

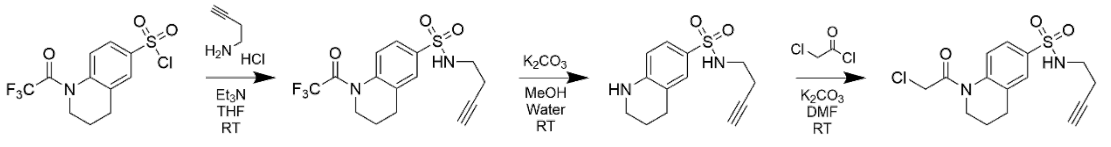

i-13 i-16 i-17 SCLA3b

*N*-(but-3-yn-1-yl)-1-(2,2,2-trifluoroacetyl)-1,2,3,4-tetrahydroquinoline-6-sulfonamide (i-16)

To a solution of 1-(2,2,2-trifluoroacetyl)-1,2,3,4-tetrahydroquinoline-6-sulfonyl chloride (150 mg, 0.46 mmol) in anhydrous THF (6 mL) under nitrogen at 0 °C, was added triethylamine (0.19 mL, 1.37 mmol) and but-3-yn-1-amine hydrochloride (58 mg, 0.55 mmol), and the reaction stirred at room temperature for 4 h. The mixture was concentrated, water was added to the residue, and after extraction with ethyl acetate (x3), the combined organic layers were washed with brine, dried over sodium sulfate, and concentrated under reduced pressure. Purification with flash column chromatography (10-100% ethyl acetate in hexane) afforded the title compound (125 mg, yield 76%) as a white solid. R_f: 0.7 (hexane/ethyl acetate: 1/1). ^{1}H NMR (400 MHz, DMSO-d_6) δ 7.85 (t, J = 5.9 Hz, 1H), 7.79 (s, 1H), 7.69 (d, J = 2.2 Hz, 1H), 7.64 (dd, J = 8.6, 2.2 Hz, 1H), 3.83 (t, J = 6.0 Hz, 2H), 2.96 – 2.82 (m, 5H), 2.28 (td, J = 7.1, 2.7 Hz, 2H), 2.02 (p, J = 6.4 Hz, 2H). ^{19}F NMR (377 MHz, DMSO-d_6) δ -68.07. ^{13}C NMR (101 MHz, DMSO-d_6) δ 139.7, 137.7, 127.4, 125.0, 124.0, 117.7, 114.9, 81.5, 72.6, 44.8, 41.6, 25.6, 22.5, 19.4. ES(-) m/z 359.1 $[M-H]^-$.

*N*-(but-3-yn-1-yl)-1,2,3,4-tetrahydroquinoline-6-sulfonamide (i-17)

To a solution of *N*-(but-3-yn-1-yl)-1-(2,2,2-trifluoroacetyl)-1,2,3,4-tetrahydroquinoline-6-sulfonamide (45 mg, 0.12 mmol) in methanol (5 mL) and water (2 mL), was added potassium carbonate (345 mg, 2.50 mmol) and the reaction stirred at room temperature for 2 h. The mixture was concentrated, water was added to the residue, and after extraction with ethyl acetate (x3), the combined organic layers were washed with brine, dried over sodium sulfate, and concentrated under reduced pressure. Purification with flash column chromatography (10-100% ethyl acetate in hexane) afforded the title compound (20 mg, yield 61%) as a colourless oil which slowly crystallised to a white solid. R_f: 0.5 (hexane/ethyl acetate: 1/1). ^{1}H NMR (400 MHz, DMSO-d_6) δ 7.27 – 7.18 (m, 3H), 6.61 – 6.54 (m, 1H), 6.47 (d, J = 8.3 Hz, 1H), 3.26 – 3.17 (m, 2H), 2.83 (t, J = 2.7 Hz, 1H), 2.75 (q, J = 7.0 Hz, 2H), 2.68 (t, J = 6.3 Hz, 2H), 2.24

(td, J = 7.3, 2.7 Hz, 2H), 1.83 – 1.72 (m, 2H). ^{13}C NMR (101 MHz, DMSO-d_6) δ 148.7, 127.7, 126.0, 124.1, 119.1, 112.0, 81.8, 72.4, 41.6, 40.4, 26.6, 20.5, 19.2. ES(-) *m/z* 263.1 [M-H]$^-$.

***N*-(but-3-yn-1-yl)-1-(2-chloroacetyl)-1,2,3,4-tetrahydroquinoline-6-sulfonamide (SCLA3b)**

To a solution of *N*-(but-3-yn-1-yl)-1,2,3,4-tetrahydroquinoline-6-sulfonamide (15 mg, 0.057 mmol) in anhydrous DMF (2 mL) under nitrogen at 0 °C, was added potassium carbonate (54 mg, 0.39 mmol) and chloroacetyl chloride (24 µL, 0.30 mmol) and the reaction stirred at room temperature for 18 h. Saturated ammonium chloride solution was added to the reaction mixture, and after extraction with ethyl acetate (x3), the combined organic layers were washed with brine, dried over sodium sulfate, and concentrated under reduced pressure. Purification with flash column chromatography (10-100% ethyl acetate in hexane) afforded the title compound (14 mg, yield 72%) as a white solid. R_f: 0.4 (hexane/ethyl acetate: 1/1). ^{1}H NMR (400 MHz, DMSO-d_6) δ 7.83 (d, J = 8.6 Hz, 1H), 7.76 (t, J = 6.0 Hz, 1H), 7.62 (d, J = 2.2 Hz, 1H), 7.58 (dd, J = 8.6, 2.3 Hz, 1H), 4.63 (s, 2H), 3.74 (t, J = 6.2 Hz, 2H), 2.89 – 2.78 (m, 5H), 2.28 (td, J = 7.1, 2.7 Hz, 2H), 1.93 (p, J = 6.4 Hz, 2H). ^{13}C NMR (101 MHz, DMSO-d_6) δ 166.0, 141.2, 135.9, 132.4, 127.0, 124.4, 124.2, 81.6, 72.5, 44.5, 43.6, 41.6, 26.3, 22.8, 19.4. ES(-) *m/z* 339.0 [M-H]$^-$.

Probes SCLA4a and SCLA4b

Based on the structure of **SCLA4**, two probes were designed by replacing the tetrahydronaphthalene ring with a phenyl ring with an alkyne group attached via an ether linker **(SCLA4a)** or by adding the alkyne in the 4-position of the aromatic ring attached to pyrazole-*N* **(SCLA4b).**

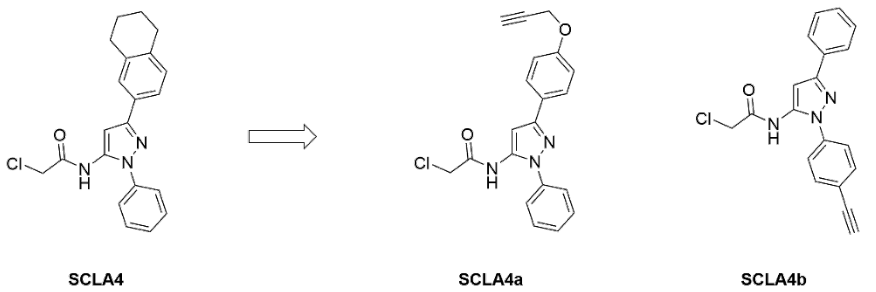

Scheme 7. Synthesis of probe SCLA4a.

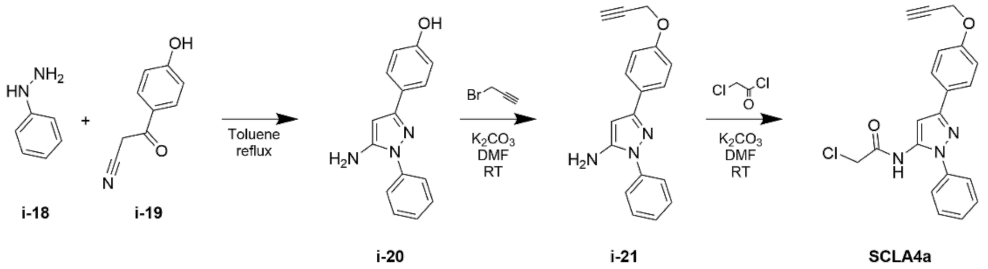

4-(5-amino-1-phenyl-1*H*-pyrazol-3-yl)phenol (i-20)

A mixture of phenylhydrazine (183 µL, 1.86 mmol) and 4-hydroxybenzoylacetonitrile (300 mg, 1.86 mmol) in toluene (5 mL) was heated to reflux for 16 h. The mixture was diluted with ethyl acetate, washed with brine (x2), and the organic phase was dried over sodium sulfate and concentrated under reduced pressure. Purification with flash column chromatography (20-60% ethyl acetate in hexane) afforded the title compound (385 mg, yield 82%) as a pale-yellow solid. R_f: 0.4 (hexane/ethyl acetate: 1/1). ^{1}H NMR (400 MHz, DMSO-d_6) δ 9.48 (s, 1H), 7.68 – 7.61 (m, 2H), 7.59 – 7.53 (m, 2H), 7.48 (t, *J* = 7.9 Hz, 2H), 7.35 – 7.26 (m, 1H), 6.81 – 6.73 (m, 2H), 5.79 (s, 1H), 5.39 (s, 2H). ES(+) *m/z* 252.1 $[M+H]^+$. *Synthesized according to the procedure reported for analogues in* [25]. *Synthesis of this compound has also been reported in* [26] *using different conditions*.

1-phenyl-3-(4-(prop-2-yn-1-yloxy)phenyl)-1*H*-pyrazol-5-amine (i-21)

To a solution of 4-(5-amino-1-phenyl-1*H*-pyrazol-3-yl)phenol (50 mg, 0.20 mmol) in anhydrous DMF (2 mL) under nitrogen, was added cesium carbonate (130 mg, 0.40 mmol) and propargyl bromide in toluene 80% w/w (23 µL, 0.21 mmol), and the reaction stirred at room temperature for 16 h. The reaction mixture was diluted with ethyl acetate and washed with lithium chloride solution 5% (x2) and brine. The combined organic layers were dried over sodium sulfate and concentrated under reduced pressure. Purification with flash column chromatography (20-50% ethyl acetate in hexane) afforded the title compound (42 mg, 73%) as a pale-yellow solid. R_f: 0.5 (hexane/ethyl acetate: 1/1). ^{1}H NMR (400 MHz, DMSO-d_6) δ 7.69 (d, *J* = 8.6 Hz, 2H), 7.65 (d, *J* = 7.9 Hz, 2H), 7.50 (t, *J* = 7.7 Hz, 2H), 7.33 (t, *J* = 7.4 Hz, 1H), 7.00 (d, *J* = 8.7 Hz, 2H), 5.85 (s, 1H), 5.42 (s, 2H), 4.82 (d, *J* = 2.4 Hz, 2H), 3.59 (t, *J* = 2.3 Hz, 1H). ES(+) *m/z* 290.1 $[M+H]^+$.

2-chloro-*N*-(1-phenyl-3-(4-(prop-2-yn-1-yloxy)phenyl)-1*H*-pyrazol-5-yl)acetamide (SCLA4a)

To a solution of 1-phenyl-3-(4-(prop2-yn-1-yloxy)phenyl)-1*H*-pyrazol-5-amine (35 mg, 0.12 mmol) in anhydrous DMF (2 mL) under nitrogen at 0 °C, was added potassium carbonate (33 mg, 0.24 mmol) and chloroacetyl chloride (29 µL, 0.36 mmol), and the reaction stirred at room temperature for 16 h. Lithium chloride solution 5% was added to the reaction mixture, and after extraction with ethyl acetate (x3), the combined organic layers were washed with brine, dried over sodium sulfate, and concentrated under reduced pressure. Purification with flash column chromatography (20-50% ethyl acetate in hexane) afforded the title compound (32 mg, yield 72%) as an off-white solid. R_f: 0.5 (hexane/ethyl acetate: 1/1). ^{1}H NMR (400 MHz, DMSO-d_6) δ 10.41 (s, 1H), 7.84 – 7.77 (m, 2H), 7.60 – 7.50 (m, 4H), 7.46 – 7.39 (m, 1H), 7.09 – 7.02 (m, 2H), 6.89 (s, 1H), 4.84 (d, *J* = 2.4 Hz, 2H), 4.28 (s, 2H), 3.60 (t, *J* = 2.4 Hz, 1H). ^{13}C NMR (101 MHz, DMSO-d_6) δ 165.5, 157.2, 150.0, 138.4, 136.3, 129.3, 127.6, 126.5, 126.0, 123.7, 115.1, 99.5, 79.2, 78.3, 55.5, 42.6. ES(+) *m/z* 366.1 $[M+H]^+$.

Scheme 8. Synthesis of probe SCLA4b.

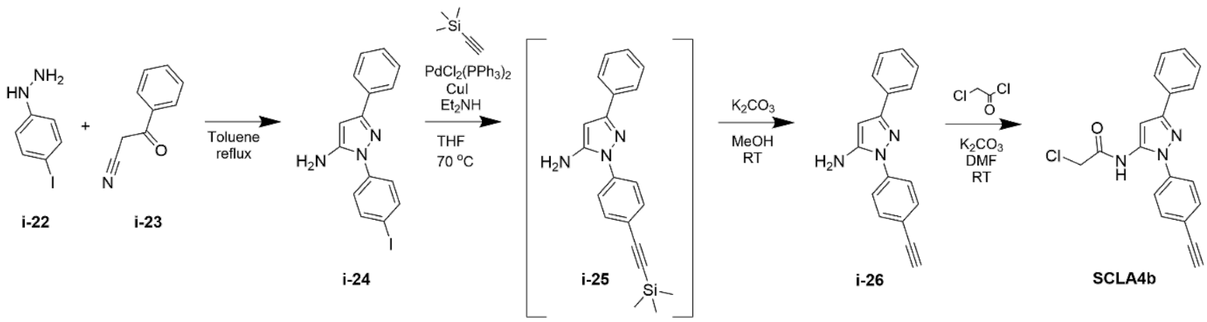

1-(4-iodophenyl)-3-phenyl-1*H*-pyrazol-5-amine (i-24)

A mixture of (4-iodophenyl)hydrazine (500 mg, 2.14 mmol) and 3-oxo-3-phenylpropanenitrile (310 mg, 2.14 mmol) in toluene (4 mL) was heated to reflux for 16 h. The mixture was diluted with ethyl acetate, washed with brine (x2), and the organic phase was dried over sodium sulfate and concentrated under reduced pressure. Purification with flash column chromatography (5-30% ethyl acetate in hexane) afforded the title compound (378 mg, yield 49%) as a light brown solid. R_f: 0.6 (hexane/ethyl acetate: 7/3). ^{1}H NMR (400 MHz, DMSO-d_6) δ 7.88 – 7.80 (m, 2H), 7.78 – 7.71 (m, 2H), 7.54 – 7.45 (m, 2H), 7.38 (t, *J* = 7.5 Hz, 2H), 7.33 – 7.25 (m, 1H), 5.92 (s, 1H), 5.53 (s, 2H). ES(+) *m/z* 362.0 $[M+H]^+$. *Synthesized according to the procedure reported for analogues in* [25]. *Synthesis of this compound has also been reported in* [27] *using different conditions*.

1-(4-ethynylphenyl)-3-phenyl-1*H*-pyrazol-5-amine (i-25)

To a solution of 1-(4-iodophenyl)-3-phenyl-1*H*-pyrazol-5-amine (180 mg, 0.50 mmol) in anhydrous tetrahydrofuran (15 mL), evacuated and purged with nitrogen, were added triethylamine (0.35 mL, 2.5 mmol), (trimethylsilyl)acetylene (0.21 mL, 1.5 mmol), copper(I) iodide (19 mg, 0.10 mmol) and bis(triphenylphosphine)palladium(II) dichloride (35 mg, 0.050 mmol) and the reaction was heated to 70 °C for 24 h. The reaction mixture was filtered through a pad of celite, and the filtrate was concentrated under vacuum. To form the deprotected product, the residue was dissolved in methanol (10 mL), and potassium carbonate (689 mg, 4.98 mmol) was added. The reaction was stirred at room temperature for 2 h. Then, the mixture was concentrated, water was added to the residue, and after extraction with ethyl acetate (x3), the combined organic layers were washed with brine, dried over sodium sulfate, and concentrated under reduced pressure. Purification with flash column chromatography (10-40% ethyl acetate in hexane) afforded the title compound (35 mg, yield 27%) as a light brown solid. R_f: 0.3 (hexane/ethyl acetate: 8/2). ^{1}H NMR (400 MHz, DMSO-d_6) δ 7.79 – 7.70 (m, 4H), 7.60 (dq, *J* = 9.0, 2.2 Hz, 2H), 7.39 (t, *J* = 7.5 Hz, 2H), 7.35 –7.28 (m, 1H), 5.94 (s, 1H), 5.57 (s, 2H), 4.25 (s, 1H). ES(+) *m/z* 260.1 $[M+H]^+$.

2-chloro-*N*-(1-(4-ethynylphenyl)-3-phenyl-1*H*-pyrazol-5-yl)acetamide (SCLA4b)

To a solution of 1-(4-ethynylphenyl)-3-phenyl-1*H*-pyrazol-5-amine (25 mg, 0.096 mmol) in anhydrous DMF (2 mL) under nitrogen at 0 °C, was added potassium carbonate (27 mg, 0.19 mmol) and chloroacetyl chloride (23 µL, 0.29 mmol), and the reaction stirred at room temperature for 2 h. Lithium chloride solution 5% was added to the reaction mixture, and after

extraction with ethyl acetate (x3), the combined organic layers were washed with brine, dried over sodium sulfate, and concentrated under reduced pressure. Purification with flash column chromatography (10-40% ethyl acetate in hexane) afforded the title compound (30 mg, yield 93%) as an off-white solid. R_f: 0.3 (hexane/ethyl acetate: 7/3). ^1H NMR (400 MHz, DMSO-d_6) δ 10.50 (s, 1H), 7.90 – 7.85 (m, 2H), 7.67 – 7.59 (m, 4H), 7.48 – 7.42 (m, 2H), 7.39 – 7.34 (m, 1H), 6.97 (s, 1H), 4.32 (s, 1H), 4.30 (s, 2H). ^{13}C NMR (101 MHz, DMSO-d_6) δ 165.5, 150.7, 138.5, 136.6, 132.7, 132.4, 128.8, 128.3, 125.3, 123.6, 120.8, 100.3, 82.7, 81.9, 42.6. ES(+) *m/z* 336.1 $[M+H]^+$.

NMR spectra of synthesized probes

SCLA1a – ^{1}H-NMR

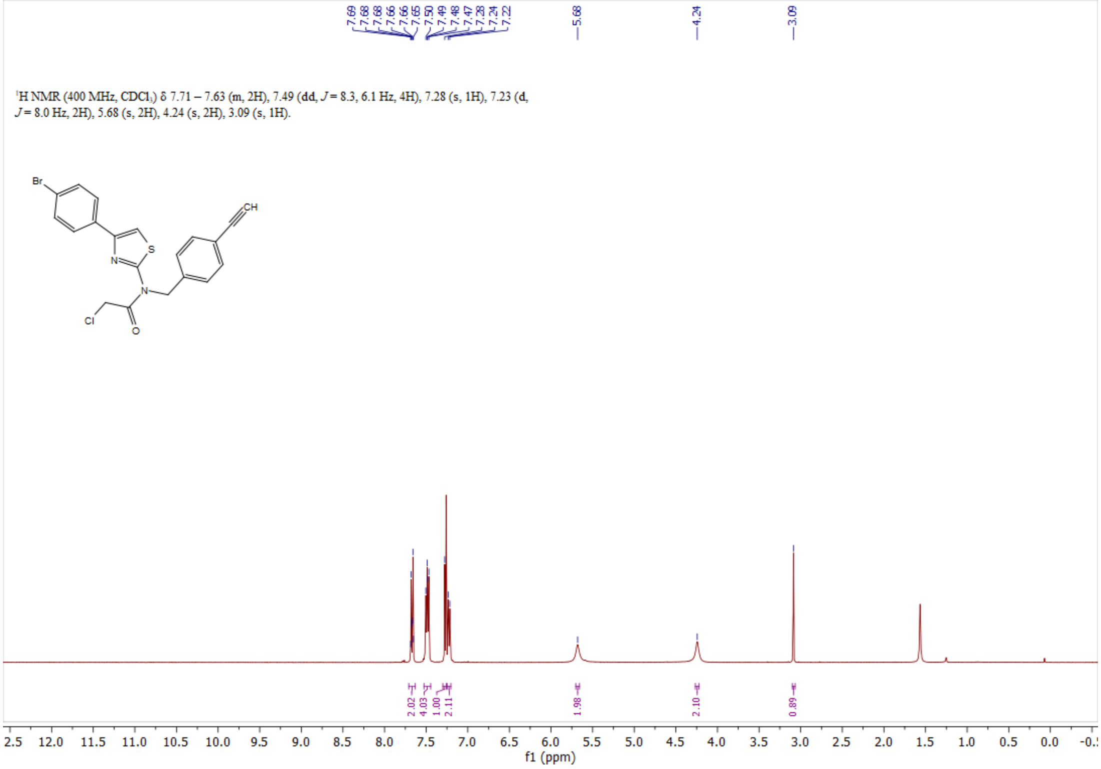

SCLA1a – ^{13}C-NMR

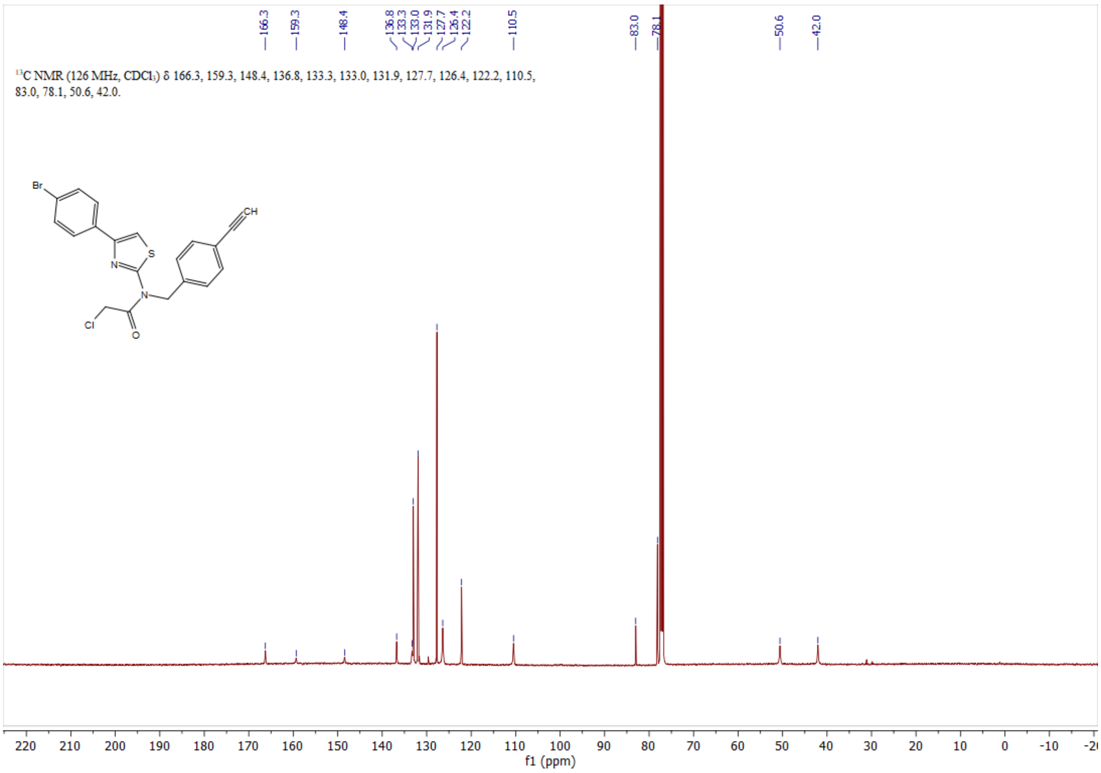

SCLA1b – ^{1}H-NMR

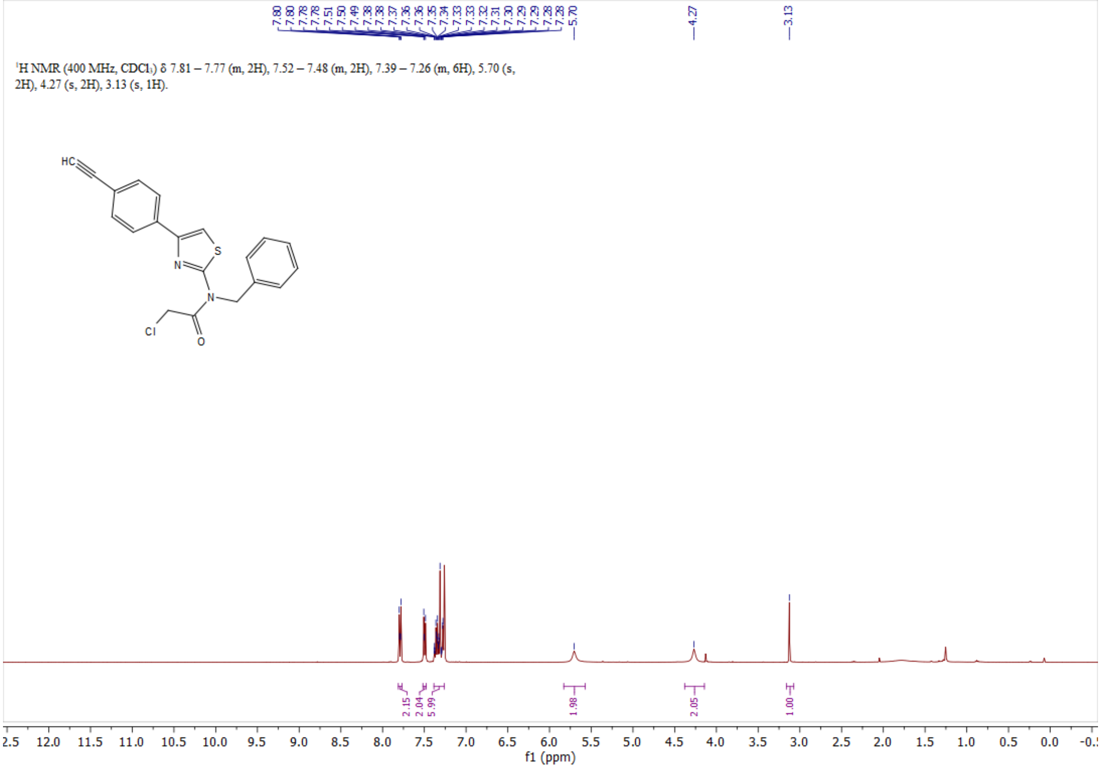

SCLA1b – ^{13}C-NMR

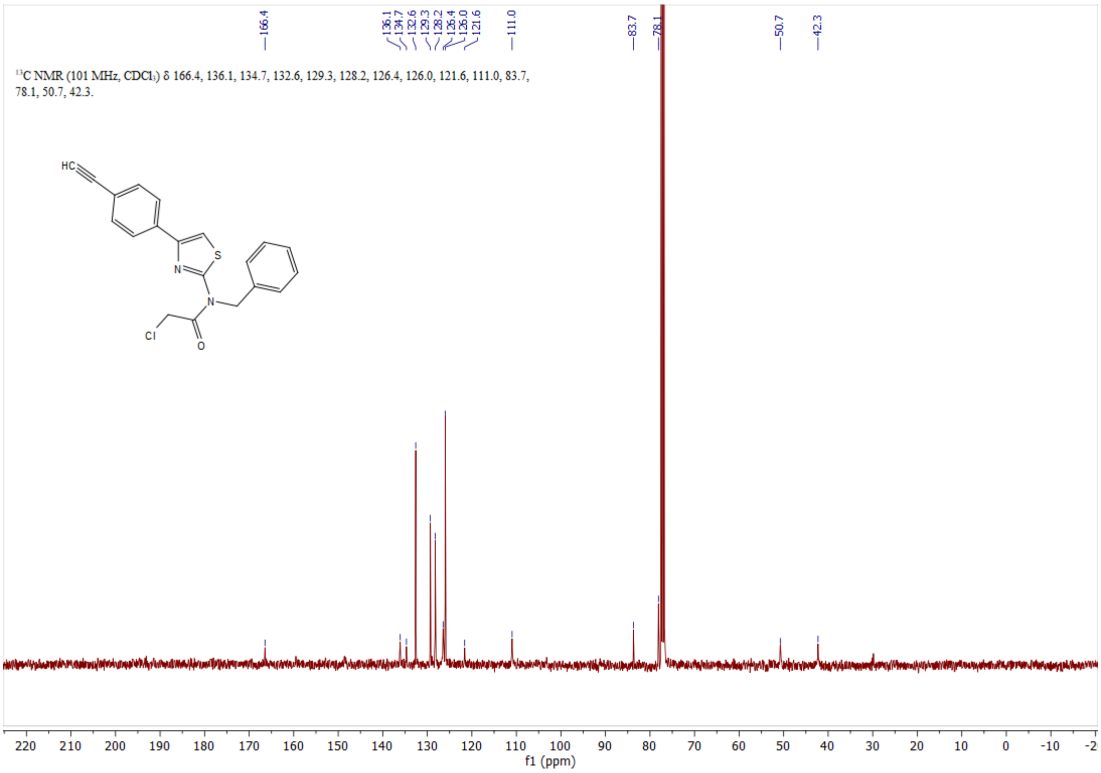

SCLA2a – ^{1}H-NMR

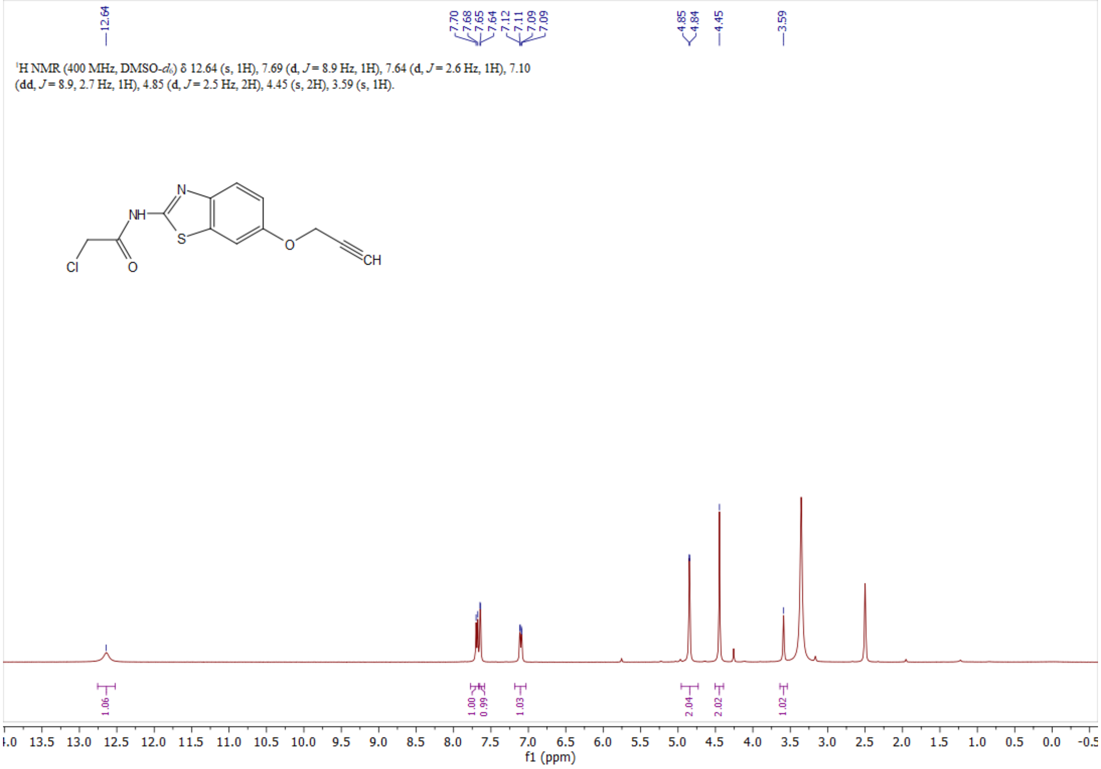

SCLA2a – ^{13}C-NMR

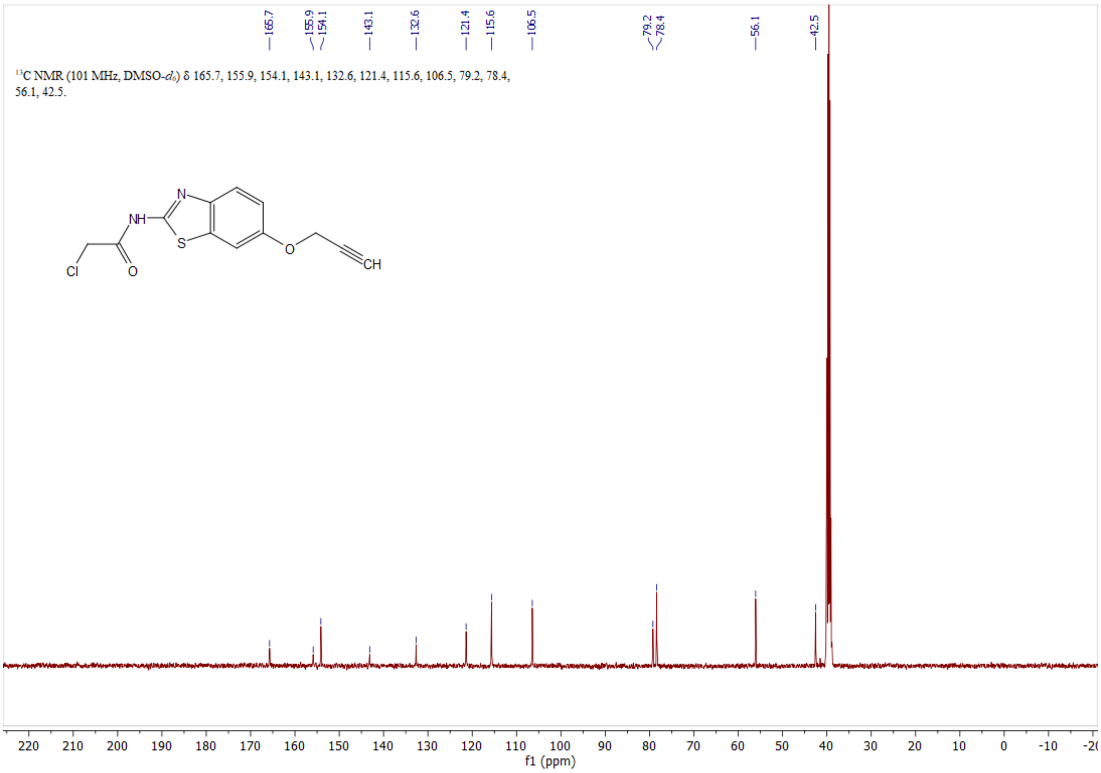

SCLA2b – ^{1}H-NMR

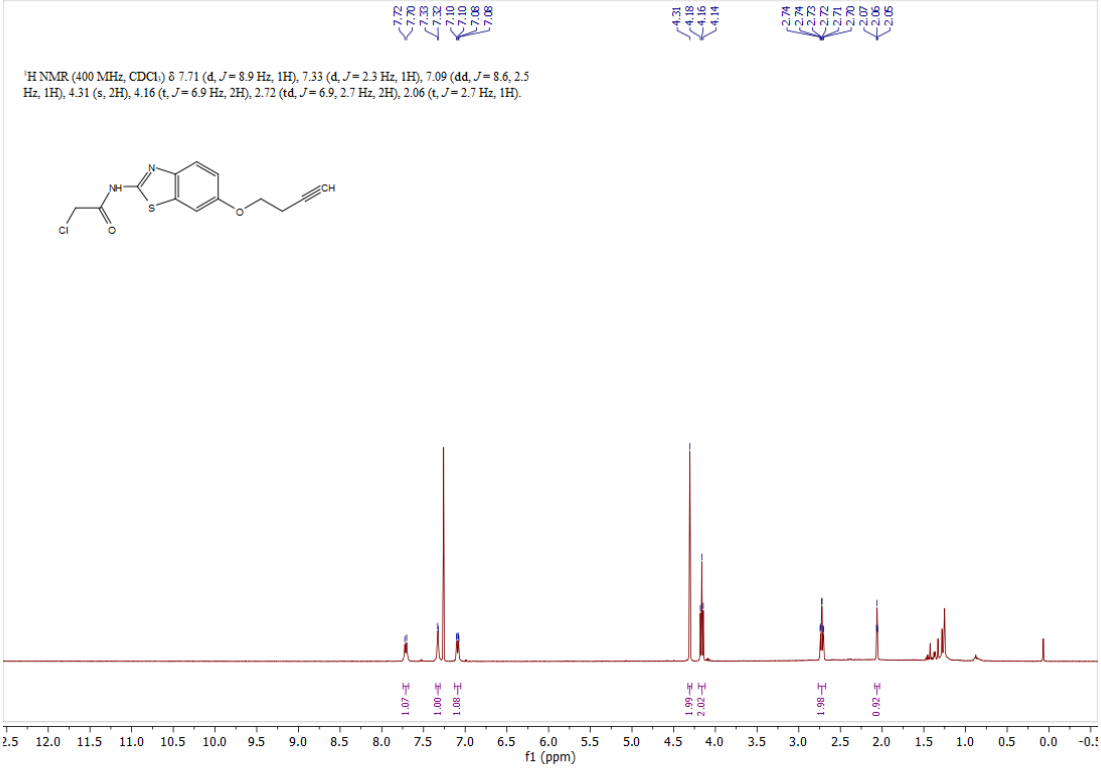

SCLA2b – ^{13}C-NMR

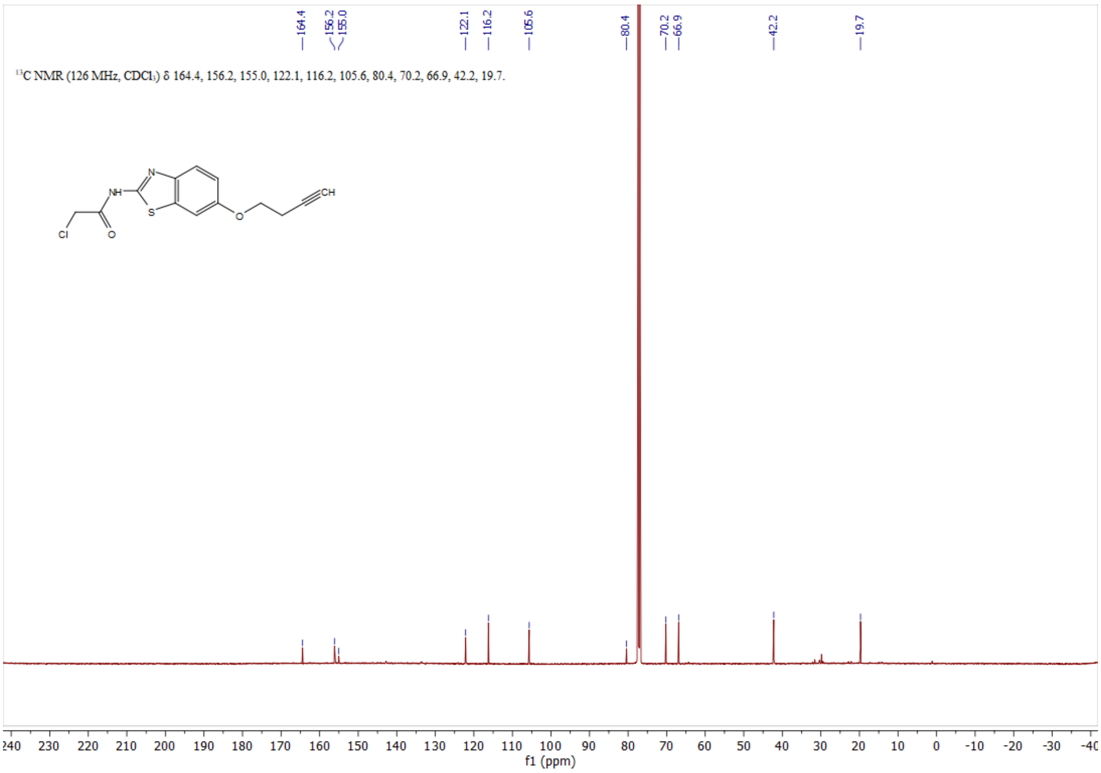

SCLA3a – ^{1}H-NMR

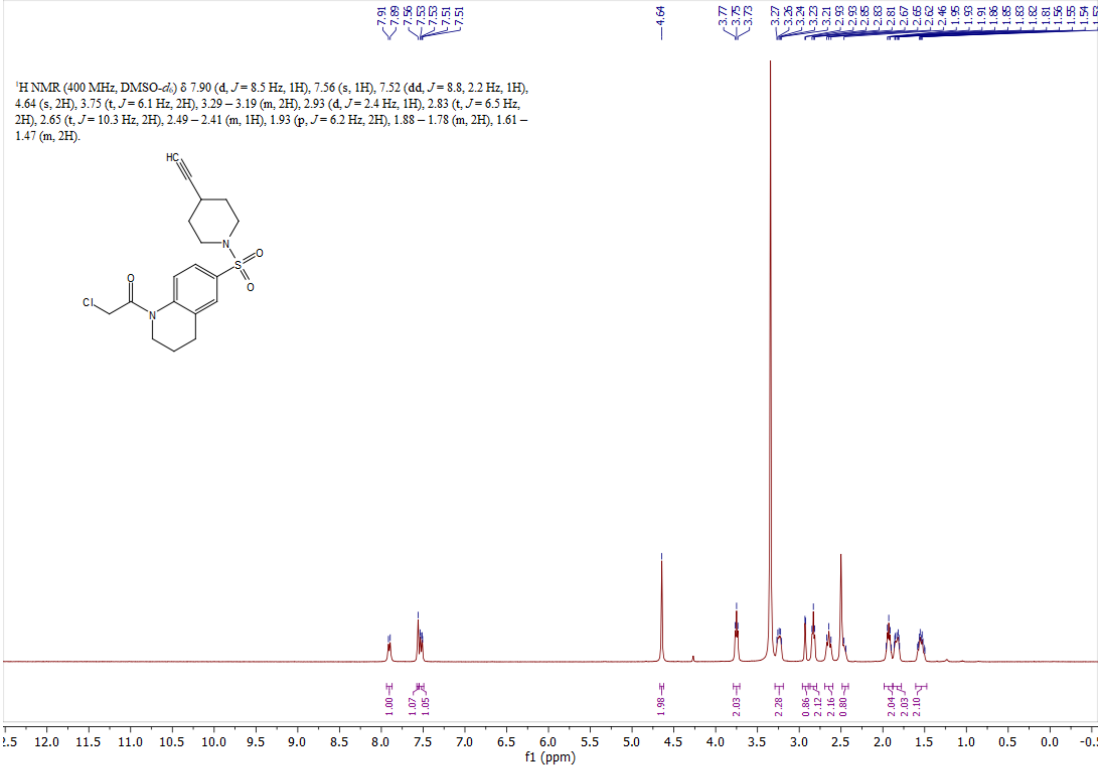

SCLA3a – ^{13}C-NMR

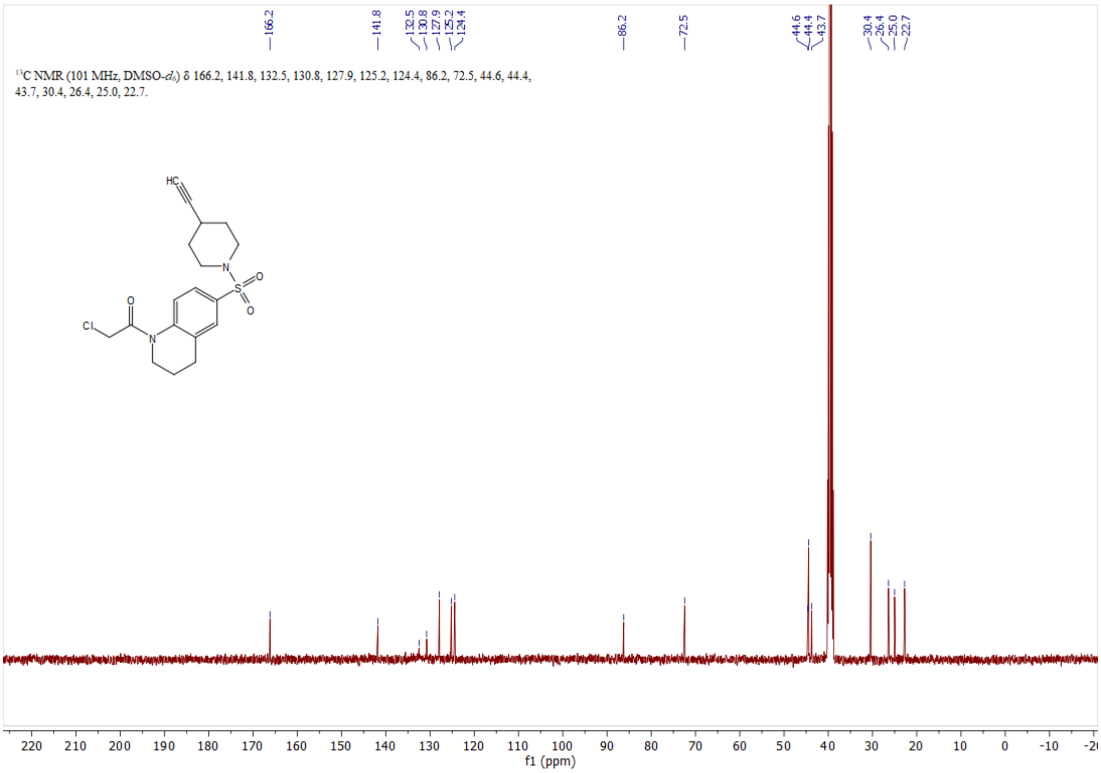

SCLA3b – ^{1}H-NMR

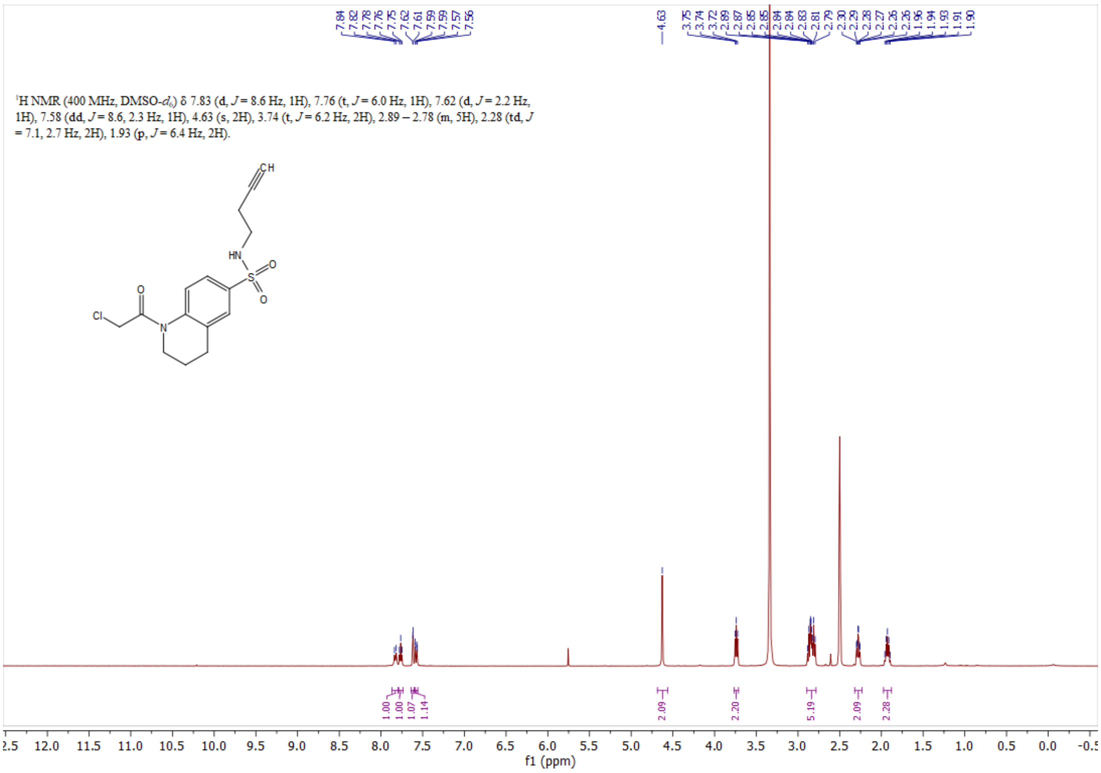

SCLA3b – ^{13}C-NMR

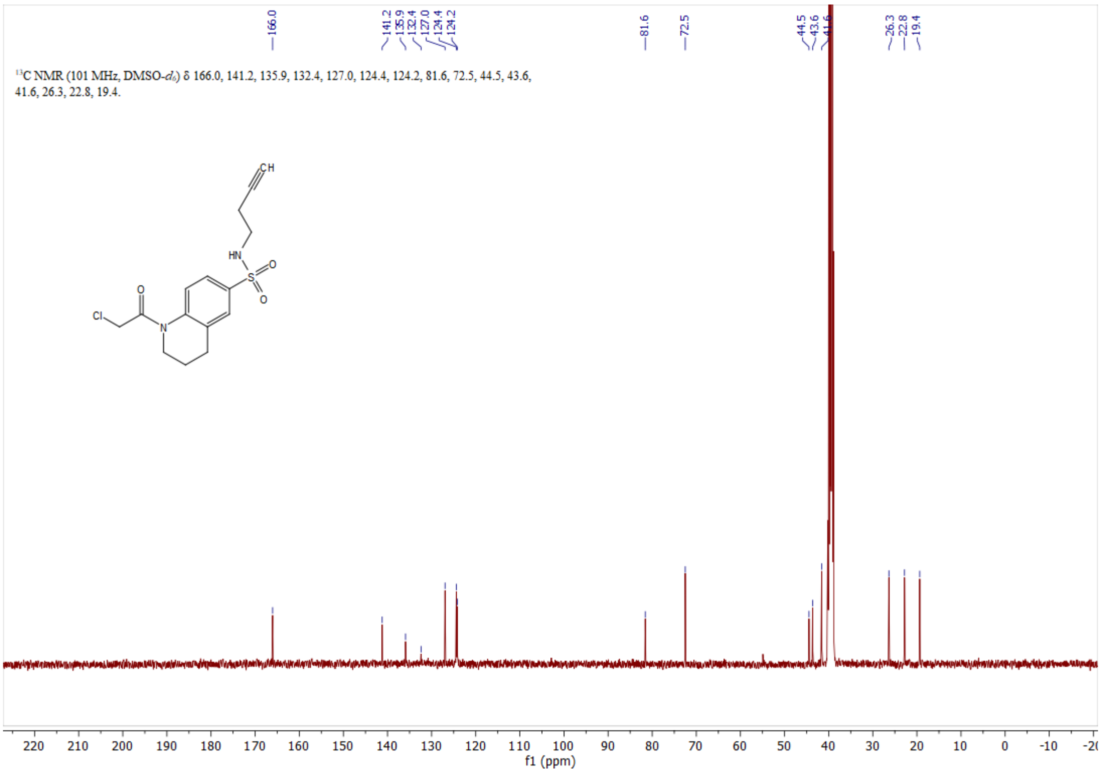

SCLA4a – ^{1}H-NMR

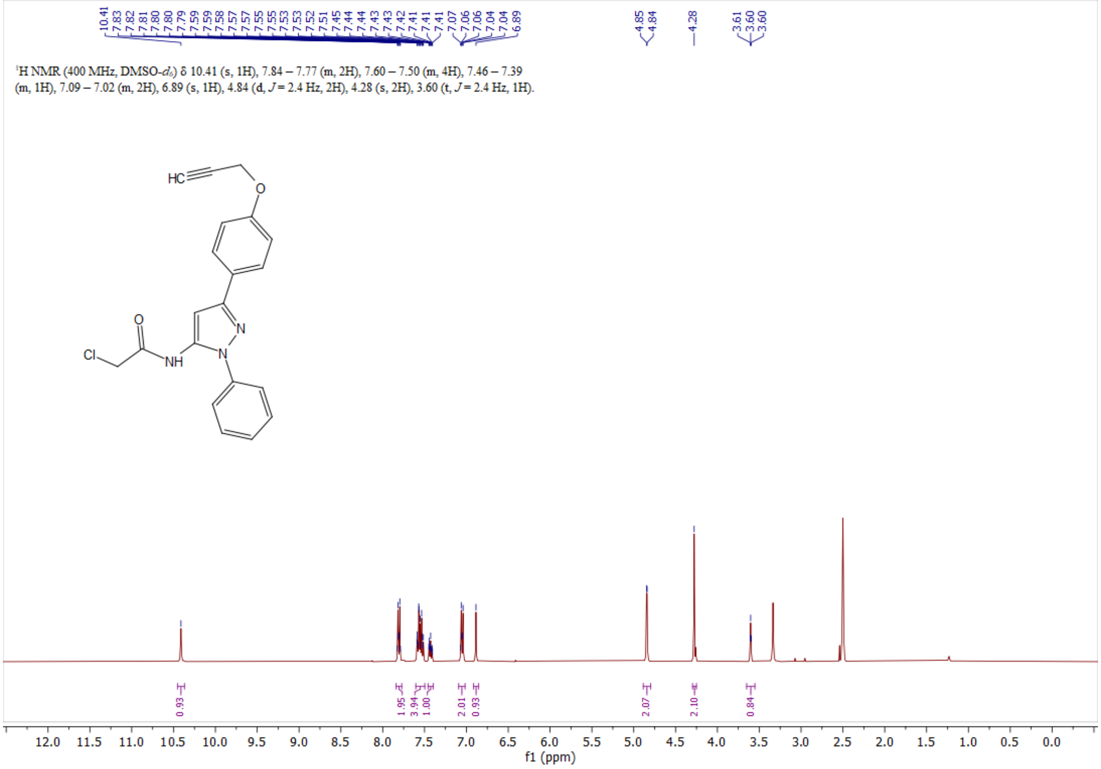

SCLA4a – ^{13}C-NMR

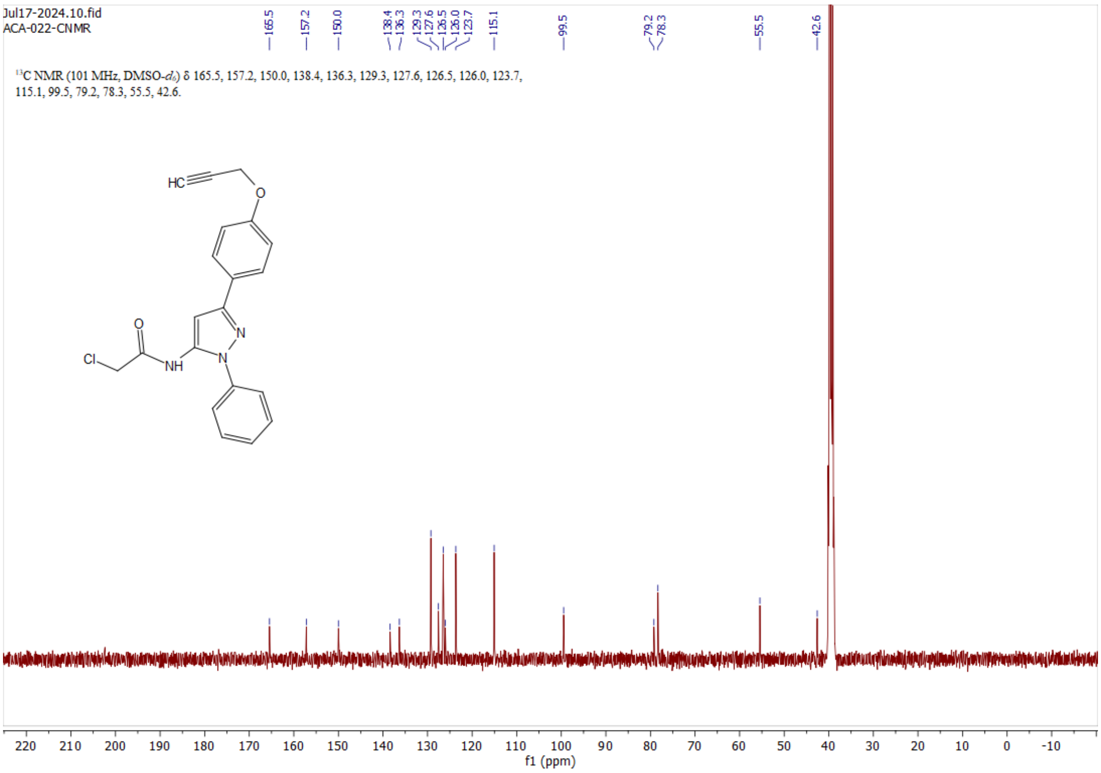

SCLA4b – ^{1}H-NMR

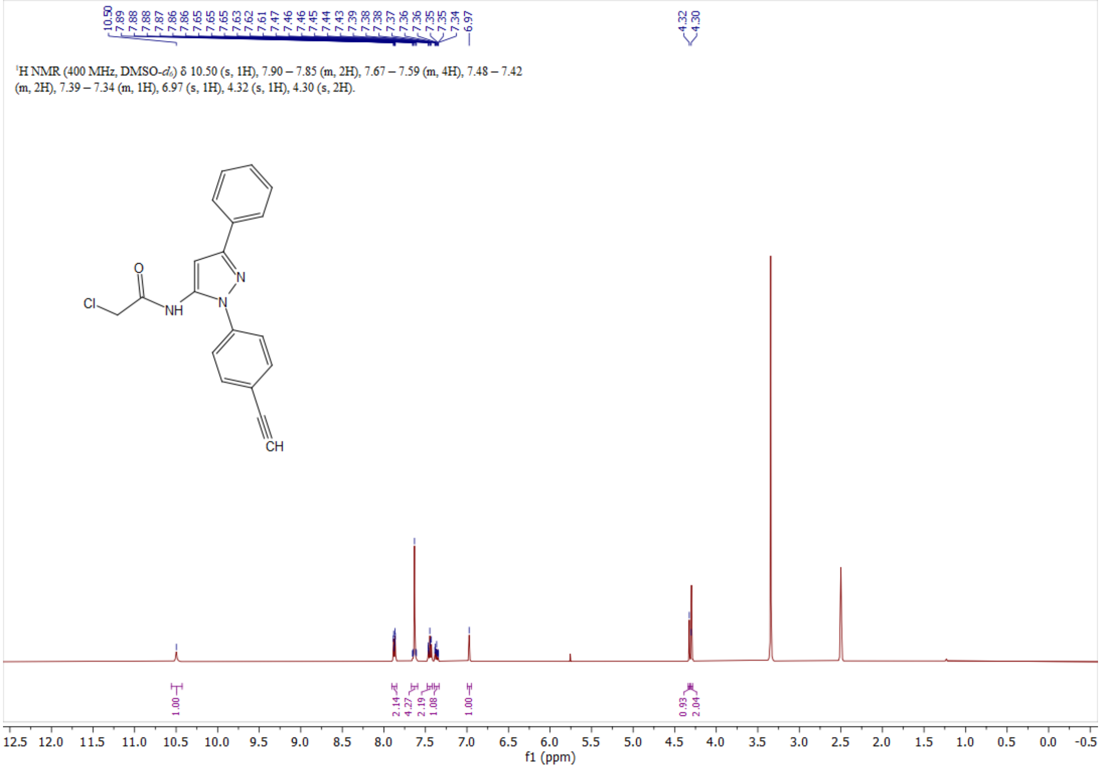

SCLA4b – ^{13}C-NMR

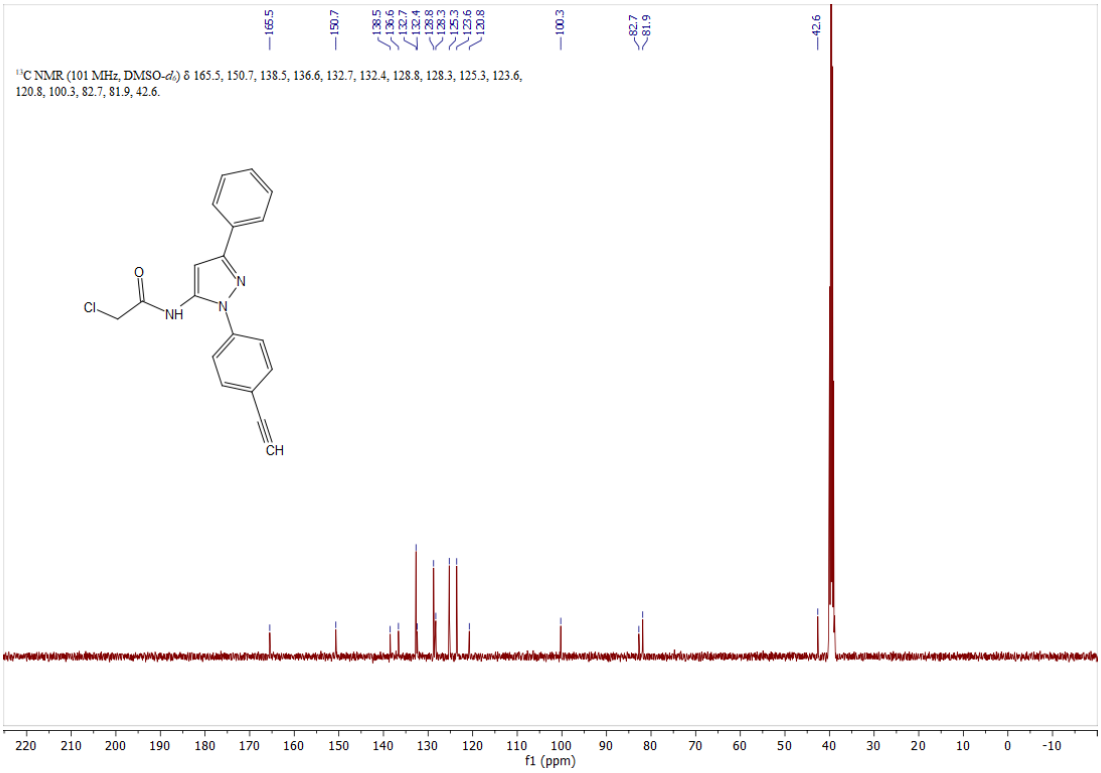

Supplement: Supplementary file 14 — Probes synthesis. [file 41556_2026_1921_MOESM14_ESM.pdf]
