# Supplementary figures and images for: Electrophilic compound screening identifies GPX4-dependent ferroptosis as a senescence vulnerability
Source: Nat Cell Biol. 2026 Apr 24;28(5):915–29. doi: 10.1038/s41556-026-01921-z (PMC13179136; doi:10.1038/s41556-026-01921-z)

# Source Data Figure 3

Figure 3, panel h

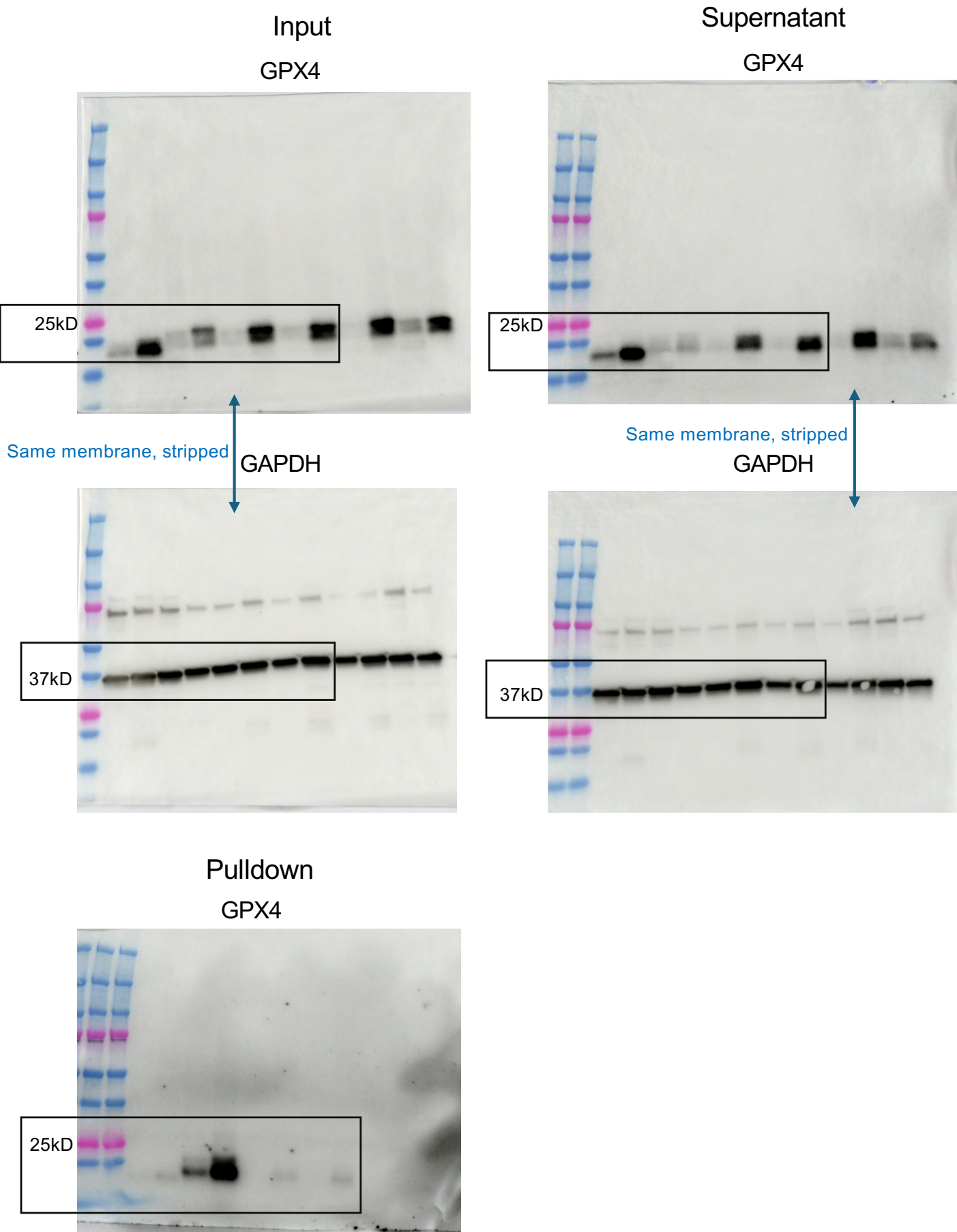

Supplement: Supplementary file 17 — Uncropped western blots. [file 41556_2026_1921_MOESM17_ESM.pdf]

# Source Data ED Figure 5

ED Figure 5, panel d

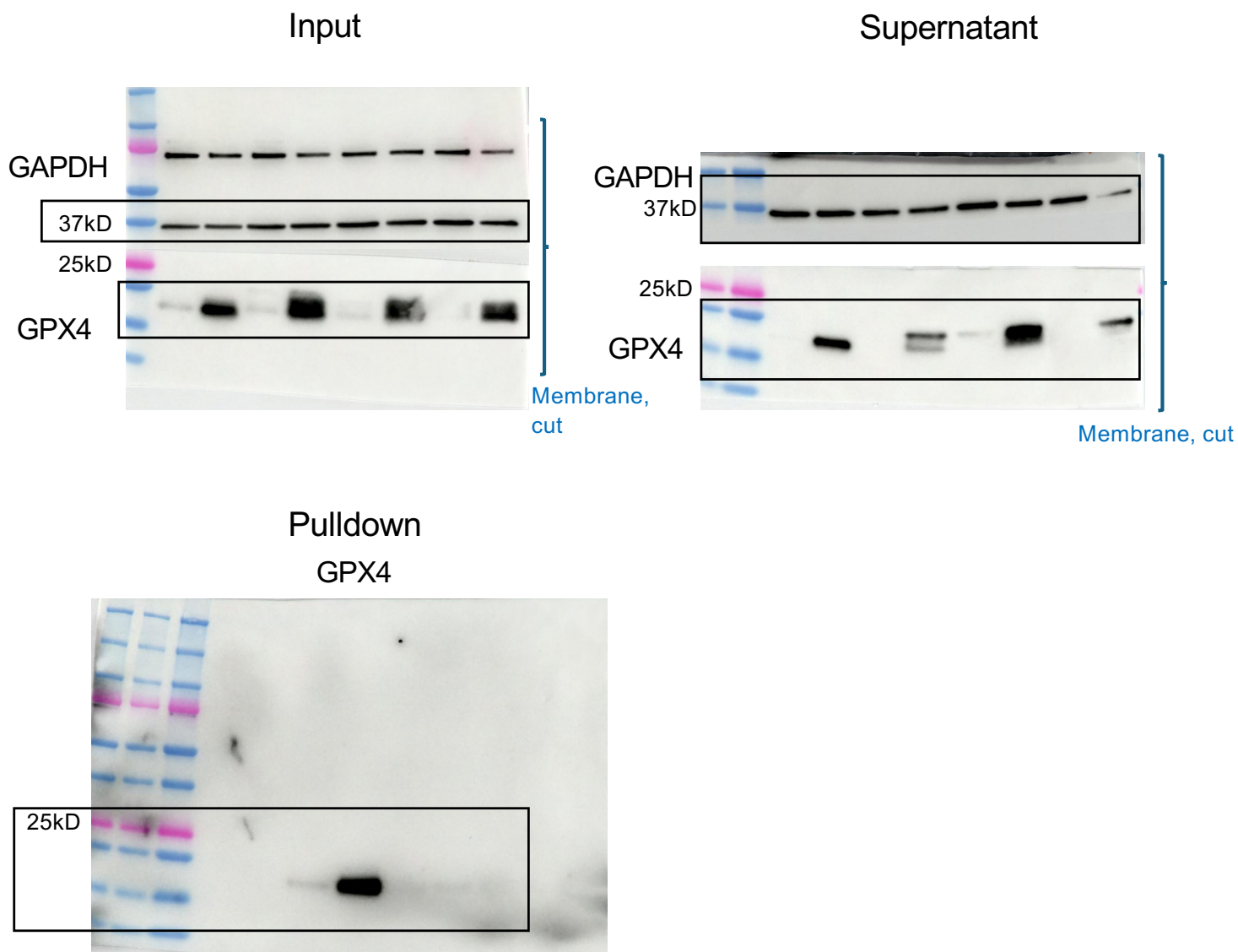

Supplement: Supplementary file 18 — Uncropped western blots. [file 41556_2026_1921_MOESM18_ESM.pdf]

# Source Data ED Figure 6

ED Figure 6, panel f

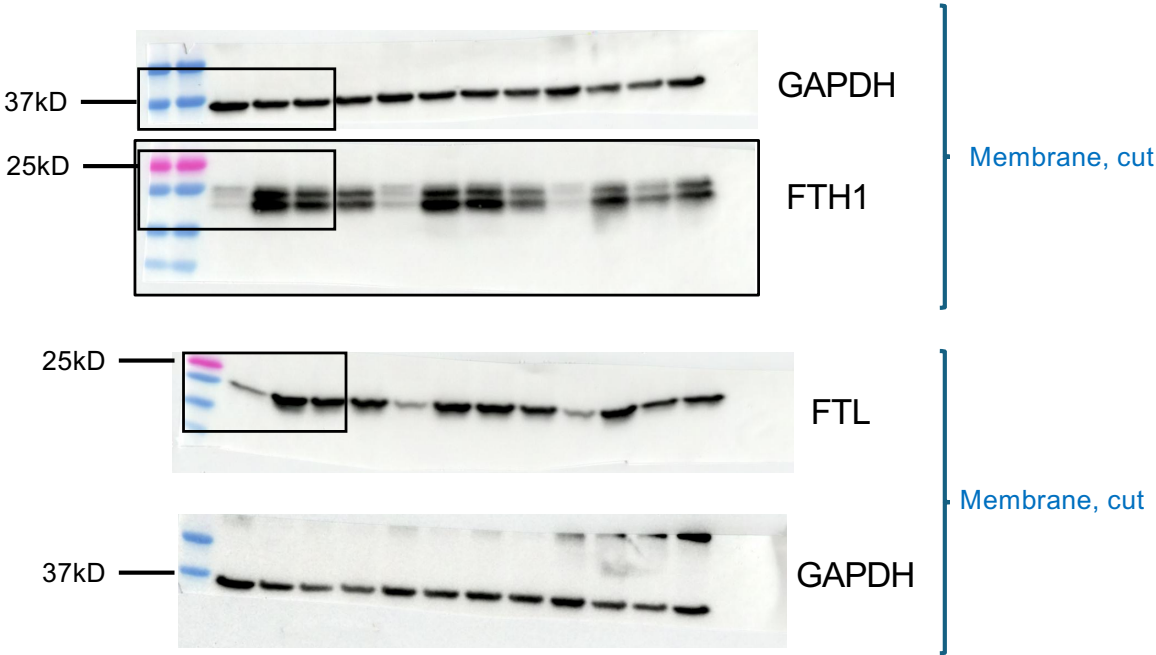

ED Figure 6, panel j

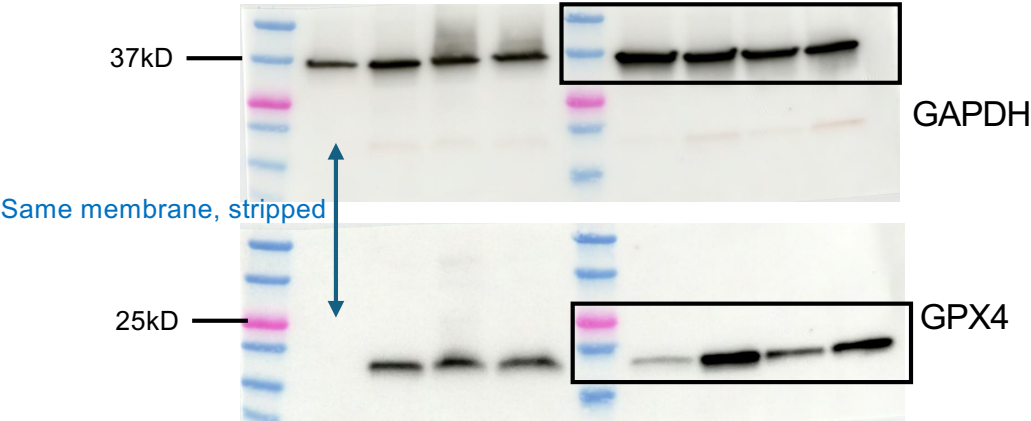

Supplement: Supplementary file 19 — Uncropped western blots. [file 41556_2026_1921_MOESM19_ESM.pdf]

# Source Data ED Figure 7

ED Figure 7, panel e

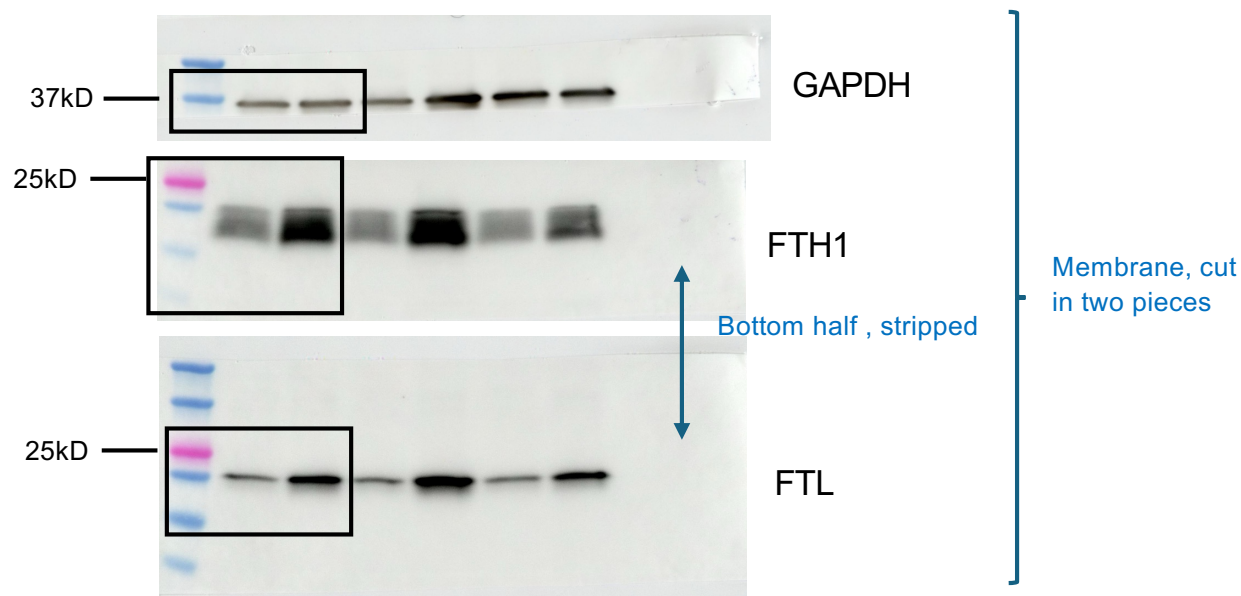

ED Figure 7, panel j

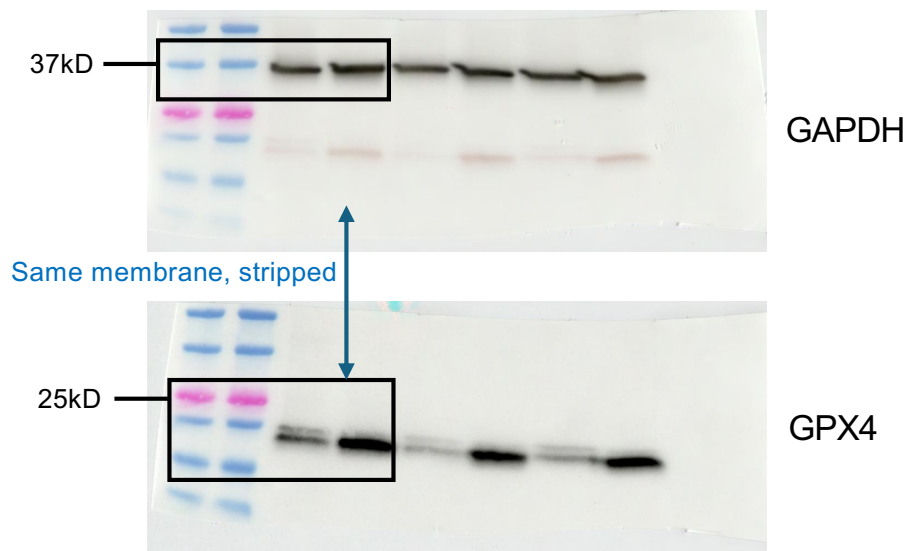

Supplement: Supplementary file 20 — Uncropped western blots. [file 41556_2026_1921_MOESM20_ESM.pdf]
